# Affordability of nutritious foods for complementary feeding in Eastern and Southern Africa

**DOI:** 10.1093/nutrit/nuaa137

**Published:** 2021-03-08

**Authors:** Theresa Ryckman, Ty Beal, Stella Nordhagen, Kudakwashe Chimanya, Joan Matji

**Affiliations:** 1 Center for Health Policy and the Center for Primary Care and Outcomes Research, Department of Medicine, Stanford University School of Medicine, Stanford, California, USA; 2 Global Alliance for Improved Nutrition, Washington DC, USA; 3 Department of Environmental Science and Policy, University of California, Davis, Davis, California, USA; 4 Global Alliance for Improved Nutrition, Geneva, Switzerland; 5 United Nations Children’s Fund, Regional Office for Eastern and Southern Africa, Nairobi, Kenya

**Keywords:** affordability, complementary feeding, dietary diversity, micronutrients, price

## Abstract

Low intake of diverse complementary foods causes critical nutrient gaps in the diets of young children. Inadequate nutrient intake in the first 2 years of life can lead to poor health, educational, and economic outcomes. In this study, the extent to which food affordability is a barrier to consumption of several nutrients critical for child growth and development was examined in Ethiopia, Mozambique, South Africa, Tanzania, Uganda, and Zambia. Drawing upon data from nutrient gap assessments, household surveys, and food composition tables, current consumption levels were assessed, the cost of purchasing key nutritious foods that could fill likely nutrient gaps was calculated, and these costs were compared with current household food expenditure. Vitamin A is affordable for most households (via dark leafy greens, orange-fleshed vegetables, and liver) but only a few foods (fish, legumes, dairy, dark leafy greens, liver) are affordable sources of iron, animal-source protein, or calcium, and only in some countries. Zinc is ubiquitously unaffordable. For unaffordable nutrients, approaches to reduce prices, enhance household production, or increase household resources for nutritious foods are needed.

## INTRODUCTION

Malnutrition remains the leading cause of death and disability among children younger than 5 years.^1^ Although the global prevalence of child stunting (ie, impaired growth and development early in life) has declined from 33% to 22% over the past 2 decades, progress in many countries has been slow, and 1 in 3 children in eastern and southern Africa has stunted growth.^2^ Stunting during the first 2 years of life is linked to lower levels of education and cognitive performance, declines in wages and productivity, and an increased risk of chronic diseases later in life.^3^ Only 2 of 12 countries with available data in eastern and southern Africa are estimated to be on track to meet the 2030 global target of reducing the number of children with stunted growth by 40%.^4^ Deficiencies in key micronutrients among children, which can hamper growth and development and increase the risk of death by impairing immune function, are also a major public health problem.^5^ Iron, vitamin A, and zinc deficiencies are estimated to affect, respectively, 16%, 42%, and 39% of children younger than 5 years in Africa.^6^

Causes of stunting and micronutrient deficiencies include poor nutrient intake and absorption, which can be partially addressed by exclusive breastfeeding in the first 6 months of life followed by the introduction of nutrient-rich, diverse complementary foods with continued breastfeeding from ages 6 to 23 months.^7^ Interventions that improve dietary diversity and complementary feeding practices have been shown to improve child growth.^7^^,^^8^ However, in most countries in eastern and southern Africa, dietary diversity among children is inadequate, with many households relying primarily on lower-cost, nutrient-poor staples to feed their young children. Only 24% of children ages 6–23 months in the region are estimated to receive a diet meeting minimum diversity requirements.^9^ There is evidence of substantial gaps in consumption of multiple micronutrients among children of complementary feeding age (6–23 months) in several countries in the region.^10^

Inadequate complementary feeding practices and low dietary diversity could be driven by several factors, including food availability, physical and temporal access, lack of knowledge, local acceptability of foods for young children, time and convenience, as well as economic barriers. Indeed, evidence indicates that affordability is a key driver of food choice and a barrier to consumption of nutritious foods in many low- and middle-income countries.^11–15^ Researchers recently found that the cost of an adequately nutritious diet exceeded average daily per capita income for more than half of households in sub-Saharan Africa.^15^ Results of cost-of-diet analyses conducted in Mozambique and Tanzania similarly indicated that purchasing a cost-optimized nutritious diet would exceed daily food expenditures for more than half of households.^13^^,^^14^

Although affordability is a known driver of poor dietary diversity in Eastern and Southern Africa, to date there has been little evidence on which nutrients present the greatest affordability barriers and which foods are the most affordable sources of those nutrients. Because interventions to improve complementary feeding practices, especially market-based interventions, often focus on specific foods, identifying the lowest-cost options to fill key nutrient gaps is crucial. It is also essential to examine to what extent these foods are currently affordable to target populations; in both urban and rural areas, poorer households tend to have higher prevalence of stunting and micronutrient deficiencies. Furthermore, identifying those nutrients and foods that are relatively less affordable can inform more targeted research on interventions and policies to improve affordable access to those foods, for example, through market interventions to reduce prices or agricultural interventions to enhance productivity. Past studies have analyzed the cost of an entire food basket or diet^15–17^ or assessed the relative cost of foods on the basis of their caloric content^11^; however, interpreting affordability in the context of specific foods and nutrients remains challenging.

In this study, we combined country-specific data on food consumption, expenditures, and prices with nutrient gap assessments and food composition data to assess current food consumption and identify the least costly foods to fill priority nutrient gaps in 6 countries in eastern and southern Africa. This study complemented a concurrent study on nutrient and food affordability in South Asia that used similar methods.^18^ This methodology can be adapted and used to provide clearer metrics of food affordability for children of complementary feeding age (and other populations) across multiple countries and world regions.

## METHODS

This analysis was focused on 6 countries: Ethiopia, Mozambique, South Africa, Tanzania, Uganda, and Zambia. These countries were chosen because they represent diversity (in economic characteristics and food systems) within the region and because political actors and other key stakeholders in these countries expressed interest in generating evidence on how to improve complementary feeding. The analysis consisted of 3 major steps. First, the study assessed available evidence on nutrient gaps in each country. Next, country survey data and food composition tables were used to identify locally available foods that could fill nutrient gaps and we calculated associated portion sizes required to meet nutrient needs from complementary foods for children ages 6–23 months. Finally, we assessed current household consumption of and expenditure on these foods, estimated the cost of purchasing portion sizes for each food, and compared this cost to current household food expenditures.

### Nutrient gap assessment

We drew upon comprehensive micronutrient gap assessments that were conducted before this analysis.^10^ These assessments synthesized country-specific evidence on deficiencies and prevalence of inadequate intakes or availability of 11 micronutrients commonly lacking in young children’s diets (namely, iron, zinc, vitamin B_12_, calcium, vitamin A, folate, iodine, vitamin B_1_, niacin, vitamin B_6_, and vitamin C).^19^ For each country, all micronutrients with evidence of a moderate to high level of certainty of a moderate to high burden micronutrient gap among children of complementary feeding age were included, based on preliminary micronutrient gap assessments, and then additional micronutrients were added, using these same criteria, on the basis of the finalized assessment methods and their corresponding results.^10^^,^^20^ Thus, micronutrients included in the analysis vary by country, depending on each country’s specific micronutrient gaps. Iodine was omitted from the analysis because iodine content in foods depends heavily on local soil conditions, and salt iodization is an effective and cost-effective strategy to ensure adequate iodine intake.^21^ More details on the nutrient gap assessment methods and findings are available elsewhere.^10^^,^^20^

In addition to these micronutrients, for each country, we also analyzed the affordability of foods that could fulfill daily protein requirements. The analysis focused only on animal sources, because plant sources of protein typically do not include all essential amino acids critical for child growth and development, even though they can be strategically combined to improve the completeness of the amino acid profile.^22^

### Data, food selection, and portion-size calculations

Foods were selected on the basis of nutrient content and local availability. We identified possible foods to analyze using country-specific data on household food consumption and food prices. These sources included household consumption and expenditure surveys and food price data released regularly by national bureaus of statistics or similar governmental bodies. All surveys included household-level data on weekly or biweekly expenditures on a wide range of foods; most also included community-level data on the prices of these foods (see Table S1 in the Supporting Information online). All surveys were also nationally representative and most covered all months of the year and were designed to be representative at the level of state, province, or district (Table S1 in the Supporting Information online).

For each country, foods were selected on the basis of whether they met 3 criteria: (1) the foods could meet 50% of nutrient needs, based on local, regional, and US Department of Agriculture food composition tables,^23–29^; (2) there were adequate data on food prices; and (3) there were adequate data on current household consumption. We used 50% of daily requirements as a target for individual foods because nutrient requirements are met from a combination of foods, and we used requirements specific to intake of complementary foods, assuming a proportion of requirements will be met through breast milk or formula (Table S2 in the Supporting Information online). Criterion 3 was included to increase the likelihood that the foods considered would be locally available and acceptable.

In some cases, variations on the specific food captured in the price and consumption data were analyzed. For example, beef (flesh meat), chicken (flesh meat), beef liver, and chicken liver were included in the analysis for all countries. Beef liver and chicken liver have very high nutrient densities, but consumption and price data on liver specifically were unavailable for several countries. In these cases, price data for beef and chicken, respectively, were used. For 3 food categories that include several varieties that can vary substantially in both nutrient content and prices (namely, dark-green leafy vegetables, legumes, and small fish), we used median nutrient densities and prices across several foods in the analysis (Tables S3 and S4 in the Supporting Information online). Additional details are provided in the Supporting Information online.

We calculated daily edible portion sizes and purchasable quantities required to meet 50% of daily protein and micronutrient requirements from complementary foods for each selected food-nutrient combination, using nutrient density and refuse data from the food composition tables,^23–30^ the proportion of each nutrient needed from complementary feeding,^31^ cooking yield,^32^^,^^33^ and reference-nutrient intake (Tables S2 and S5–S7 in the Supporting Information online).^34–38^ Alternate nutrient density and refuse assumptions were explored for certain foods in sensitivity analyses. The analysis did not include foods for which daily portion sizes were too large to be realistically consumed, although there is surely considerable variability in young children’s desire for and ease of consuming different foods.

We also analyzed the cost to meet total energy requirements from complementary foods (450 kcal/day) for children 6–23 months old for nutritious foods above a minimum energy density (0.8 kcal/g for solid foods),^34^ after accounting for cooking yield and refuse, when applicable. In addition, for all countries the cost per 450 kcal of nutrient-rich foods was compared to the cost of unfortified maize flour, a nutrient-poor but more affordable and widely consumed staple. Prices were converted to 2018 US dollars and 2018 international dollars, using exchange and inflation rates from the World Bank and the International Monetary Fund.^39^^,^^40^

### Cost estimation and affordability analysis

We used the same country-specific data sources on expenditures and prices to estimate the cost and affordability of the selected foods and nutrients. First, surveyed households with children of complementary feeding age (often simplified to children younger than 2 years, because data on household members’ age in months was not always available) were identified. We analyzed household consumption and expenditure patterns, broadly and for the selected foods, and calculated the cost of each food by multiplying the purchasable quantities by country-specific prices. When possible, local price data spanning multiple time periods were used, enabling us to match prices to a household’s subnational region, rural or urban setting, and the month when that household was surveyed.

The cost of each food was compared to total adjusted household food expenditure, which includes a household’s food purchases as well as the value of food consumed from own production and in-kind sources. Household food expenditure was adjusted by the number of adult equivalents (AEQs) in the household, as has been done previously in analyses of household dietary intake.^41^^,^^42^ Adjusting for AEQs, which are an estimate of each household member's proportional energy requirements relative to that of an adult, allows the analysis to incorporate the number of people in the household (given equal expenditures, larger households can afford to shift expenditures less because resources are spread across more people) and their relative food needs (young children require less food than adults). Food expenditure was chosen as the comparison because it is a proxy for household resources available for food and because data on other possible comparators, such as household income, are not readily available for many low- and middle-income countries (and were not available in the data).^43^ The relationship between household food expenditure per AEQ and several food-security indicators was tested in Tanzania and South Africa, where surveys included a food-security module. More details on how the affordability analysis was conducted for each country are available in the Supporting Information online.

To assess inequality in food affordability within countries, subgroup analyses by food expenditure per AEQ quintile and rural or urban residence were also conducted. The base case analysis does not account for current household consumption of a food, but this was explored in sensitivity analysis.

Although comparing the cost of nutritious foods to household food expenditure provides insight on the relative affordability of different foods, it is difficult to interpret the extent to which each food is affordable in absolute terms, because the concept is not clearly defined for individual foods (E Djimeu Wouabe, unpublished data). To help answer this question, we defined 10% as a reasonable threshold for affordability and assessed whether the average cost of foods was above or below 10% of household food expenditure per AEQ. In initial analyses for these 6 countries and for 3 countries in South Asia,^18^ we found that households tend to spend <5% of resources on single foods, apart from non-nutritious staples, making 10% a somewhat conservative but reasonable upper bound on affordability.

### Average share of micronutrient requirements

Because most foods provide > 1 nutrient, we also assessed foods in terms of their affordability for meeting several micronutrient needs in combination. We developed a metric, average share of micronutrient requirements, based on previous advancements to profile foods in terms of their micronutrient content and to quantify the adequacy of an individual’s micronutrient intake.^44^^,^^45^ The average share of requirements for a given portion size of a particular food was calculated as the average proportion of daily requirements (capped at 100% for each nutrient) from complementary foods for a prespecified set of micronutrients that are met by consuming the specified quantity of that food (see Figures S1 and S2 in the Supporting Information online). To allow for comparability across countries, micronutrients were chosen that were included in the by-nutrient affordability analysis for at least 1 country: iron, vitamin A, zinc, calcium, folate, and vitamin B_12_.

We calculated the weekly portion sizes required to achieve an average of one-third of requirements, which corresponds to a portion size providing somewhere between 100% of weekly requirements for 2 micronutrients and one-third of weekly requirements for all 6 micronutrients (Figure S3 in the Supporting Information online). For those foods for which this portion size was ≤100 g per day (chosen because it is a reasonable complementary feeding meal size), we calculated the cost of purchasing the portion sizes as a share of weekly household food expenditure per AEQ. The same data and assumptions for the daily requirements of a food that must be met through complementary feeding, nutrient densities, refuse, and cooking yield were used as in the by-nutrient affordability analysis. Although we focused on conclusions about relative affordability that could be drawn from this analysis, because we assessed an average of one-third of requirements, one-third of weekly household food expenditure per AEQ was also established as a rough absolute affordability threshold.

### Statistical analysis

All data cleaning and statistical analysis were conducted in Stata 15.^46^ The *svy* family of commands in Stata was used to account for the complex sampling design (including weighting, clustering, and stratification) used for each of the country surveys. The *svy* package calculates weighted estimates and robust standard errors that account for clustering and stratified sampling. The results thus can be interpreted as population-representative means (nationally or for subgroups), with 95% CIs around the means, calculated assuming the data follow a normal distribution parameterized by these means and the linearized robust standard errors estimated from the data. Specifically, confidence intervals in the results incorporated within-country variability in prices and household food expenditures but not uncertainty around nutrient densities and refuse (these were explored separately via sensitivity analysis).

## RESULTS

### Nutrient gaps

For each country, there was substantial evidence of complementary feeding gaps in ≥2 micronutrients (Table 1). On the basis of pre-established inclusion criteria (including certainty of evidence and micronutrient-gap burden level) and results from preliminary and final micronutrient gap assessments, iron and vitamin A were included in the analysis for all 6 countries, calcium for 5 countries, zinc for 2 countries (Ethiopia and Zambia), and folate and vitamin B_12_ were included only for Zambia.^10^

**Table 1:** Micronutrients analyzed on the basis of preliminary and final gap assessments in 6 countries in eastern and southern Africa

Country	Iron	Vitamin A	Calcium	Zinc	Folate	Vitamin B_12_	Total
Ethiopia							4
Mozambique							2
South Africa							2
Tanzania							3
Uganda							2
Zambia							6
Total	6	6	5	2	1	1	

### Household consumption and expenditure patterns

**Figure 1:**
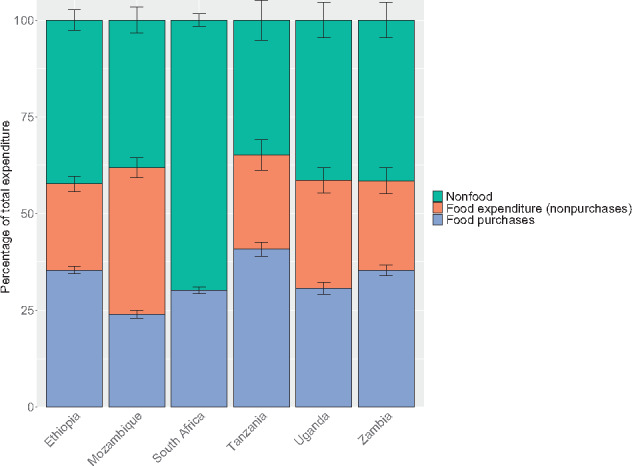
**Total household food and nonfood expenditures**. Breakdowns of food expenditure by purchases vs own production or other sources were not available for South Africa. However, purchases were thought to compose the vast majority of total household food expenditure, given low levels of food consumption from own production in the country. For all countries, only households with children of complementary feeding age are shown, yielding sample sizes of 5808 households in Ethiopia, 9441 in Mozambique, 3303 in South Africa, 1062 in Tanzania, 728 in Uganda, and 2054 in Zambia. Error bars represent 95%CIs.

In 5 of the 6 countries analyzed (Ethiopia, Mozambique, Tanzania, Uganda, and Zambia), food expenditures accounted for 58%–65% of total expenditure, on average, whereas nonfood expenditures made up the remaining 35%–42% (Figure 1). Food made up a much smaller share of total household spending—only 30% (95%CI 29%–31%)—in South Africa. In Ethiopia, Tanzania, Uganda, and Zambia, the majority of food expenditure came from purchases (between 54% and 65%, on average). In Mozambique, more food expenditure came from own production. Even in rural areas, households obtained, on average, approximately half of food consumption from purchases, except for those in Mozambique, where the proportion was closer to 30% (95%CI 29%–32%) (Figure S4 in the Supporting Information online). Analyzing expenditures by quintile revealed stark inequalities between households; households in the lowest food expenditure quintile per AEQ spent 68%–90% less than the highest-quintile households and 48%–77% less than the average household (Figure S5 in the Supporting Information online).

**Figure 2:**
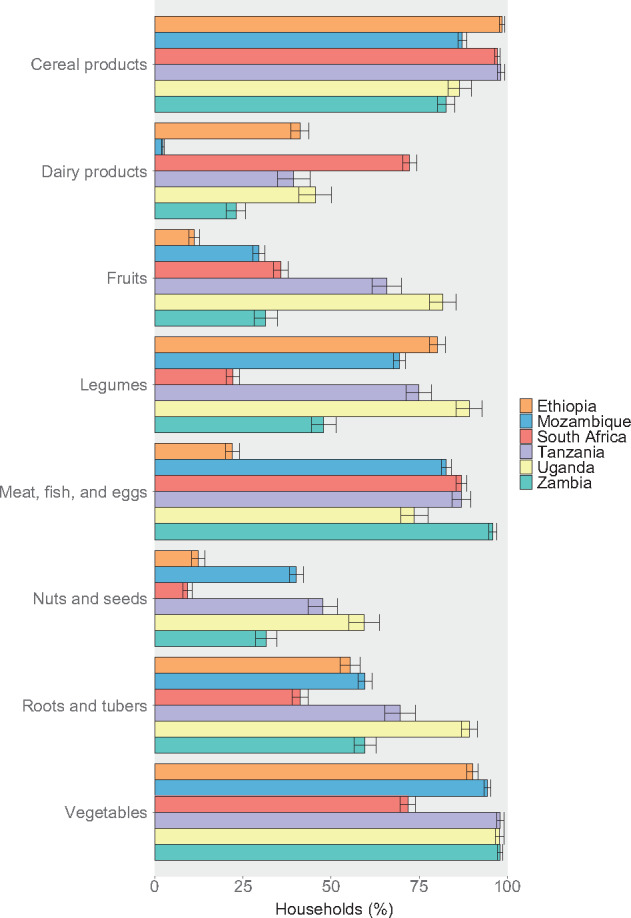
**Household consumption of key food groups**. Other food groups, including sugar and other sweets; oils and other fats; salt, other spices, and condiments; beverages; and foods eaten outside of the home were omitted for brevity. Surveys covered household consumption over the past week, except for South Africa and Zambia, which covered the past 2 weeks. For all countries, only households with children of complementary feeding age are shown, yielding sample sizes of 5808 households in Ethiopia, 9441 in Mozambique, 3303 in South Africa, 1062 in Tanzania, 728 in Uganda, and 2054 in Zambia. Error bars represent 95%CIs.

**Figure 3:**
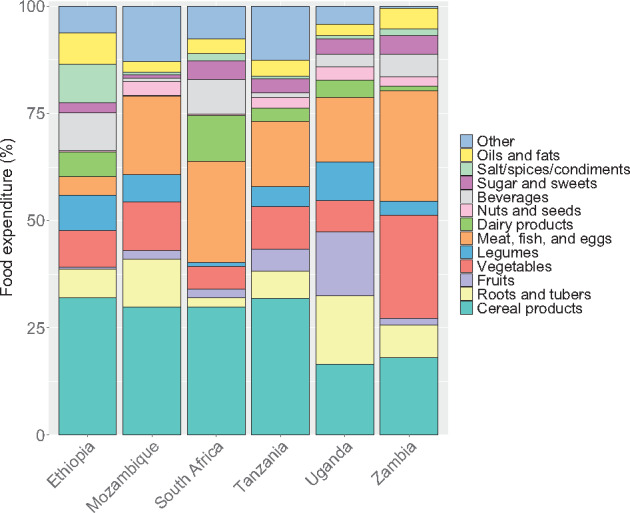
**Household expenditure by food group.** Expenditure includes value of consumption from all sources (ie, purchases, own production, and in kind). Only households with children of complementary feeding age are shown, yielding sample sizes of 5808 households in Ethiopia, 9441 in Mozambique, 3303 in South Africa, 1062 in Tanzania, 728 in Uganda, and 2054 in Zambia. Error bars represent 95%CIs.

Household consumption of different food groups varied by country (Figure 2). In all countries, most households consumed cereal products and vegetables during the survey period (the last 1 or 2 weeks; see Table S1 in the Supporting Information online), and cereal products accounted for at least 15% of average food expenditure in all 6 countries (Figure 3). In Zambia, 24% of food expenditure went toward vegetables, on average, whereas this figure was only 5%–11% in the other countries. Foods from the meat, fish, and eggs group were also consumed by >70% of households in all countries and tended to be the second-highest expenditure food group, except in Ethiopia, where <25% of households consumed these foods. Cereal products, vegetables, and meat, fish, and eggs made up more than half of average food expenditure in all countries except Ethiopia and Uganda. For other foods groups, consumption across countries was more varied. Uganda had the highest proportion of households consuming fruits, legumes, nuts and seeds, and roots and tubers, and the second-highest proportion consuming dairy products. In South Africa, 72% of households consumed dairy products, whereas only 2% of households in Mozambique consumed dairy. Consumption and expenditure patterns were generally similar across rural and urban areas within a country (Figures S6 and S7 in the Supporting Information online). The proportion of households consuming different food groups tended to increase as household food expenditure increased (Figures S8–S10 in the Supporting Information online); these trends were especially noticeable for animal-source foods, which were consumed more by higher-quintile households.

### Selected foods to meet nutrient needs

For each country, surveys included several foods that could meet protein or micronutrient needs, were consumed in the country, and for which price data were available (Table 2). Many of the same foods were selected as possibilities to fill the same nutrient gap in multiple countries. In addition, some foods could be used to meet multiple nutrient needs. For instance, dark-green leafy vegetables contain iron, vitamin A, calcium, and folate; beef is high in protein, iron, and zinc; and small dried fish are a good source of protein, iron, vitamin A, calcium, zinc, and vitamin B_12_. Almost all foods analyzed could fill at least 2 nutrient gaps.

**Table 2:** **Selected foods to meet animal-source protein and micronutrient needs** ^a^

**Ethiopia** Protein: beef, chicken, eggs, cottage cheese, fresh milk Iron: chicken liver, beef liver, dark-green leafy vegetables, beef, legumes Vitamin A: beef liver, chicken liver, carrots, pumpkin, dark-green leafy vegetables, eggs, fresh milk, cottage cheese Calcium: fresh milk, dark-green leafy vegetables Zinc: beef, beef liver, chicken liver, groundnuts, chicken, legumes, eggs, fresh milk
**Mozambique** Protein: small dried fish, beef, chicken, fresh or frozen fish, eggs, fresh milk Iron: chicken liver, small dried fish, beef liver, dark-green leafy vegetables, beef, legumes Vitamin A: beef liver, chicken liver, orange-fleshed sweet potatoes, small dried fish, dark-green leafy vegetables, eggs, mango, fresh milk, fresh or frozen fish
**South Africa** Protein: beef, chicken, small tinned fish, eggs, sour milk or yogurt, fresh milk Iron: chicken liver, beef liver, dark-green leafy vegetables, small tinned fish, beef, legumes Vitamin A: beef liver, chicken liver, carrots, pumpkin, dark-green leafy vegetables, eggs, mango, fresh milk, small tinned fish, sour milk or yogurt Calcium: small tinned fish, fresh milk, dark-green leafy vegetables
**Tanzania** Protein: small dried fish, beef, chicken, fresh or frozen fish, eggs, fresh milk Iron: chicken liver, small dried fish, beef liver, dark-green leafy vegetables, beef, legumes Vitamin A: beef liver, chicken liver, carrots, dark-green leafy vegetables, small dried and tinned fish, eggs, mango, papaya, fresh milk, fresh or frozen fish Calcium: small dried fish, fresh milk, dark-green leafy vegetables
**Uganda** Protein: small dried fish, beef, chicken, fresh or frozen fish, eggs, fresh milk Iron: chicken liver, small dried fish, beef liver, dark-green leafy vegetables, beef, legumes Vitamin A: beef liver, chicken liver, carrots, small dried fish, pumpkin, dark-green leafy vegetables, eggs, mango, papaya, fresh milk, fresh or frozen fish Calcium: small dried fish, fresh milk, dark-green leafy vegetables
**Zambia** Protein: small dried fish, beef, chicken, fresh or frozen fish, eggs, sour milk or yogurt, fresh milk Iron: chicken liver, small dried fish, beef liver, dark-green leafy vegetables, beef, legumes Vitamin A: beef liver, chicken liver, carrots, small dried fish, pumpkin, dark-green leafy vegetables, eggs, fresh milk, fresh or frozen fish, sour milk Calcium: small dried fish, sour milk or yogurt, fresh milk, dark-green leafy vegetables Zinc: small dried fish, beef, beef liver, chicken liver, groundnuts, chicken, legumes, eggs, sour milk or yogurt, fresh milk Folate: chicken liver, beef liver, groundnuts, legumes, okra, eggs, dark-green leafy vegetables, oranges, bananas Vitamin B_12_: beef liver, chicken liver, small dried fish, fresh or frozen fish, beef, eggs, fresh milk, sour milk or yogurt

a Foods are ordered from highest to lowest nutrient density within each nutrient category.

**Figure 4:**
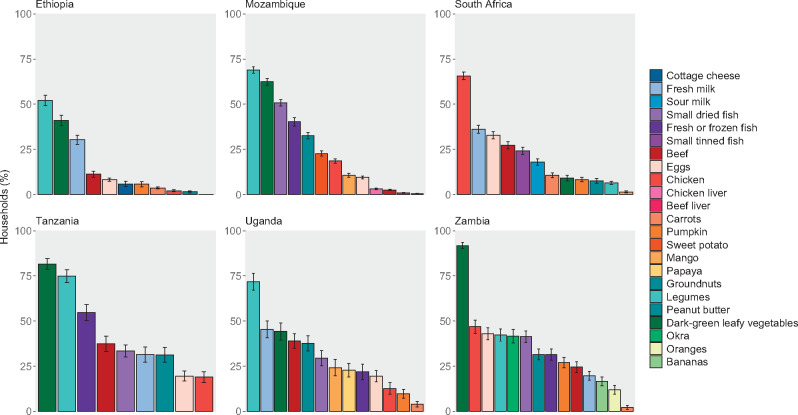
**Current consumption of selected nutritious foods.** Surveys covered household consumption over the past week, except for South Africa and Zambia, for which the past w weeks’ consumption was covered. Only households with children of complementary feeding age are shown, yielding sample sizes of 5808 households in Ethiopia, 9441 in Mozambique, 3303 in South Africa, 1062 in Tanzania, 728 in Uganda, and 2054 in Zambia. Error bars represent 95%CIs.

**Figure 5:**
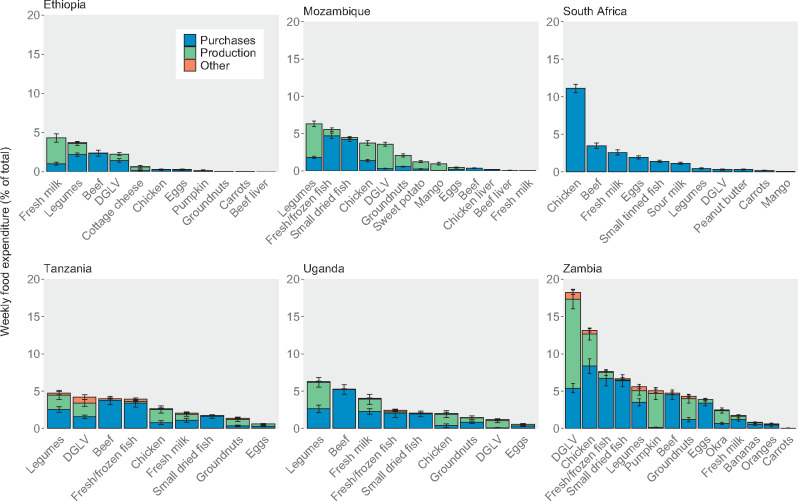
**Current expenditure from purchases, own production, and other sources on selected nutritious foods.** Breakdowns of food expenditure by type were not available for South Africa. Mozambique’s survey only covered expenditures from purchases and own production, not other sources. Only households with children of complementary feeding age are shown, yielding sample sizes of 5808 households in Ethiopia, 9441 in Mozambique, 3303 in South Africa, 1062 in Tanzania, 728 in Uganda, and 2054 in Zambia. Error bars represent 95%CIs. DGLV, dark-green leafy vegetables.

Although many of the selected foods were similar across countries, consumption of and expenditure on these foods varied (Figures 4 and 5), as did food prices. Legumes and dark-green leafy vegetables were among the most commonly consumed foods in all countries except South Africa. These 2 foods were also more frequently consumed from own production than other foods analyzed. Milk was also consumed by approximately 30%–40% of households in Ethiopia, South Africa, Tanzania, and Uganda but was consumed less frequently in Zambia and was rarely consumed in Mozambique. Countries varied in the consumption of other animal-source proteins, including chicken, beef, fish, and eggs, each of which was frequently consumed in some countries and rarely consumed in others.

Very few foods accounted for >10% of household food expenditure (only chicken in South Africa and Zambia and dark-green leafy vegetables in Zambia). Across all countries, most households spent <5% of resources for food on each of the nutritious foods in this analysis. Foods that accounted for the greatest share of food expenditure included those that were most frequently consumed (eg, dark-green leafy vegetables and legumes) and those that were highest priced (eg, chicken, beef, and fish). Within each country, rural and urban households consumed many of the same foods, with rural households sometimes consuming a specific food less frequently than urban households (Figures S11 and S12 in the Supporting Information online). Households in the higher quintiles of food expenditure per AEQ consumed many foods more frequently and with higher expenditures, especially more expensive animal-source foods (eg, beef, chicken, fish) (Figures S13 and S14 in the Supporting Information online).

### Affordability of selected nutritious foods

**Figure 6:**
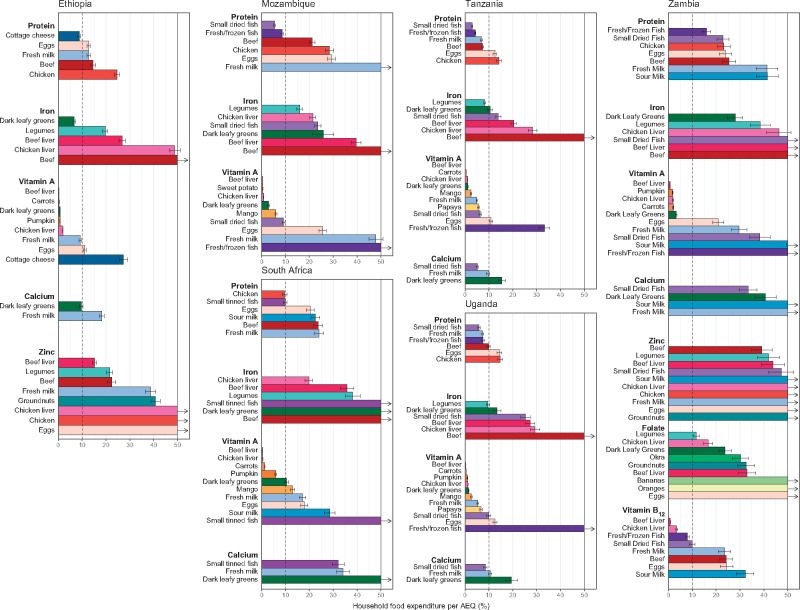
**Portion-size cost as a share of total household food expenditure per adult equivalent.** The *y*-axis was truncated at 50%, but the costs of some foods exceeded 50% of household food expenditure per adult equivalent (AEQ); these foods are designated with vertical arrows indicating that the bar continues vertically beyond the scale of the graph. Regional price data were not available for Zambia and thus the CIs shown on the Zambia panel do not incorporate geographic price variation. Only households with children of complementary feeding age are shown, yielding sample sizes of 5808 households in Ethiopia, 9441 in Mozambique, 3303 in South Africa, 1062 in Tanzania, 728 in Uganda, and 2054 in Zambia. Error bars represent 95%CIs

The main affordability analysis results are shown in Figure 6. Vitamin A is the most affordable nutrient, with several foods available in all countries that could meet young children’s nutrient requirements and cost <5% (or even <1%) of current household food expenditure per AEQ. These foods include dark-green leafy vegetables; orange-fleshed vegetables, roots, and tubers such as carrots, pumpkin, and sweet potatoes; beef liver; chicken liver; and orange-fleshed fruits such as mango and papaya. Dark-green leafy vegetables are also among the most affordable sources of iron, except in South Africa, where they are relatively higher priced, and they are a somewhat affordable source of calcium in Ethiopia.

On the other hand, there are generally only 1 or 2 foods in each country that could meet half of animal-source protein, iron, and calcium needs and cost <10% of household food expenditure per AEQ. These foods vary by country, but, in addition to dark-green leafy vegetables, include small dried or tinned fish (protein, calcium), legumes (iron), dairy products such as milk or cottage cheese (calcium, protein), and fresh or frozen fish (protein). In Mozambique, South Africa, and Zambia, all foods that could meet iron requirements (and calcium requirements for South Africa and Zambia) exceed the 10% expenditure threshold. For Ethiopia and Zambia, the 2 countries for which zinc was assessed, all foods that could meet 50% of zinc needs would exceed 15% of household food expenditure per AEQ.

**Figure 7:**
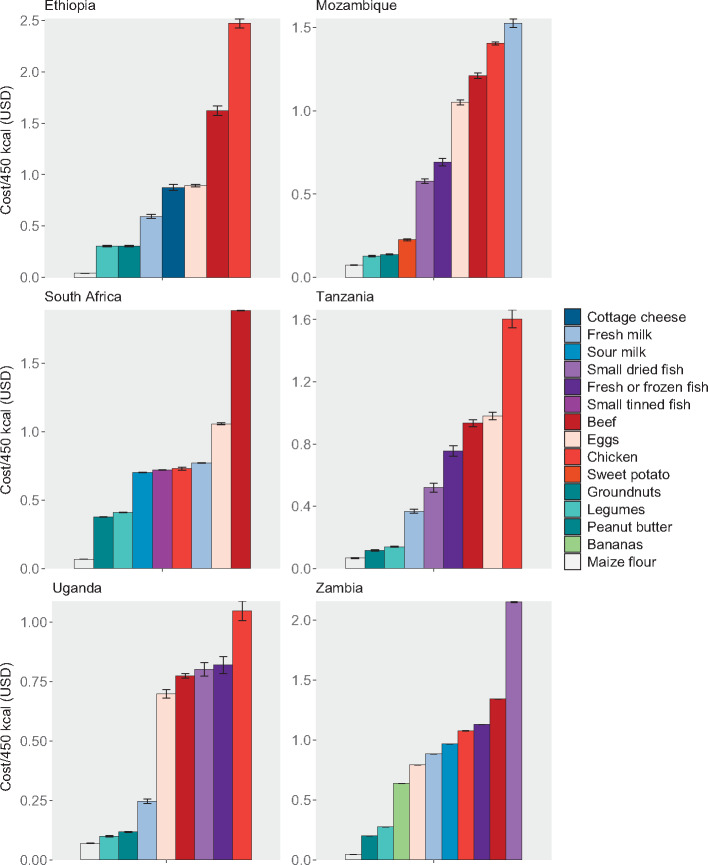
**Food cost per 450 kcal**. Regional price data were not available for Zambia; thus, the costs shown here only incorporate small temporal changes in price over the survey period. Only households with children of complementary feeding age are shown, yielding sample sizes of 5808 households in Ethiopia, 9441 in Mozambique, 3303 in South Africa, 1062 in Tanzania, 728 in Uganda, and 2054 in Zambia. Error bars represent 95%CIs

Even the most affordable nutritious foods tend to cost between 2 and 10 times more per kilocalorie than maize flour, a relatively inexpensive but nutrient-poor staple, signifying the challenge many households may face in purchasing more nutritious items (Figure 7). Notably, these foods are relatively low-cost sources of some nutrients but high-cost sources of others; for example, small dried fish are often a more affordable source of protein and calcium but a less affordable source of iron and vitamin A. Chicken (not including liver), beef (not including liver), and eggs, which are relatively higher priced, tend to be unaffordable sources of each of the 6 nutrients individually and were also the most expensive foods per kilocalorie, with some exceptions (namely, chicken in South Africa and beef in Tanzania). Results organized by food are shown in Figure S15 and by different affordability thresholds are listed in Table S8 (both in the Supporting Information online).

**Figure 8:**
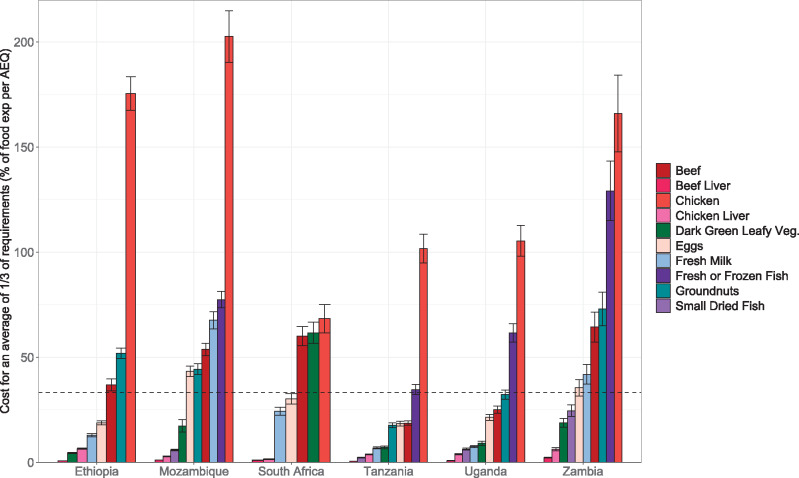
**Cost of achieving an average of one-third of micronutrient requirements, as a share of total household food expenditure per adult equivalent (AEQ)**. Regional price data were not available for Zambia; thus, the CIs shown for Zambia do not incorporate geographic price variation. Only households with children of complementary feeding age are shown, yielding sample sizes of 5808 households in Ethiopia, 9441 in Mozambique, 3303 in South Africa, 1062 in Tanzania, 728 in Uganda, and 2054 in Zambia. Error bars represent 95%CIs

Findings from the average share of micronutrient requirements analysis indicated that several animal-source foods, including small dried fish, beef liver, chicken liver, milk, and eggs, appeared more affordable when their joint contributions toward requirements for several micronutrients were considered (Figure 8). However, for the most part, beef and especially chicken (not including liver) are still relatively expensive sources of the nutrients analyzed in this study, even when assessing multiple nutrients together (except beef for the latter in Tanzania, Uganda, and, to some extent, Ethiopia). The consideration of a food’s combined nutrient composition also demonstrates the advantages of some animal-source foods. A single 100-g (or smaller) portion of several animal-source foods could achieve an average of one-third of requirements (indicating that between 100% of needs for 2 micronutrients and one-third of needs for all 6 micronutrients are met), whereas dark-green leafy vegetables and groundnuts were the only plant-source foods analyzed that met this criterion (Table 3), although legumes were relatively close, with 100 g of legumes achieving an average of 29% of requirements (Figure S3 in the Supporting Information online).

**Table 3:** **Average share of micronutrient requirements portion sizes** ^a^

Food	Daily edible portion size required to achieve an average of one-third of requirements (g)
Beef liver	1
Chicken liver	3
Small dried fish	6
Beef	27
Eggs	35
Dark-green leafy vegetables	72
Chicken	83
Fresh milk	95
Fresh or frozen fish	98
Groundnuts	99
Yogurt or sour milk	110
Legumes	139
Okra	161
Papaya	164
Mango	174
Carrots	258
Pumpkin	319

a Foods with a portion size >100 g were not included in the average share of requirements analysis.

Foods tend to be less affordable in rural areas, which reflects lower average food expenditures among rural households rather than it does price differences (Figures S16–S18 in the Supporting Information online). Results stratified by food expenditure quintile (Figures S19–S21 in the Supporting Information online) demonstrate the greater challenges faced by low-resource households, which tend to be less food secure (Table S9 in the Supporting Information online) and are presumably more economically constrained. Although the cost of several foods (and individual nutrients) fell below 10% of household food expenditure per AEQ on average (Figure 6), and the cost of foods assessed by their combined nutrient contributions fell below one-third of household food expenditure per AEQ on average (Figure 8), these averages mask stark inequalities between households in a country. For the lowest spending 20% of households, the cost of all nutritious foods analyzed by nutrient (except vitamin A, vitamin B_12_ in Zambia, and protein in Tanzania) exceeded 10% of household food spending per AEQ, indicating that affordability likely presents substantial barriers to filling most nutrient gaps among poorer households in all 6 countries (Figure S19 in the Supporting Information online). On the basis of both the by-nutrient affordability analysis and the average share of micronutreint requirements analysis, liver and, in some countries, small dried fish, milk, and/or dark-green leafy vegetables are the most affordable options for low-resource households to fill important micronutrient gaps for young children (Figure S21 in the Supporting Information online).

Households in Zambia also face unique affordability challenges, which are only partly explained by the greater number of nutrients analyzed; even for nutrient gaps present in most countries, foods that could fill these gaps tend to be less affordable (Figure 6). It is difficult to tease apart the extent to which this result is driven by higher food prices or lower household spending; however, in a comparison of food prices converted to international dollars (which convert local currencies into a common currency that accounts for purchasing power differences^47^), prices for most items are not uniquely higher in Zambia (Figure S22 in the Supporting Information online). The bigger culprit is inequality in household spending; lower-spending households in Zambia spend much less on food relative to higher-spending households, or even the average household, than is the case in other countries (Figure S5 in the Supporting Information online). This finding is also evident in Figure S19 in the Supporting Information online; Zambian households in the lowest-spending quintile face far greater affordability barriers than the lowest-quintile households in other countries, whereas affordability results look similar between the highest-spending households in Zambia and the other countries.

### Sensitivity analyses

These results do not account for current household expenditure on a food. However, when the estimated current consumption of a food is incorporated, some foods become slightly more affordable, but the results are not substantially changed, indicating that households are not currently consuming the selected foods in substantial quantities compared with the required portion sizes (Figure S23 in the Supporting Information online).

In some cases, multiple similar foods were placed in a broad category. For example, dark-green leafy vegetables could include spinach, mustard greens, sweet-potato leaves, amaranth, and many others, which can have very different nutrient densities and prices (Tables S3 and S4 in the Supporting Information online). Therefore, average prices and median nutrient densities across several different foods within the dark-green leafy vegetables, legumes, and small fish categories were used. The analysis also explored alternate nutrient densities, which, for the most part, had little effect on overall nutrient affordability in each country or on which foods were the most and least affordable sources of each nutrient (Figures S24 and S25 in the Supporting Information online). The nutrients most affected by this sensitivity analysis were iron (Ethiopia, Mozambique, Tanzania, and Uganda) and calcium (Ethiopia, Tanzania, and Uganda). In Ethiopia, for example, dark-green leafy vegetables are, on average, a somewhat affordable source of iron (costing 10% of household food expenditure per AEQ) but could cost as little as 4% and as much as 23% under a range of plausible nutrient densities.

## DISCUSSION

Although the results of this study varied by country, several overarching conclusions can be drawn. Affordability is unlikely to be a cause of gaps in vitamin A intake (and vitamin B_12_ in Zambia) among children of complementary feeding age in the region. Even households that spend relatively little on food can choose between several foods (orange-fleshed vegetables and fruits, dark-green leafy vegetables, chicken liver, beef liver) that would require them to reallocate <5% (and often <1%) of household food expenditures per AEQ. Greater than 40% of households in all countries except South Africa already consume dark-green leafy vegetables, many from own production, but current consumption levels of orange-fleshed vegetables and liver were generally lower. Many of these foods have low prices, and the required portion sizes are relatively small. Gaps in vitamin A instead may be caused by poor accessibility (these foods may only be available in certain locations or times of year), knowledge (caretakers or the household member primarily responsible for purchasing food may be unaware of the importance of vitamin A consumption or which foods contain vitamin A), desirability, or preferences. Convenience also likely plays a role. Certain foods take more time and attention to prepare or spoil more easily than others, or households may prefer not to purchase food separately for young children, but purchasing a sufficient amount of a food to feed the entire household pushes up the cost considerably. Future work should focus on identifying these barriers and designing policy interventions that can address them. Prevalence of continued breastfeeding is relatively high in eastern and southern Africa^9^ and should be maintained to help ensure intake of key nutrients, such as vitamin A.

Another option to provide young children with additional vitamin A is vitamin A supplementation. Although vitamin A supplementation programs are generally delivered and funded by country governments or development partners, when assessed using the same household affordability metric, vitamin A supplementation would cost between 1% and 3% of food expenditure per AEQ, similar to the cost of liver, dark leafy greens, and orange-fleshed fruits and vegetables while providing close to 100% of vitamin A needs (Tables S10 and S11 in the Supporting Information online). Although vitamin A supplementation could be a similarly or more affordable alternative to obtaining vitamin A through complementary feeding and requires less behavior change on the part of households, regional coverage is only 68% and could decline as many countries move to integrate their campaign-based supplementation programs within routine health services.^48^^,^^49^

For the remaining nutrients, there are generally only 1 or 2 foods that could meet a child’s nutrient needs for <10% of current household food spending per AEQ. These foods vary by country, but, in addition to dark-green leafy vegetables (iron, calcium, vitamin A), they often include small dried or tinned fish (protein, iron, calcium), legumes (iron, zinc, folate; also relatively low cost per kilocalorie), dairy products (calcium, protein), and fresh or frozen fish (protein). Although some of these foods are already frequently consumed in many countries (ie, dark-green leafy vegetables, legumes, milk, small dried fish), sensitivity analyses revealed that current consumption levels are likely too small to meet requirements for these less-affordable nutrients. In several cases (ie, most nutrients in Zambia, zinc in Ethiopia, iron in Mozambique, and calcium and iron in South Africa), there are no foods for which cost falls below the 10% threshold. Furthermore, lower-spending households, which tend to face greater food insecurity, also face greater affordability challenges. In almost all countries, purchasing these nutritious foods would require already resource-constrained households to reallocate >10% of current food expenditures per AEQ. At least 4 foods in each country (especially chicken liver, beef liver, and small dried fish, and also dark-green leafy vegetables, milk, and eggs) may be affordable when their contributions to multiple micronutrient requirements are considered. This analysis highlights promising options for households and for future research but is not a substitution for the affordability analysis by individual nutrient, because constraints on affordability and desirability (as well as food seasonality) will require availability of a larger range of foods. Notably, neither analysis incorporates a food’s content of other nutrients for which there was less evidence of gaps or components beyond nutrients that contribute to health (eg, prebiotics, probiotics, polyphenols, and other beneficial bioactive compounds).

Of the 6 countries analyzed, Zambian households, especially those in the lowest expenditure quintile, appear to face the most urgent affordability barriers. It is perhaps no coincidence that Zambia has evidence of the greatest number of nutrient gaps and also faces the greatest economic obstacles to filling these gaps. Vitamin A supplementation may be a more promising option for filling vitamin A gaps (based on both affordability compared to foods and current coverage levels) in Zambia than in several of the other countries. Additional research on short- and long-term strategies to address nutrient gaps among low-resource households in Zambia should be prioritized.

These findings are consistent with other literature on food affordability in concluding that economic barriers (among others) prevent children from accessing more nutritious diets. In their analysis of relative caloric prices, Headey and Alderman^11^ found that pulses tend to be the cheapest sources of energy in eastern and southern Africa and animal-source foods are among the most expensive, which is consistent with our findings in this study. Similar to this study’s results presented in Table 7, in Tanzania, Masters et al^16^ identified maize as the lowest-cost source of energy, followed by groundnuts and soybeans. However, they also found that beef is much more affordable than the results from our study indicate; this could be due to different assumptions about the cuts of beef being purchased, their nutrient content (eg, fattier cuts have higher energy density, and the analysis assumed beef was approximately 89% lean), refuse, cooking yield, or regional variation in beef prices. Both this analysis and that of Masters et al^16^ also found that energy and calcium are among the most expensive nutrients and vitamin A is among the least expensive in Tanzania. Contrary to our findings, in their affordability analysis of the EAT-*Lancet* reference diet, Hirvonen et al^15^ reported that fruits and vegetables are the most costly dietary component in sub-Saharan Africa. In the present study, we found that several fruits and vegetables (especially dark-green leafy vegetables) are among the least expensive foods to fill several nutrient gaps. Although Hirvonen et al^15^ relied on different price data and their analysis covered many more countries, this difference is also likely due to their analysis of diets that fill all nutrient needs for a 30-year-old woman (rather than a selection of individual nutrient needs for a child 6–23 months old) and their consideration of additional factors in food selection, such as environmental sustainability.

The present study is a valuable addition to the current literature on nutrient affordability. This, and the accompanying analysis for South Asia,^18^ are the first of which we are aware that examined the absolute and relative affordability of several individual nutrients, and foods that are good sources of those nutrients, across multiple countries (E Djimeu Wouabe, unpublished data). We relied on data from nationally representative household surveys and recent local price data, which allowed the analysis to account for regional variation and inequities across households within countries. The integration of data on household consumption, evidence on nutrient intake, and country-specific food composition tables enabled us to choose locally relevant foods that could feasibly be used to fill priority nutrient gaps among children of complementary feeding age. Many of the surveys used are conducted periodically, providing an opportunity to track progress over time. The resulting findings provide robust evidence on the relative affordability of different foods and nutrients in the 6 countries analyzed.

Although the results of this study do not make as clear a determination about the absolute affordability of each food for each nutrient and country, broader conclusions can be drawn by setting a percentage of household food spending per AEQ (10% and 33.3%) as the upper limit of affordability for individual nutrients and micronutrients jointly, respectively. In addition to being based on current expenditure patterns, the rationale for choosing 10% is that it is reasonable to assume that most households (except those experiencing extreme food insecurity) could afford to shift 10% of current food expenditure to different foods without pulling considerably from nonfood expenditures, which are likely meeting basic needs for many households. The 33.3% threshold was chosen to match the average percentage of requirements that could be achieved (also 33.3%). This analysis was also based on the affordability of portion sizes that provide 50% of requirements for a nutrient, assuming the other 50% will come from other foods that are currently part of the diet, but this may not be the case for all children. Although these decisions were somewhat arbitrary, they do not affect the affordability of the different foods and nutrients relative to each other and they allow for a clearer determination of what foods are affordable or unaffordable, which is useful for informing nutrition policy and programming. Challenges in definitively classifying a food as affordable are common to much of the food and nutrient affordability literature (E Djimeu Wouabe, unpublished data), and further developments on such thresholds are left as a direction for future work.

Also, although we considered in this analysis foods already consumed in each country, we were unable to account for availability of these foods subnationally within a country and seasonally throughout the year (although seasonality of food prices is explored in Figure S26 in the Supporting Information online). Food purchases are generally made for an entire family, not individual members; thus, the child-specific portion sizes used may be unrealistic. Furthermore, foods in their purchasable forms may vary from foods in the portion analysis in terms of their refuse, cooking yield, and minimum purchase size. There are inherent weaknesses in the collection of household food expenditure and price data; those most likely to influence this analysis include recall bias, the presence of bulk purchases for which consumption may be spread outside of the survey recall period, reliance on predetermined lists of food to query households in some surveys, and differences between countries’ survey methodologies (see the Supporting Information online).^50^ The by-nutrient analysis focused mostly on nutrients for which there was at least moderate-certainty evidence of at least a moderate burden gap in consumption. Notably, although there are potential gaps in zinc, calcium, vitamin B_12_, and folate intake in many of the focus countries, these gaps are generally based on low certainty of evidence. Finally, there are other considerations beyond affordability and nutrient content that influence household food consumption decisions, particularly for young children. These include consistent availability, nutrition knowledge, desirability, and habits.^51–53^ For example, numerous religious fasting periods commonly observed in Ethiopia prohibit the consumption of animal-source foods for adults and older children, so messaging about the importance of continuity in young children’s consumption of affordable animal-source foods, such as dairy, even during fasting periods, is important. For both affordable and unaffordable foods, there may be additional barriers to consumption.

This study has several implications for nutrition policy and programing. Programs to increase intake of animal-source protein, iron, calcium, zinc, and folate could improve affordability by focusing on the most affordable foods (eg, through market interventions to further reduce prices), most affected households (eg, through cash transfers and other safety-net programs), or both (eg, through agricultural interventions to promote increased production among low-resource households). To increase their impact on child nutrition, programs should aim to promote those foods that are already more affordable sources of key nutrients (eg, dark-green leafy vegetables, fish, legumes, dairy products). For less affordable foods that are high in nutrients of concern (eg, chicken, beef), efforts are needed to understand how to improve their affordability, which may be through subsidies, trade policies, and/or improved production efficiencies. Given that no foods were found that could meet zinc needs at affordable prices in Ethiopia and Zambia, making zinc-rich foods more affordable or providing new sources of dietary zinc (eg, through fortification or biofortification) should be a policy priority. Affordability barriers are highest for poor and rural households, and these are driven primarily by lower food expenditures among these households. This finding underscores the importance of broader policies that can increase the food budgets available to low-income households, such as cash transfers and subsidies. Safety nets could be particularly important in Zambia, where poorer households spent relatively much less on food than in other countries and were thus more constrained by affordability.

Our findings also open several avenues for future research. For foods found to be more affordable sources of key nutrients but potentially underconsumed (eg, dark-green leafy vegetables, orange-fleshed vegetables, liver, legumes), additional research can examine what nonaffordability barriers prevent their consumption, and interventions can be designed to address these. In particular, although several of these foods are already consumed by many households, additional exploration of liver may be warranted. Chicken liver and beef liver are among the more affordable sources of vitamins A and B_12_ and all 6 micronutrients combined, but there were limited data on their consumption and prices, and we assumed prices were similar to other cuts of chicken and beef, respectively. As such, liver could be a more affordable source of other nutrients (eg, iron) than this analysis concludes, because price data from countries with available data (ie, Ethiopia, South Africa, and Mozambique) indicate liver tends to be cheaper than other cuts of meat. Or, liver may not be an acceptable food to families or young children and could thus have substantial noneconomic barriers to consumption. Small dried fish could be another important topic for future research, because it is a good source of several nutrients and is among the most affordable options when its combined nutrient composition is considered, but prices and availability appear to be highly variable across and within countries. In addition, to help develop clear affordability thresholds, future work can complement analyses such as this one with data and insights on willingness and ability to reallocate food expenditures.

## CONCLUSION

Many programs aimed at improving infant and young-child feeding practices focus on increasing knowledge and/or desire for nutritious complementary foods. However, using data from 6 countries in eastern and southern Africa, we demonstrate that affordability is also a key obstacle to improving nutrition among young children. Apart from increasing home production or wild harvest, affordability can only be addressed by interventions that reduce food prices or increase incomes—avenues not commonly pursued by existing nutrition interventions. Although affordability may not be the only cause of poor nutrient intake, interventions that increase availability of, knowledge of, or desire for nutritious foods will have limited impact if affordability is not addressed. This study offers important insights on which foods and nutrients are the most and least affordable and which households and countries are most affected by affordability barriers. These findings can be used to drive research and design evidence-based programs that address nutrient gaps and allow millions of children to reach their full potential.

## Supplementary Material

nuaa137_Supplementary_Data

## Supporting Information

The following Supporting Information is available through the online version of this article at the publisher’s website:

*Table S1* **Data sources used for each country**

*Table S2* **Nutrient requirements from complementary feeding for children aged 6–23 months**

*Table S3* **Variation in nutrient densities**

*Table S4* **Variation in food prices**

*Table S5* **Country-specific values for refuse, cooking yield, and proportion of purchasable portion**

*Table S6* **Nutrient densities used in portion-size calculations**

*Table S7* **Weekly purchasable portion sizes used in affordability analysis**

*Table S8* **Nutrients and foods that are affordable and unaffordable under alternative thresholds**

*Table S9* **Relationships between food insecurity and total household food expenditure per adult equivalent in South Africa and Tanzania**

*Table S10* **Published vitamin A supplementation cost estimates in sub-Saharan Africa**

*Table S11* **Vitamin A supplementation costs and affordability**

*Figure S1* **Share of micronutrient requirements across portion sizes (plant-source foods)**

*Figure S2* **Share of micronutrient requirements across portion sizes (animal-source foods)**

*Figure S3* **Average share of micronutrient requirements across portion sizes, by food**

*Figure S4* **Total household food and nonfood expenditures, by rural or urban setting**

*Figure S5* **Total household food and nonfood expenditures, by quintile**

*Figure S6* **Household consumption of key food groups, by rural or urban setting**

*Figure S7* **Weekly household expenditures by food group and rural or urban setting**

*Figure S8* **Household consumption of key food groups, by quintile**

*Figure S9* **Proportional weekly household expenditure by food group and quintile**

*Figure S10* **Total weekly household expenditure by food group and quintile**

*Figure S11* **Current consumption of selected nutritious foods, by rural or urban setting**

*Figure S12* **Current expenditure on selected nutritious foods, by rural or urban setting**

*Figure S13* **Current consumption of selected nutritious foods, by quintile**

*Figure S14* **Current expenditure on selected nutritious foods, by quintile**

*Figure S15* **Portion size cost, as a share of total household food expenditure per adult equivalent, by food**

*Figure S16* **Portion size cost, as a share of total household food expenditure per adult equivalent, by rural/urban setting**

*Figure S17* **Food cost per kcal by rural/urban setting**

*Figure S18* **Average share of micronutrient requirements affordability analysis, by rural/urban setting**

*Figure S19* **Portion size cost, as a share of total household food expenditure per adult equivalent, by quintile**

*Figure S20* **Food cost per kcal by quintile**

*Figure S21* **Average share of micronutrient requirements affordability analysis, by quintile**

*Figure S22* **Food prices per kg by country, adjusted for currency exchange rates and purchasing power parities**

*Figure S23* **Portion size cost net current expenditure per AEQ, as a share of total household food expenditure per AEQ**

*Figure S24* **Sensitivity analysis results for nutrient density and refuse for dark leafy green vegetables, legumes, fish, chicken, and chicken liver**

*Figure S25* **Cost per kcal sensitivity analysis results for nutrient density and refuse for legumes, fish, and chicken**

*Figure S26A* **Seasonal price variation by food and country—Ethiopia (Birr)**

*Figure S26B* **Seasonal price variation by food and country—Mozambique (Metical)**

*Figure S26C* **Seasonal price variation by food and country—South Africa (Rand)**

*Figure S26D* **Seasonal price variation by food and country—Tanzania (Shillings)**

*Figure S26E* **Seasonal price variation by food and country—Uganda (Shillings)**
