# Supplementary material for: Affordability of nutritious foods for complementary feeding in Eastern and Southern Africa
Source: Nutr Rev. 2021 Mar 8;79(Suppl 1):35–51. doi: 10.1093/nutrit/nuaa137 (PMC7948081; doi:10.1093/nutrit/nuaa137)
Supplement: nuaa137_Supplementary_Data [file nuaa137_supplementary_data.docx]

**SUPPORTING INFORMATION**

Contents

[Supplementary Text – Food and non-food expenditure definitions 3](#_Toc65486969)

[Supplementary Text – Methodological details for each country 3](#_Toc65486970)

[Supplementary Text – Methodological details for certain foods 5](#_Toc65486971)

[Table S1: Data sources used for each country 7](#_Toc65486972)

[Table S2: Nutrient requirements from complementary feeding for children aged 6-23 months 9](#_Toc65486973)

[Table S3: Variation in nutrient densities 10](#_Toc65486974)

[Table S4: Variation in food prices 12](#_Toc65486975)

[Table S5: Country-specific values for refuse, cooking yield, and proportion of purchasable portion 13](#_Toc65486976)

[Table S6: Nutrient densities used in portion size calculations 15](#_Toc65486977)

[Table S7: Weekly purchasable portion sizes used in affordability analysis 16](#_Toc65486978)

[Supplementary text – average share of micronutrient requirements analysis details 17](#_Toc65486979)

[Table S8: Nutrients and foods which are affordable and unaffordable under alternative thresholds 18](#_Toc65486980)

[Table S9: Relationships between food insecurity and total household food expenditure per adult equivalent in South Africa and Tanzania 20](#_Toc65486981)

[Supplementary Text – Vitamin A supplementation affordability comparison 22](#_Toc65486982)

[Table S10: Published vitamin A supplementation cost estimates in Sub-Saharan Africa 22](#_Toc65486983)

[Table S11: Vitamin A supplementation costs and affordability 24](#_Toc65486984)

[Figure S1: Share of micronutrient requirements across portion sizes (plant-source foods) 25](#_Toc65486985)

[Figure S2: Share of micronutrient requirements across portion sizes (animal-source foods) 26](#_Toc65486986)

[Figure S3: Average share of micronutrient requirements across portion sizes, by food 27](#_Toc65486987)

[Figure S4: Total household food and non-food expenditure, by rural/urban setting 28](#_Toc65486988)

[Figure S5: Total household food and non-food expenditure, by quintile 29](#_Toc65486989)

[Figure S6: Household consumption of key food groups, by rural/urban setting 30](#_Toc65486990)

[Figure S7: Weekly household expenditure by food group and rural/urban setting 31](#_Toc65486991)

[Figure S8: Household consumption of key food groups, by quintile 32](#_Toc65486992)

[Figure S9: Proportional weekly household expenditure by food group and quintile 34](#_Toc65486993)

[Figure S10: Total weekly household expenditure by food group and quintile 35](#_Toc65486994)

[Figure S11: Current consumption of selected nutritious foods, by rural/urban setting 36](#_Toc65486995)

[Figure S12: Current expenditure on selected nutritious foods, by rural/urban setting 37](#_Toc65486996)

[Figure S13: Current consumption of selected nutritious foods, by quintile 39](#_Toc65486997)

[Figure S14: Current expenditure on selected nutritious foods, by quintile 41](#_Toc65486998)

[Figure S15: Portion size cost, as a share of total household food expenditure per adult equivalent, by food 43](#_Toc65486999)

[Figure S16: Portion size cost, as a share of total household food expenditure per adult equivalent, by rural/urban setting 45](#_Toc65487000)

[Figure S17: Food cost per kcal by rural/urban setting 49](#_Toc65487001)

[Figure S18: Average share of micronutrient requirements affordability analysis, by rural/urban setting 50](#_Toc65487002)

[Figure S19: Portion size cost, as a share of total household food expenditure per adult equivalent, by quintile 51](#_Toc65487003)

[Figure S20: Food cost per kcal by quintile 58](#_Toc65487004)

[Figure S21: Average share of micronutrient requirements affordability analysis, by quintile 60](#_Toc65487005)

[Figure S22: Food prices per kg by country, adjusted for currency exchange rates and purchasing power parities 62](#_Toc65487006)

[Figure S23: Portion size cost net current expenditure per AEQ, as a share of total household food expenditure per AEQ 63](#_Toc65487007)

[Figure S24: Sensitivity analysis results for nutrient density and refuse for dark leafy green vegetables, legumes, fish, chicken, and chicken liver 65](#_Toc65487008)

[Figure S25: Cost per kcal sensitivity analysis results for nutrient density and refuse for legumes, fish, and chicken 70](#_Toc65487009)

[Figure 26A: Seasonal price variation by food and country – Ethiopia (Birr) 71](#_Toc65487010)

[Figure 26B: Seasonal price variation by food and country – Mozambique (Metical) 72](#_Toc65487011)

[Figure 26C: Seasonal price variation by food and country – South Africa (Rand) 73](#_Toc65487012)

[Figure 26D: Seasonal price variation by food and country – Tanzania (Shillings) 75](#_Toc65487013)

[Figure 26E: Seasonal price variation by food and country – Uganda (Shillings) 76](#_Toc65487014)

[References 78](#_Toc65487015)

# Supplementary Text – Food and non-food expenditure definitions

Food expenditures (aggregate and for specific food items) are defined as the value of consumption for all foods and beverages (non-alcoholic and alcoholic) consumed by the household, both in the home and away from the home. This includes purchases, value of own production, and value of foods consumed from gifts and other in-kind sources.

Non-food expenditure includes expenditures on non-food items (including narcotics), services (including food services charges such as milling or butchering), healthcare, education (including some in-kind scholarships), housing (rent and imputed rent for households that own their property), insurance payments, taxes, and fees. Non-routine bulk expenditures (such as for weddings) are included. House purchases, the value of durable goods owned by the household, and purchases for household enterprises were excluded.

Because data were collected over a relatively short period of time for each country, expenditures have not been adjusted for regional price differences or inflation, except when converted to common units (e.g., US dollars).

# Supplementary Text – Methodological details for each country

*Ethiopia*

Data on age in months was not available in Ethiopia's Household Consumption and Expenditure Survey (HCE), so the analysis was conducted for all households with children under two years. The HCE elicited information on food consumption and expenditures by asking households to log food consumption in a diary over the course of a week. Information from the diaries was then collated on-site by a survey enumerator. "Food Expenditure" includes expenditures from purchases and the estimated value of foods consumed from own production, gifts, in-kind, and other sources. Value of food from own production, gifts, and other sources was estimated in the survey based on household's estimation of value and based on market prices collected as part of the HCE. For this analysis, we estimate prices for households that did not consume each food item based on the prices available in the HCE for households in nearby geographical locations, starting with Enumeration Area (EA). When prices were not available for the same EA and food, they were estimated at higher geographic levels.

*Mozambique*

In Mozambique, most households were surveyed three different times. Because household composition may change over the course of a year, we treated each household-trimester observation as a separate observation in our analysis. Data on age in months was not available in Mozambique’s Inquérito sobre Orcamento Familiar (Household Budget Survey/IOF), so the analysis was conducted for all households with children under two years. The IOF collected data on household food consumption and expenditures by asking households, over three different visits in one week, to recall quantities consumed and expenditures for a long list of foods purchased over the past week. Households were also asked about quantities consumed from own production for a subset of these foods. Survey encoders used survey price data to estimate the value of foods consumed from own production. Market price data were collected via a separate survey module (the community questionnaire). We matched households to prices based on location (primary sampling unit) and trimester. When prices were not available for the same PSU, food, and trimester, we estimated them based on the closest price data available.

*South Africa*

As with several of the other surveys, we conducted the affordability analysis for the subset of all households with children under two, since data on household members’ age in months was not available. South Africa’s Living Conditions Survey (LCS) elicited information on household food consumption and expenditure by asking households to keep a diary over a two-week period. If foods were consumed from own production, gifts, in-kind wages, own shop stocks, or other sources besides purchases, households were asked to estimate the value of these foods. These diaries were then collated by survey enumerators and categorized into detailed food codes. Although collected in the LCS, data on the source of food consumption (whether it came from purchases, own production, or other sources) was not available in the survey data files. Food price data was not collected in the LCS. Instead, we used data from Statistics South Africa (market prices for urban areas, at the province level) and from reports published by the National Agricultural Marketing Council (NAMC; market prices for rural areas, at the national level).^1,2^ The NAMC publishes food price monitoring reports based on non-public data obtained from Statistics South Africa. Because rural price data was also only available for a subset of foods, we estimated rural prices based on urban province-level data and the rural-urban price difference for similar items. For example, the price for spinach in rural areas was estimated based on the price for spinach in urban areas and the difference between the urban and rural price for cabbage. If urban data was missing for a particular food and province, estimates were generated similarly. Price data was matched to the month a household was surveyed when possible; when this was not possible, prices were estimated from surrounding months or years using food inflation rates.

*Tanzania*

As with several of the other surveys, we conducted the affordability analysis for the subset of all households with children under two, since data on household members’ age in months was not available. Tanzania’s Living Standards Measurement Survey (LSMS) collected data on household food consumption and expenditures using the recall method; households were asked by survey enumerators to recall quantities consumed and expenditures made over the past week for a long list of foods. Market prices were collected as part of the LSMS for a different (but overlapping) set of foods. Value of food from own production, gifts, and other sources (besides purchases) was estimated based on these market prices. When price data was not available for the same item or location, we matched households to prices based on nearby locations or (to value non-purchases only) we used implicit prices paid for the same item by other households in the same setting (urban/rural) and district.

*Uganda*

We used data collected on household members’ birthdates and survey dates to identify the subset of households with children aged 6-23 months. Uganda’s Living Standards Measurement Survey (LSMS) elicited information on food consumption and expenditures by asking households to recall quantities of a long list of foods consumed in the past week. For purchased items, households were asked about expenditures and market prices. For items from own production or in-kind sources, households were asked about the value of the items and farm gates prices were recorded. We used market prices to conduct the affordability analysis. Sometimes prices were available for multiple units of an item (e.g. kilograms, pieces, bundles, etc.), in which case we chose the most standard and/or most frequently reported units and did not convert across units unless necessary. When market prices for a particular food and unit were not recorded for a particular household (because the household did not consume that item or reported a price in another unit), we estimated price based on data from nearby households, or from the closest households geographically.

*Zambia*

The Zambia Living Conditions Monitoring Survey (LCMS) collected data on household members’ age in months, which we used to identify households with children aged 6-23 months. The LCMS elicited information on food value by asking households to recall quantities of a long list of foods purchased, consumed from own production, and received without payment in the past four weeks (for cereals, salt, cooking oil, and spices) or the past two weeks (for all other foods). The value of foods consumed from own production or received without payment was estimated by the households (households were asked how much the item would cost if they were to buy it). The LCMS only collected data on value of foods consumed, not quantities. Data on food prices was taken from a series of monthly reports on inflation released by the Zambia Central Statistical Office. Each report contains data on national average retail prices for a selection of both food and non-food products. Information by sub-geography (e.g. province or rural/urban) was not available. When available, we matched price data to households based on the month the household was surveyed (April-June 2015). Because not all food items were included in the reports each month, we estimated food prices from neighboring months (e.g. March or July) when needed. The only food item without data in a neighboring month was milk; the price of milk was estimated based on the monthly price of super milk and the price ratio between milk and super milk from a later month.

# Supplementary Text – Methodological details for certain foods

For most foods, we exactly matched foods in the price data to foods in US Department of Agriculture (USDA) and local nutrient composition tables to estimate nutrient density, cooking yield, and refuse. To estimate current consumption and expenditure on these foods, if multiple similar varieties were covered in the survey data, we summed consumption/expenditure over these varieties for each household. For some foods, additional assumptions had to be made. These are detailed below:

*Dark Green Leafy Vegetables (DGLV)*

For most countries, surveys captured several different varieties of DGLV. Because nutrient composition can vary substantially across DGLV, we used an average covering multiple varieties of DGLV in multiple food composition tables (Table S3), and varied nutrient densities in sensitivity analysis (Figures S24-S25). To estimate prices, we averaged prices across all DGLV varieties that were consumed by 10% or more of surveyed households (Table S4).

*Legumes*

Legumes were treated similarly to DGLV. For nutrient density, we used an average across multiple varieties (Table S3) and varied this in sensitivity analysis (Figures S24-25). We calculated prices as the average across all legumes consumed by 10% of household or more (Table S4).

*Fish*

We analyzed three categories of fish – small dried fish, fresh/frozen fish, and small tinned fish – chosen based on consumption data. For Ethiopia, fish was omitted entirely because it was consumed by less than 1% of households. For fresh/frozen fish, we used the nutrient composition for an average of tilapia and mackerel for all nutrients except vitamin A, where we used the nutrient composition for mackerel only (because tilapia does not contain vitamin A). For small dried fish and small tinned fish, we used an average nutrient composition from multiple sources (Table S3), similar to how we estimated the nutrient content for DGLV and legumes. For the most part, only one set of prices was available for each category of fish (Table S4).

*Chicken*

Data on chicken nutrient content came from USDA food composition tables. In most countries, chickens are typically purchased whole, and could be purchased alive or dead. For live chickens, we added an additional 25% refuse to the US Department of Agriculture refuse data based on Azahan.^3^ When price data did not specify whether the price corresponded to purchasing a live or dead chicken, we used the average refuse and varied refuse in sensitivity analysis (Figures S24-25).

*Chicken Liver*

Unless chicken liver or chicken offal price data were available (this was only the case for Mozambique), we used the price and refuse for chicken, assuming that households could obtain chicken liver from purchasing a whole chicken and that chicken liver is not sold separately. We also ran a sensitivity analysis with zero refuse, which applies to the scenario in which households can purchase chicken liver separately (Figures S24-25).

*Beef Liver*

Beef liver or beef offal prices were available for Ethiopia, Mozambique, and South Africa. For Tanzania, Uganda, and Zambia, we used the price for beef and zero refuse (assuming that households can purchase beef liver separately for around the same price as other cuts of beef).

# Table S1: Data sources used for each country

| **Country** | **Consumption and Expenditure Data Source** | **Reference Period** | **Survey Period** | **Sub-National Region at which the Survey is Representative** | **Data Collection Method** | **Food Price Data Source** | **Notes** |
| --- | --- | --- | --- | --- | --- | --- | --- |
| Ethiopia | 2015-16 Household Consumption and Expenditure Survey (HCE)^4^ | 1 week | September 2015-August 2016 | National, Region-Urban/Rural combination | Diary | 2015-16 HCE^4^ |  |
| Mozambique | 2014-15 15 Inquérito sobre Orcamento Familiar (Household Budget Survey/IOF)^5^ | 1 week | August 2014-August 2015 | National, Provincial, Urban/Rural | Recall | 2014-15 IOF ^5^ | Foods consumed by in-kind sources (e.g., gifts) were not covered by the survey. Most households were interviewed 3 times over the course of 1 year; these are treated as separate observations |
| South Africa | 2014-15 Living Conditions Survey^6^ | 2 weeks | October 2014-October 2015 | National, Provincial | Diary | Statistics South Africa and National Agriculture Marketing Council price reports^1,2^ | Breakdowns of food expenditure by purchases vs. own production/other sources were not available |
| Tanzania | 2014-15 Living Standards Measurement Survey (LSMS)^7^ | 1 week | October 2014-September 2015 | National, Sub-National Domain (Dar es Salaam, Other Mainland Urban Areas, Rural Mainland, Zanzibar) | Recall | 2014-15 LSMS^7^ |  |
| Uganda | 2013-14 Living Standards Measurement Survey^8^ | 1 week | September 2013-October 2014 | National, Region-Urban/Rural combination (Kampala City, Other Urban Areas, Central Rural, Eastern Rural, Western Rural, Northern Rural) | Recall | 2013-14 LSMS ^8^ |  |
| Zambia | 2015 Living Conditions Monitoring Survey^9^ | 2 weeks | April-June 2015 | National, Provincial, Urban/Rural | Recall | Zambia Central Statistical Office monthly price reports^10^ | Sub-national price data were not available |

# Table S2: Nutrient requirements from complementary feeding for children aged 6-23 months

| **Nutrient** | **Requirement type** | **Animal or plant source*** | **Nutrient Requirements (by age)** | | | **Proportion of nutrient requirements needed from complementary feeding**** |
| --- | --- | --- | --- | --- | --- | --- |
|  |  |  | **6-11 m** | **12-35 m** | **6-23 m (estimated)** |  |
| Protein (g) | RDA | Animal source foods | 11.0 | 13.0 | 12.3 | 0.49 |
| Iron (mg) | RNI | Animal source foods | 6.2 | 3.9 | 4.7 | 0.98 |
| Iron (mg) | RNI | Plant source foods | 9.3 | 5.8 | 7.0 | 0.98 |
| Zinc (mg) | RNI | Animal source foods | 2.5 | 2.4 | 2.4 | 0.87 |
| Zinc (mg) | RNI | Plant source foods | 4.1 | 4.1 | 4.1 | 0.87 |
| Calcium (mg) | RNI | Both | 400.0 | 500.0 | 466.7 | 0.65 |
| Vitamin A (mcg RAE) | RDA | Both | 500.0 | 300.0 | 366.7 | 0.17 |
| Vitamin B12 (mcg) | RDA | Both | 0.5 | 0.9 | 0.8 | 0.70 |
| Folate (mcg DFE) | RDA | Both | 80.0 | 150.0 | 126.7 | 0.60 |

Notes: RDA = Recommended Dietary Allowance; RNI = Reference Nutrient Intake; RAE = Retinol Activity Equivalents; mcg = micrograms; DFE = Dietary Folate Equivalents

*For iron, we assumed 15% dietary iron bioavailability for animal-source foods and 10% for plant foods; for zinc we assumed 50% dietary zinc bioavailability for animal-source foods and 30% for legumes, nuts, and seeds ^11^. **Based on ^12^.

# Table S3: Variation in nutrient densities

| **Dark Green Leafy Vegetables** | | | | | |
| --- | --- | --- | --- | --- | --- |
| **Food** | **Food Composition Table (Source)** | **Nutrient Content** | | | |
|  |  | **Iron (mg)** | **Vitamin A (mcg RAE)** | **Calcium (mg)** | **Folate (mcg DFE)** |
| Amaranth Leaves | Mozambique ^13^ | 3.8 | 392 | 407 | 25 |
| Cowpea Leaves |  | 2.3 | 242 | 97 | 18 |
| Pumpkin Leaves |  | 1.2 | 269 | 67 | 26 |
| Sweet Potato Leaves |  | 2.4 | 233 | 98 | 17 |
| Cassava Leaves | Zambia ^14^ | 3.7 | - | - | - |
| Cowpea Leaves |  | 5.7 | - | - | - |
| Pumpkin Leaves |  | 5.6 | - | - | - |
| Rape (Brassica Carinata) |  | 5.5 | - | - | - |
| Amaranth Leaves | West Africa ^15^ | 4.9 | 228 | - | 42 |
| Cassava Leaves |  | 4.4 | 271 | - | 62 |
| Cowpea Leaves |  | 4.0 | 142 | - | 68 |
| Pumpkin Leaves |  | 1.8 | - | - | 19 |
| Spinach |  | 2.5 | 387 | - | 93 |
| Sweet Potato Leaves |  | 2.8 | 463 | - | 42 |
| Amaranth Leaves | USDA ^16^ | 2.3 | 139 | 209 | 57 |
| Mustard Greens |  | 3.2 | 80 | 43 | 25 |
| Pumpkin Leaves |  | 0.6 | 147 | 33 | 49 |
| Sweet Potato Leaves |  | 3.6 | 524 | 136 | 146 |
| Spinach |  | 0.9 | 527 | 118 | 9 |
| Median [10^th^ and 90^th^ percentiles] | | 3.2 [1.1-5.5] | 256 [140-506] | 98 [41-249] | 42 [17-83] |

| **Legumes** | | | | | |
| --- | --- | --- | --- | --- | --- |
| **Food** | **Food Composition Table (Source)** | **Nutrient Content** | | | |
|  |  | **Iron (mg)** | **Zinc (mg)** | **Folate (mcg DFE)** | **Energy (kcal)** |
| Cowpeas | Mozambique^13^ | 1.7 | 1.1 | 50 | 105 |
| Cowpeas | West Africa ^15^ | 2.3 | 1.5 | 77 | 117 |
| Lentils |  | 2.2 | 1.3 | 54 | 109 |
| Pigeon Peas |  | 1.5 | 0.7 | 84 | 111 |
| Cowpeas | USDA ^16^ | 3.1 | 1.9 | 142 | 117 |
| Kidney Beans |  | 2.9 | 1.1 | 130 | 127 |
| Lentils |  | 3.3 | 1.3 | 181 | 114 |
| Pigeon Peas |  | 1.1 | 0.9 | 111 | 121 |
| Lima Beans |  | 2.4 | 1.0 | 83 | 115 |
| Split Peas |  | 1.3 | 1.0 | 65 | 116 |
| Median [10^th^ and 90^th^ percentiles] | | 2.3 [1.3-3.1] | 1.1 [0.9-1.5] | 84 [54-146] | 116 [109-122] |

| **Small Dried Fish** | | | | | | | | |
| --- | --- | --- | --- | --- | --- | --- | --- | --- |
| **Food** | **Location and Source** | **Nutrient Content** | | | | | | |
|  |  | **Iron (mg)** | **Zinc (mg)** | **Calcium (mg)** | **Vitamin B_12_ (mcg)** | **Vitamin A (mcg RAE)** | **Protein (g)** | **Energy (kcal)** |
| Small Dried Fish | Mozambique GAIN/CGIAR Analysis (unpublished) | 36.8 | 11.1 | 960 | - | - | 56.7 | - |
| Small Dried Fish | Tanzania FCT ^17^ | 2.5 | 5.2 | 1700 | 12.0 | - | 58.6 | 335 |
| Dried Mukene | Lake Victoria region, Kabahenda et al. ^18^ | 10.7 | 10.3 | 1556 | - | - | 58.8 | - |
| Dried Kapenta | Zambia (Steiner-Asiedu et al via Longley et al ^19^) | 10.0 | 10.0 | 2360 | - | 186 | 63.3 | - |
| Median [10^th^-90^th^ percentiles] | | 10.4 [5.5-35.2] | 10.0 [5.7-10.8] | 2030 [1258-3218] | 12 | 363 [221-505] | 59 [56-63] | 255 [218-319] |

| **Small Tinned Fish** | | | | | | | |
| --- | --- | --- | --- | --- | --- | --- | --- |
| **Food** | **Food Composition Table (Source)** | **Nutrient Content** | | | | | |
|  |  | **Iron (mg)** | **Zinc (mg)** | **Calcium (mg)** | **Vitamin A (mcg RAE)** | **Protein (g)** | **Energy (kcal)** |
| Tinned Sardines | Zambia ^14^ | 3 | - | 400 | 60 | 20 | 309 |
| Canned Sardines | USDA ^16^ | 2.9 | 1.3 | 382 | 32 | 24.6 | 208 |
| Canned Anchovy |  | 4.6 | 2.4 | 232 | 12 | 28.9 | 210 |
| Median [10^th^-90^th^ percentiles] | | 3.0 [2.9-4.3] | 1.9 [1.4-2.3] | 382 [262-396] | 32 [16-54] | 24.6 [21-28] | 210 [208-289] |

Notes: RAE = Retinol Activity Equivalents; mcg = micrograms; DFE = Dietary Folate Equivalents; FCT = Food Composition Table; USDA = US Department of Agriculture

# Table S4: Variation in food prices

| **Dark Green Leafy Vegetables** | | | |
| --- | --- | --- | --- |
| **Country** | **Food (as listed in data)** | **Price per kg, local currency [95% CI]** | **Price per kg, 2018 USD [95% CI]** |
| Ethiopia | Ethiopian Kale | 6.7 [6.4-6.9] | 0.33 [0.32-0.34] |
| Mozambique | Cassava Leaves | 22.6 [21.7-23.5] | 0.56 [0.54-0.58] |
|  | Cowpea Leaves | 26.2 [25.3-27.0] | 0.65 [0.62-0.67] |
|  | Pumpkin Leaves | 25.4 [24.5-26.2] | 0.63 [0.61-0.65] |
|  | Sweet Potato Leaves | 24.1 [23.1-25.0] | 0.60 [0.57-0.62] |
| South Africa | Fresh Spinach/Morogo | 36.3 [36.0-36.6] | 3.21 [3.18-3.24] |
| Tanzania | Spinach | 1066 [990-1141] | 0.55 [0.51-0.59] |
| Uganda | Amaranth Greens | 1335 [1196-1473] | 0.43 [0.39-0.48] |
| Zambia | Pumpkin Leaves | 4.7 [4.6-4.7] | 0.59 [0.59-0.59] |
|  | Rape Greens | 4.6 [4.6-4.6] | 0.61 [0.61-0.61] |
|  | Sweet Potato Leaves | 5.3 [5.3-5.3] | 0.70 [0.70-0.70] |
| **Legumes** | | | |
| Ethiopia | Split Field Peas | 30.7 [30.1-31.3] | 1.50 [1.47-1.53] |
|  | Split Lentils | 50.9 [50.3-51.6] | 2.49 [2.46-2.53] |
| Mozambique | Cowpeas | 27.0 [26.4-27.7] | 0.67 [0.65-0.68] |
|  | Butter Beans | 51.7 [50.0-51.4] | 1.25 [1.24-1.27] |
|  | Pigeon Peas | 23.2 [22.6-23.9] | 0.57 [0.56-0.59] |
| Tanzania | Beans | 1798 [1737-1859] | 0.58 [0.56-0.60] |
|  | Peas | 1473 [1364-1583] | 0.48 [0.44-0.51] |
| South Africa | Dried Beans | 29.8 [29.8-29.9] | 2.64 [2.64-2.65] |
| Uganda | Dried Beans | 1976 [1907-2045] | 0.64 [0.62-0.66] |
| Zambia | Dried Beans | 13.4 [13.4-13.4] | 1.77 [1.77-1.77] |
| **Fish** | | | |
| Mozambique | Dried Fish | 132.5 [129.4-135.7] | 3.28 [3.20-3.35] |
|  | Fresh, Frozen, or Chilled Fish | 75.6 [73.3-78.0] | 1.87 [1.81-1.93] |
|  | Fresh, Frozen, or Chilled Carapau | 87.8 [87.1-88.5] | 2.17 [2.16-2.19] |
| South Africa | Tinned Fish (excl. tuna) | 38.0 [37.9-38.1] | 3.36 [3.35-3.37] |
| Tanzania | Dagaa | 5194 [4903-5485] | 2.69 [2.54-2.84] |
|  | Fresh Fish | 3936 [3757-4116] | 2.04 [1.95-2.13] |
| Uganda | Dry/Smoked Fish | 14046 [13543-14548] | 4.54 [4.38-4.70] |
|  | Fresh Fish | 6847 [6550-7143] | 2.21 [2.12-2.31] |
| Zambia | Dried Kapenta | 92.2 [91.9-92.5] | 12.19 [12.15-12.23] |
|  | Frozen Fish | 23.1 [23.1-23.1] | 3.05 [3.05-3.05] |

Note: Prices per kg in US Dollars were estimated using local currency inflation and 2018 exchange rates from the International Monetary Fund ^20,21^. If price data were available for multiple varieties of a food, we included only those specific foods for which consumption exceeded 10% of households. For Zambia, we were only able to incorporate variation across the three months covered by the survey, not geographical variation.

# Table S5: Country-specific values for refuse, cooking yield, and proportion of purchasable portion

| **Country** | **Food** | **Refuse from purchasable portion*** | **Cooking yield**** | **Proportion of purchasable portion** |
| --- | --- | --- | --- | --- |
| Ethiopia | Beef | 0.15 | 0.70 | 0.60 |
|  | Beef Liver | 0.00 | 0.73 | 0.73 |
|  | Carrots | 0.11 | 0.94 | 0.84 |
|  | Chicken | 0.42 | 0.77 | 0.45 |
|  | Chicken Liver | 0.42 | 0.62 | 0.36 |
|  | Cottage Cheese | 0.00 | 1.00 | 1.00 |
|  | Dark Green Leafy Vegetables | 0.10 | 0.98 | 0.88 |
|  | Eggs | 0.12 | 1.01 | 0.89 |
|  | Fresh Milk | 0.00 | 1.00 | 1.00 |
|  | Groundnuts | 0.10 | 1.00 | 0.90 |
|  | Legumes | 0.00 | 2.50 | 2.50 |
|  | Pumpkin | 0.30 | 0.92 | 0.64 |
| Mozambique | Beef | 0.15 | 0.70 | 0.60 |
|  | Beef Liver | 0.00 | 0.73 | 0.73 |
|  | Chicken | 0.34 | 0.77 | 0.51 |
|  | Chicken Liver | 0.00 | 0.62 | 0.62 |
|  | Dark Green Leafy Vegetables | 0.10 | 0.98 | 0.88 |
|  | Eggs | 0.12 | 1.01 | 0.89 |
|  | Fresh/Frozen Fish | 0.00 | 0.85 | 0.85 |
|  | Fresh Milk | 0.00 | 1.00 | 1.00 |
|  | Groundnuts | 0.10 | 1.00 | 0.90 |
|  | Legumes | 0.00 | 2.50 | 2.50 |
|  | Mango | 0.29 | 1.00 | 0.71 |
|  | Small Dried Fish | 0.00 | 1.00 | 1.00 |
|  | Sweet Potato | 0.28 | 0.95 | 0.68 |
| South Africa | Beef | 0.15 | 0.70 | 0.60 |
|  | Beef Liver | 0.00 | 0.73 | 0.73 |
|  | Carrots | 0.11 | 0.94 | 0.84 |
|  | Chicken | 0.34 | 0.77 | 0.51 |
|  | Chicken Liver | 0.00 | 0.62 | 0.62 |
|  | Dark Green Leafy Vegetables | 0.10 | 0.98 | 0.88 |
|  | Eggs | 0.12 | 1.01 | 0.89 |
|  | Fresh Milk | 0.00 | 1.00 | 1.00 |
|  | Legumes | 0.00 | 2.50 | 2.50 |
|  | Mango | 0.29 | 1.00 | 0.71 |
|  | Peanut Butter | 0.00 | 1.00 | 1.00 |
|  | Small Tinned Fish | 0.00 | 1.00 | 1.00 |
|  | Sour Milk | 0.00 | 1.00 | 1.00 |
| Tanzania | Beef | 0.15 | 0.70 | 0.60 |
|  | Beef Liver | 0 | 0.73 | 0.73 |
|  | Carrots | 0.11 | 0.94 | 0.84 |
|  | Chicken | 0.49 | 0.77 | 0.39 |
|  | Chicken Liver | 0.49 | 0.62 | 0.32 |
|  | Dark Green Leafy Vegetables | 0.10 | 0.98 | 0.88 |
|  | Eggs | 0.12 | 1.01 | 0.89 |
|  | Fresh/Frozen Fish | 0.00 | 0.85 | 0.85 |
|  | Fresh Milk | 0.00 | 1.00 | 1.00 |
|  | Groundnuts | 0.10 | 1.00 | 0.90 |
|  | Legumes | 0.00 | 2.50 | 2.50 |
|  | Mango | 0.29 | 1.00 | 0.71 |
|  | Papaya | 0.38 | 1.00 | 0.62 |
|  | Small Dried Fish | 0.00 | 1.00 | 1.00 |
| Uganda | Beef | 0.15 | 0.70 | 0.60 |
|  | Beef Liver | 0.00 | 0.73 | 0.73 |
|  | Chicken | 0.42 | 0.77 | 0.45 |
|  | Chicken Liver | 0.42 | 0.62 | 0.36 |
|  | Dark Green Leafy Vegetables | 0.10 | 0.98 | 0.88 |
|  | Eggs | 0.12 | 1.01 | 0.89 |
|  | Fresh/Frozen Fish | 0.00 | 0.85 | 0.85 |
|  | Fresh Milk | 0.00 | 1.00 | 1.00 |
|  | Groundnuts | 0.00 | 1.00 | 1.00 |
|  | Legumes | 0.00 | 2.50 | 2.50 |
|  | Small Dried Fish | 0.00 | 1.00 | 1.00 |
| Zambia | Bananas | 0.36 | 1.00 | 0.64 |
|  | Beef | 0.15 | 0.70 | 0.60 |
|  | Beef Liver | 0.00 | 0.73 | 0.73 |
|  | Carrots | 0.11 | 0.94 | 0.84 |
|  | Chicken | 0.34 | 0.77 | 0.51 |
|  | Chicken Liver | 0.34 | 0.62 | 0.41 |
|  | Dark Green Leafy Vegetables | 0.10 | 0.98 | 0.88 |
|  | Eggs | 0.12 | 1.01 | 0.89 |
|  | Fresh/Frozen Fish | 0.00 | 0.85 | 0.85 |
|  | Fresh Milk | 0.00 | 1.00 | 1.00 |
|  | Groundnuts | 0.10 | 1.00 | 0.90 |
|  | Legumes | 0.00 | 2.50 | 2.50 |
|  | Okra | 0.00 | 1.00 | 1.00 |
|  | Oranges | 0.27 | 1.00 | 0.73 |
|  | Pumpkin | 0.30 | 0.92 | 0.64 |
|  | Small Dried Fish | 0.00 | 1.00 | 1.00 |
|  | Sour Milk | 0.00 | 1.00 | 1.00 |

Notes: if item is consumed raw (e.g. fresh milk) then a value of 1.00 was used for cooking yield. Variation in refuse and cooking yields for the same item across countries reflects differences in the form in which the item was purchased (e.g. some surveys recorded prices for live chickens vs. dead chickens). See supplementary methods for more details. * Refuse values are from ^16^ and ^3^** Cooking yield values are from ^22^ and ^23^

# Table S6: Nutrient densities used in portion size calculations

| **Food** | **Iron (mg)** | **Vit A (mcg RAE)** | **Calcium (mg)** | **Zinc (mg)** | **Folate (mcg DFE)** | **Vit B12 (mcg)** |
| --- | --- | --- | --- | --- | --- | --- |
| Small dried fish | 10.4 | 363 | 2030 | 10.0 | 19 | 12.0 |
| Chicken liver | 12.3 | 4,139 | 11 | 4.0 | 569 | 19.0 |
| Beef liver | 6.5 | 9,442 | 6 | 5.3 | 253 | 70.6 |
| Eggs | 1.2 | 149 | 50 | 1.1 | 44 | 1.1 |
| Beef | 2.8 | 0 | 8 | 6.5 | 8 | 2.6 |
| Dark leafy greens | 3.2 | 256 | 98 | 0.3 | 42 | 0.0 |
| Chicken | 1.2 | 46 | 14 | 1.9 | 5 | 0.3 |
| Milk | 0.0 | 46 | 113 | 0.4 | 5 | 0.5 |
| Groundnuts | 1.3 | 0 | 57 | 2.3 | 86 | 0.0 |
| Legumes | 2.3 | 0 | 25 | 1.1 | 84 | 0.0 |
| Yogurt/Sour milk | 0.1 | 27 | 121 | 0.6 | 7 | 0.4 |
| Mango | 0.5 | 152 | 13 | 0.1 | 33 | 0.0 |
| Carrot | 0.3 | 852 | 30 | 0.2 | 14 | 0.0 |
| Pumpkin | 0.6 | 288 | 15 | 0.2 | 9 | 0.0 |
| Okra | 0.3 | 14 | 77 | 0.4 | 46 | 0.0 |
| Fresh/frozen fish | 0.7 | 33 | 14 | 0.5 | 4 | 4.4 |
| Cottage cheese | 0.1 | 37 | 83 | 0.4 | 12 | 0.4 |
| Small tinned fish | 3.0 | 32 | 382 | 1.9 | 10 | 8.9 |
| Orange-fleshed sweet potatoes | 0.7 | 787 | 27 | 0.2 | 6 | 0.0 |
| Papaya | 0.3 | 47 | 20 | 0.1 | 37 | 0.0 |
| Oranges | 0.1 | 11 | 40 | 0.1 | 30 | 0.0 |
| Bananas | 0.3 | 3 | 5 | 0.2 | 20 | 0.0 |

Notes: RAE=retinol activity equivalents, mcg=micrograms, DFE=dietary folate equivalents. The nutrient densities shown come from a combination of local food composition tables (for small dried fish, dark leafy greens, and legumes) and USDA data (for other foods), as detailed in Table S3. Nutrient densities for mango are based on averages from West African ^15^ and Mozambique ^13^ food composition tables. Shading indicates that food was included (for at least one country) in the analysis for a given nutrient. All six nutrients were considered in average share of micronutrient requirements analysis for all foods with data in at least two countries.

# Table S7: Weekly purchasable portion sizes used in affordability analysis

|  | Protein | Iron | Vitamin A | Calcium | Zinc | Folate | Vitamin B_12_ |
| --- | --- | --- | --- | --- | --- | --- | --- |
| Bananas |  |  |  |  |  | 2.08 |  |
| Beef | 0.12 | 0.96 |  |  | 0.19 |  | 0.12 |
| Beef Liver |  | 0.34 | 0.003 |  | 0.19 | 0.14 | 0.004 |
| Carrots |  |  | 0.03 |  |  |  |  |
| Chicken* | 0.16-0.21 |  |  |  | 0.77-0.87 |  |  |
| Chicken Liver* |  | 0.21-0.41 | 0.01-0.02 |  | 0.45-0.51 | 0.11 | 0.02 |
| Cottage Cheese | 0.19 |  | 0.59 |  |  |  |  |
| DGLV |  | 0.85 | 0.10 | 1.23 |  | 0.72 |  |
| Eggs | 3.78 |  | 3.29 |  | 15.86 | 13.60 | 3.84 |
| Fresh Milk | 0.64 |  | 0.46 | 0.91 | 1.94 |  | 0.36 |
| Fresh/Frozen Fish | 0.10 |  | 0.78 |  |  |  | 0.05 |
| Groundnuts |  |  |  |  | 0.60 | 0.34 |  |
| Legumes |  | 0.42 |  |  | 0.45 | 0.13 |  |
| Mango |  |  | 0.23 |  |  |  |  |
| Okra |  |  |  |  |  | 0.58 |  |
| Oranges |  |  |  |  |  | 1.21 |  |
| Papaya |  |  | 0.75 |  |  |  |  |
| Pumpkin |  |  | 0.12 |  |  |  |  |
| Small Dried Fish | 0.04 | 0.15 | 0.06 | 0.05 | 0.07 |  | 0.02 |
| Small Tinned Fish | 0.09 | 0.54 | 0.68 | 0.28 |  |  |  |
| Sweet Potato |  |  | 0.04 |  |  |  |  |
| Yogurt/Sour Milk | 0.60 |  | 0.81 | 0.88 | 1.26 |  | 0.47 |

Notes: DGLV = Dark Green Leafy Vegetables.

*Chicken and chicken liver portion sizes vary depending on the purchasable form (alive vs. dead, whether liver is purchased separately or with the chicken). Units are in kilograms except for eggs (number of eggs), fresh milk (liters), and yogurt/sour milk (liters).

# Supplementary text – average share of micronutrient requirements analysis details

Average share of micronutrient requirements is a metric that we developed to assess the affordability of a food in terms of its provision of multiple micronutrients combined. The impetus for conducting this analysis is that we found several foods that are a good source of several micronutrients but are considered relatively unaffordable when assessed in terms of each micronutrient individually. For a given portion size *i,* of a given food *j,* the average share of requirements over a set of micronutrients *A,* is calculated as:

$$X_{i,j}=\frac{1}{|A|}\sum_{a \in A} min\{\frac{nutrient\_density_{a,j}*i}{nutrient\_requirements_{a}}, 1\}$$

For each food in the analysis, we calculated the portion size *i* for which the average share of requirements equals one third and assessed the affordability of those foods for which this portion size was less than or equal to 100 g.

For this analysis, which is focused on complementary feeding in Eastern and Southern Africa, we chose micronutrients with common potential gaps across the region (iron, vitamin A, calcium, zinc, folate, and vitamin B_12_), and we used the daily requirements of a nutrient from complementary foods, adjusting for the micronutrient contributions from breastmilk ^12^. However, this metric could easily be extended to other contexts by changing the set of nutrients analyzed and incorporating population-specific nutrient requirements.

Figures S1-S3 further demonstrate how the average share of requirements is calculated across a range of portion sizes. Figures S1 and S2 show the proportion of daily nutrient needs that are met from portion sizes of different foods for individual micronutrients. Figure S3 shows the average share of micronutrient requirements of these foods and portions. The levels shown in Figure S3 can be visualized as the averages of the individual micronutrient adequacy levels shown in Figures S1 and S2. The foods included in the average share of micronutrient requirements analysis are those for which the height of the colored lines exceeds the height of the horizontal dashed black line (at 33.3%) at portion sizes of 100 g or lower. Table S6 shows the nutrient densities used in this analysis; these are the same nutrient densities used in the by-nutrient affordability analysis.

# Table S8: Nutrients and foods which are affordable and unaffordable under alternative thresholds

|  | **Protein** | **Iron** | **Vitamin A** | **Calcium** | **Zinc** | **Folate** | **Vitamin B_12_** |
| --- | --- | --- | --- | --- | --- | --- | --- |
| **Affordable (at a threshold of less than 5% of total adjusted household food expenditure)** | | | | | | | |
| **Ethiopia** | x | x | Beef Liver, Dark Green Leafy Veg., Carrots, Pumpkin, Chicken Liver | x | x |  |  |
| **Mozambique** | x | x | Beef Liver, Sweet Potato, Chicken Liver, Dark Green Leafy Veg. |  |  |  |  |
| **South Africa** | x | x | Beef Liver, Chicken Liver, Carrots | x |  |  |  |
| **Tanzania** | Small Dried Fish, Fresh/Frozen Fish | x | Beef Liver, Carrots, Chicken Liver, Dark Green Leafy Veg., Mango, Fresh Milk | Small Dried Fish |  |  |  |
| **Uganda** | x | x | Beef Liver, Carrots, Pumpkin, Chicken Liver, Dark Green Leafy Veg., Mango | x |  |  |  |
| **Zambia** | x | x | Beef Liver, Pumpkin, Chicken Liver, Carrots, Dark Green Leafy Veg. | x | x | x | Beef Liver, Chicken Liver |
| **Affordable (at a threshold of less than 10% of total adjusted household food expenditure)** | | | | | | | |
| **Ethiopia** | Cottage Cheese | Dark Green Leafy Veg. | Beef Liver, Dark Green Leafy Veg., Carrots, Pumpkin, Chicken Liver, Fresh Milk | Dark Green Leafy Veg. | x |  |  |
| **Mozambique** | Small Dried Fish, Fresh/Frozen Fish | x | Beef Liver, Sweet Potato, Chicken Liver, Dark Green Leafy Veg., Mango, Small Dried Fish |  |  |  |  |
| **South Africa** | Chicken, Small Tinned Fish | x | Beef Liver, Chicken Liver, Carrots, Pumpkin | x |  |  |  |
| **Tanzania** | Small Dried Fish, Fresh/Frozen Fish, Fresh Milk, Beef | Legumes | Beef Liver, Carrots, Chicken Liver, Dark Green Leafy Veg., Mango, Fresh Milk, Papaya, Small Dried Fish | Small Dried Fish, Fresh Milk |  |  |  |
| **Uganda** | Small Dried Fish, Fresh Milk, Fresh/Frozen Fish, Beef | Legumes | Beef Liver, Carrots, Pumpkin, Chicken Liver, Dark Green Leafy Veg., Mango, Fresh Milk, Papaya, Small Dried Fish | Small Dried Fish |  |  |  |
| **Zambia** | x | x | Beef Liver, Pumpkin, Chicken Liver, Carrots, Dark Green Leafy Veg. | x | x | x | Beef Liver, Chicken Liver, Fresh/Frozen Fish, Small Dried Fish |
| **Unaffordable (at a threshold of greater than 50% of total adjusted household food expenditure)** | | | | | | | |
| **Ethiopia** | x | Beef, Chicken Liver | x | x | Chicken, Chicken Liver, Eggs |  |  |
| **Mozambique** | Fresh Milk | Beef | Fresh/Frozen Fish |  |  |  |  |
| **South Africa** | x | Beef, Dark Green Leafy Veg., Small Tinned Fish | Small Tinned Fish | Dark Green Leafy Veg. |  |  |  |
| **Tanzania** | x | Beef | x | x |  |  |  |
| **Uganda** | x | Beef | Fresh/Frozen Fish | x |  |  |  |
| **Zambia** | x | Beef, Fresh Milk, Small Dried Fish | Sour Milk, Fresh/Frozen Fish | Fresh Milk, Sour Milk | Chicken, Chicken Liver, Eggs, Fresh Milk, Groundnuts, Sour Milk | Bananas, Eggs, Oranges | x |

Note: This table shows the foods for each nutrient and country combination that cost less than 1%, 5%, and 10% and greater than 50% of total household food expenditure per adult equivalent on average. Foods are ordered from least to most expensive. An “x” indicates that no foods were affordable (or unaffordable) under a given threshold, while grey shading indicates that the particular nutrient was not analyzed for a country. Vitamin A tends to be the most affordable nutrient, with several options that cost just 1% of household food expenditure or less on average (beef liver, dark green leafy vegetables, chicken liver, and/or orange-fleshed vegetables such as carrot, pumpkin, or sweet potato)

# Table S9: Relationships between food insecurity and total household food expenditure per adult equivalent in South Africa and Tanzania

| **South Africa** | | | | | | | |
| --- | --- | --- | --- | --- | --- | --- | --- |
| **Outcomes** | | | **Risk of food insecurity outcome relative to households in quintile 5** | | | | **Proportion of Households in Quintile 5** |
|  |  |  | **Quintile 1** | **Quintile 2** | **Quintile 3** | **Quintile 4** |  |
| (1) | Household's self-assessed standard of food consumption in past month was inadequate | | 2.22***  (1.73 - 2.84) | 1.98*** (1.51 - 2.59) | 1.87*** (1.44 - 2.43) | 1.35**  (1.02 - 1.79) | 0.18***  (0.14 - 0.22) |
| (2) | In the past 12 months, household ran out of money to buy food | | 2.24***  (1.83 - 2.74) | 1.96*** (1.59 - 2.42) | 1.88*** (1.54 - 2.31) | 1.60*** (1.29 - 1.98) | 0.23***  (0.19 - 0.27) |
| (3) | In the past 12 months, because there was not enough food: | Adult in household went hungry often/always | 4.53***  (2.71 - 7.55) | 3.78*** (2.24 - 6.37) | 3.53*** (2.13 - 5.84) | 2.16*** (1.27 - 3.68) | 0.03***  (0.02 - 0.05) |
| (4) |  | Child in household went hungry often/always | 6.82***  (3.56 - 13.07) | 5.15*** (2.66 - 9.99) | 4.97*** (2.61 - 9.45) | 2.41** (1.22 - 4.76) | 0.02***  (0.01 - 0.03) |
| (5) |  | Household cut size of meals | 2.35***  (1.91 - 2.90) | 2.12*** (1.70 - 2.65) | 1.85*** (1.49 - 2.30) | 1.66*** (1.31 - 2.09) | 0.21***  (0.17 - 0.25) |
| (6) |  | Household skipped meals | 2.68***  (2.09 - 3.45) | 2.15*** (1.65 - 2.80) | 2.06*** (1.59 - 2.67) | 1.62*** (1.22 - 2.14) | 0.16***  (0.13 - 0.20) |
| (7) |  | Household ate smaller portions/less variety | 2.30***  (1.88 - 2.81) | 1.98*** (1.59 - 2.47) | 1.79*** (1.46 - 2.20) | 1.50*** (1.20 - 1.88) | 0.21***  (0.18 - 0.26) |
| (8) | Any of 2-7 occurred in the past 12 months | | 1.80***  (1.53 - 2.11) | 1.60*** (1.34 - 1.90) | 1.52*** (1.29 - 1.79) | 1.33*** (1.12 - 1.59) | 0.33***  (0.28 - 0.37) |
| (9) | In the past month, on 5 or more days: | Household ran out of money to buy food | 3.05***  (2.10 - 4.43) | 2.75*** (1.90 - 3.98) | 2.39*** (1.65 - 3.47) | 1.88*** (1.25 - 2.82) | 0.08***  (0.06 - 0.11) |
| (10) |  | Household cut size of meals | 2.82***  (1.95 - 4.07) | 2.65*** (1.83 - 3.85) | 2.23*** (1.55 - 3.23) | 1.74*** (1.16 - 2.60) | 0.09***  (0.06 - 0.12) |
| (11) |  | Household skipped meals | 3.78***  (2.46 - 5.80) | 3.09*** (1.99 - 4.78) | 2.80*** (1.82 - 4.30) | 2.03*** (1.26 - 3.28) | 0.06***  (0.04 - 0.09) |
| (12) |  | Household ate smaller portions/less variety | 3.16***  (2.25 - 4.42) | 2.93*** (2.06 - 4.17) | 2.58*** (1.85 - 3.59) | 2.04*** (1.41 - 2.96) | 0.08***  (0.06 - 0.11) |

| **Tanzania** | | | | | | | |
| --- | --- | --- | --- | --- | --- | --- | --- |
| **Outcomes** | | | **Risk of food insecurity outcome relative to households in quintile 5** | | | | **Proportion of Households in Quintile 5** |
|  |  |  | **Quintile 1** | **Quintile 2** | **Quintile 3** | **Quintile 4** |  |
| (1) | Household worried they would not have enough food in past week | | 1.87***  (1.44 - 2.44) | 1.30*  (0.98 - 1.73) | 1.16  (0.83 - 1.62) | 0.92  (0.64 - 1.32) | 0.29***  (0.23 - 0.36) |
| (2) | In the past week, household had to: | Rely on less preferred foods | 1.79***  (1.28 - 2.49) | 1.16  (0.82 - 1.65) | 1.27  (0.88 - 1.83) | 1.04  (0.69 - 1.57) | 0.25***  (0.19 - 0.32) |
| (3) |  | Limit food variety | 1.56*  (0.94 - 2.58) | 0.82  (0.44 - 1.54) | 1.35  (0.79 - 2.30) | 0.98  (0.53 - 1.78) | 0.15***  (0.10 - 0.22) |
| (4) |  | Limit portion sizes | 3.23***  (1.85 - 5.63) | 1.59  (0.86 - 2.93) | 1.75*  (0.92 - 3.34) | 1.57  (0.83 - 2.97) | 0.09***  (0.06 - 0.14) |
| (5) |  | Reduce number of meals | 2.92***  (1.86 - 4.58) | 2.19*** (1.36 - 3.53) | 1.82**  (1.08 - 3.06) | 1.59*  (0.94 - 2.69) | 0.11***  (0.08 - 0.17) |
| (6) |  | Reduce consumption by adults for small children to eat | 4.24***  (1.44 - 12.49) | 2.89*  (0.94 - 8.91) | 2.41  (0.73 - 7.88) | 2.76*  (0.93 - 8.22) | 0.03***  (0.01 - 0.10) |
| (7) |  | Borrow food or rely on help from a friend or relative | 2.57**  (1.23 - 5.34) | 2.23**  (1.09 - 4.54) | 2.34**  (1.16 - 4.69) | 1.84  (0.85 - 3.99) | 0.06***  (0.04 - 0.12) |
| (8) |  | Have no food of any kind in the household | 2.19*  (0.99 - 4.85) | 1.57  (0.70 - 3.51) | 1.17  (0.43 - 3.21) | 1.12  (0.41 - 3.06) | 0.05***  (0.03 - 0.10) |
| (9) |  | Go a whole day and night without eating | 17.51*** (3.35 - 91.54) | 5.56*  (0.89 -34.54) | 6.33**  (1.02 - 39.11) | 6.72*  (0.99 - 45.78) | 0.00***  (0.00 - 0.02) |
| (10) | Household did not have enough food in the past year | | 2.06***  (1.48 - 2.87) | 1.76*** (1.28 - 2.42) | 1.74***  (1.23 - 2.47) | 1.16  (0.83 - 1.64) | 0.27***  (0.20 - 0.36) |
| (11) | Any of 1-10 applied to the household | | 1.51***  (1.22 - 1.87) | 1.33*** (1.09 - 1.64) | 1.34**  (1.06 - 1.69) | 1.00  (0.77 - 1.30) | 0.45***  (0.37 - 0.53) |

Note: This table shows the results of using Poisson regressions to regress food insecurity outcomes on dummy variables for household quintile. The resulting coefficients are interpretable as the risk of a household in a particular decile experiencing a particular food insecurity condition, relative to households in the highest quintile, and the constant is interpretable as the proportion of households in the highest quintile experiencing that condition. Sample sizes were 3,226-3,303 for South Africa and 1,061-1,062 for Tanzania (some slight variation by indicator). 95% confidence intervals are shown in parentheses. *** p<0.01, ** p<0.05, * p<0.1 .

# Supplementary Text – Vitamin A supplementation affordability comparison

*Methods*

We compared the affordability of obtaining vitamin A from complementary foods with the affordability of vitamin A supplementation. We identified published estimates of the costs of vitamin A capsules and their delivery through a government program in Sub-Saharan Africa. We assumed that supplementation would be delivered through a government program based on current policy (vitamin A supplementation is typically delivered through a country’s Expanded Program of Immunization) and because vitamin A capsules are generally not available for households to purchase on the market.

Details of these cost estimates are shown in Table 10. For comparability across estimates, we show the costs in 2018 United States Dollars (USD). Across the studies, vitamin A supplementation delivery costs are estimated to range from $1.54 in Senegal (not including capsules, which cost around $0.02 to $0.05) to $4.76 in South Africa. To conduct the affordability analysis, we converted the estimates to local currency units from the years covered by the consumption and expenditure surveys. We compared these costs to household food expenditure per AEQ over a six-month period, for comparability with the affordability analysis of nutritious foods and since vitamin A supplementation programs generally deliver a six-month dose. When country-specific vitamin A supplementation costs were available (as for South Africa and Zambia), we used those costs. When they were not, we used a range covering countries of similar income levels (Table 11).

# Table S10: Published vitamin A supplementation cost estimates in Sub-Saharan Africa

| **Study** | **Country or Region** | **Year** | **Delivery Mode** | **Unit Cost (including delivery)** | **Currency** | **Cost in 2018 USD** | **Notes** |
| --- | --- | --- | --- | --- | --- | --- | --- |
| Neidecker-Gonzales et al ^24^ | Ghana | 2000 | Campaign | 0.51 | 2004 USD | $2.78 |  |
|  | South Africa | 1999 | Campaign | 2.27 | 2004 USD | $4.76 |  |
|  | Zambia | 2004 | Campaign | 0.61 | 2004 USD | $2.34 |  |
| Horton et al ^25^ | Senegal | 2016 | Campaign | 728.6 | 2016 FCFA | $1.54 | Does not include capsule cost |
| Kagin et al ^26^ | Cameroon | 2013 | Campaign | 1.65 | 2013 USD | $2.27 |  |
| Bhutta et al ^27^ | Sub-Saharan Africa | NA | Not specified | 2.82-2.85 | 2010 $Int | $3.25-3.28 | Costs are estimated based on ingredients approach |

Note: delivery mode indicates whether the study costed vitamin A supplementation delivered via a mass campaign - either standalone or alongside other health services (such as a Child National Health day) - compared to delivery via the routine health system. USD = United States Dollars. FCFA = Francs of the Financial Community of Africa. $Int = International Dollars.

*Results*

Across all six countries, the cost of vitamin A supplementation is estimated to fall below 5% of household food expenditure per AEQ, ranging from 0.9% to 3.6% (Table 11). This cost is comparable to the costs of several foods in the analysis, including beef liver, chicken liver, orange-fleshed vegetables and fruits (such as carrots, pumpkin, or mango), and dark green leafy vegetables. For at least three of these foods in each country, portion sizes that fulfill 50% of vitamin A requirements cost less than 3% of food expenditure per AEQ and many cost less than 1%. However, since vitamin A supplementation provides close to 100% of requirements over six months, and our affordability analysis of nutritious foods focused on portion sizes that provide 50% of requirements, it may be more appropriate to compare its costs to the costs of obtaining 100% of vitamin A requirements through complementary feeding. These affordability comparisons are shown in Table 11. For most countries, there are multiple foods that could meet 100% of vitamin A requirements at similar costs to vitamin A supplementation. Zambia is an exception, and given higher coverage of vitamin A supplementation programs in Zambia and a lack of data on beef liver consumption among children, supplementation could be a promising option, In the other countries, vitamin A supplementation could be another affordable alternative to consuming vitamin A through complementary foods, although the interpretation of affordability here is different, since governments, not households, generally cover the cost of vitamin A supplementation programs.

Coverage of vitamin A supplementation programs across Eastern, Southern, and Central Africa averages only 68%, with higher coverage in Zambia and Tanzania and lower coverage (less than 60%) in Mozambique, South Africa, and Ethiopia ^28^. Additionally, while all studies considered here provided cost estimates for the delivery of vitamin A supplementation via mass campaigns, many countries are shifting toward delivery via the routine health system for budgetary reasons and because campaigns are often conducted as part of polio vaccine campaigns, which are starting to be phased out in many countries ^25^. While a shift to routine delivery may decrease costs, countries that have undergone these shifts have also experienced declines in coverage ^25^. Given current low coverage levels in several countries and the threat of further declines, consumption of a range of affordable, vitamin A-rich foods may be a more feasible, safer, and more sustainable alternative to ensuring children of complementary feeding age receive adequate vitamin A. On the other hand, for countries with higher coverage, such as Zambia and Tanzania, where there appears to be a gap in vitamin A consumption despite its affordability, it is possible that vitamin A supplementation will be a feasible alternative to behavior change interventions that may be necessary to fill that gap via changes in children’s diets.

# Table S11: Vitamin A supplementation costs and affordability

|  | Ethiopia | Mozambique | South Africa | Tanzania | Uganda | Zambia |
| --- | --- | --- | --- | --- | --- | --- |
| Vitamin A Supplementation | | | | | | |
| Unit Cost | 34.6-70.6 | 68.3-139.4 | 53.7 | 3,142-6,411 | 4,924-10,048 | 18.2 |
| Cost per Food Expenditure per AEQ | 1.1% - 2.4% | 1.7% - 3.6% | 2.5% - 2.8% | 0.9% - 2.0% | 1.5% - 3.4% | 1.9% - 2.2% |
| Complementary Foods (cost of 100% requirements divided by food expenditure per AEQ) | | | | | | |
| Beef Liver | 0.5% [0.5-0.5] | 0.7% [0.7-0.8] | 0.7% [0.6-0.7] | 0.4% [0.4-0.4] | 0.5% [0.5-0.5] | 1.5% [1.3-1.6] |
| Chicken Liver | 4.0% [3.8-4.1] | 1.7% [1.6-1.8] | 0.9% [0.9-1.0] | 2.3% [2.1-2.4] | 2.4% [2.2-2.5] | 3.8% [3.3-4.2] |
| DGLV | 1.5% [1.4-1.6] | 5.9% [4.9-6.9] | 20.9% [19.2-22.6] | 2.4% [2.2-2.7] | 3.1% [2.7-3.4] | 6.4% [5.7-7.1] |
| Orange-Fleshed Vegetables | 0.8% [0.7-0.8] | 1.0% [0.9-1.0] | 2.5% [2.3-2.7] | 1.1% [1.0-1.2] | 1.1% [1.0-1.2] | 3.5% [3.1-3.9] |

“DGLV” = Dark Green Leafy Vegetables. Costs are calculated in local currency units from the year from which household food expenditure data was collected. Affordability ranges for vitamin A supplementation incorporate both ranges in costs (for some countries) and uncertainty in the mean estimate of household food expenditure. For foods, means with 95% confidence intervals are shown. The most affordable orange-fleshed fruit/vegetable is shown for each country: carrots (Ethiopia, South Africa, Tanzania, Uganda), orange-fleshed sweet potato (Mozambique), pumpkin (Zambia).

# Figure S1: Share of micronutrient requirements across portion sizes (plant-source foods)


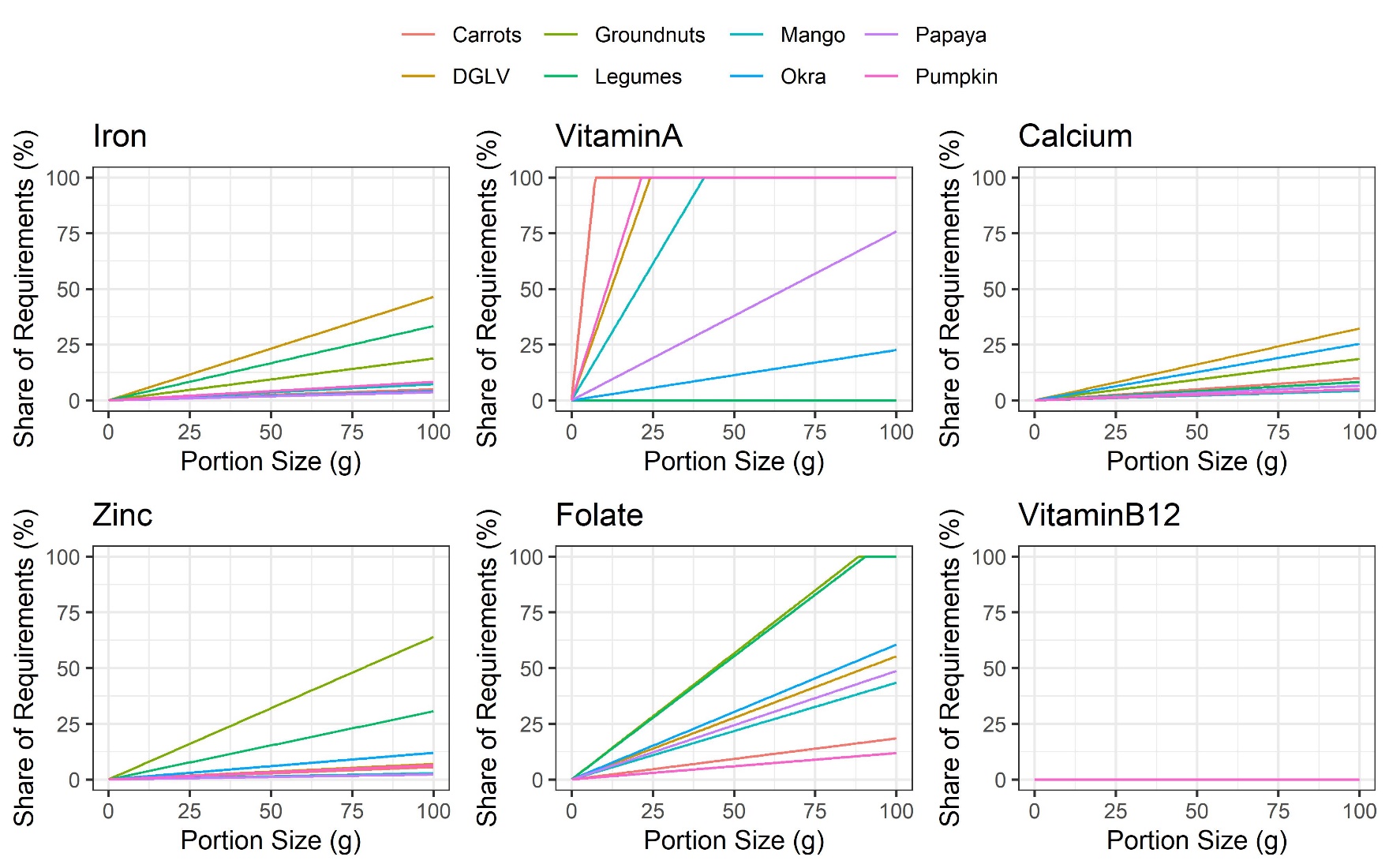


Notes: DGLV = Dark Green Leafy Vegetables

# Figure S2: Share of micronutrient requirements across portion sizes (animal-source foods)


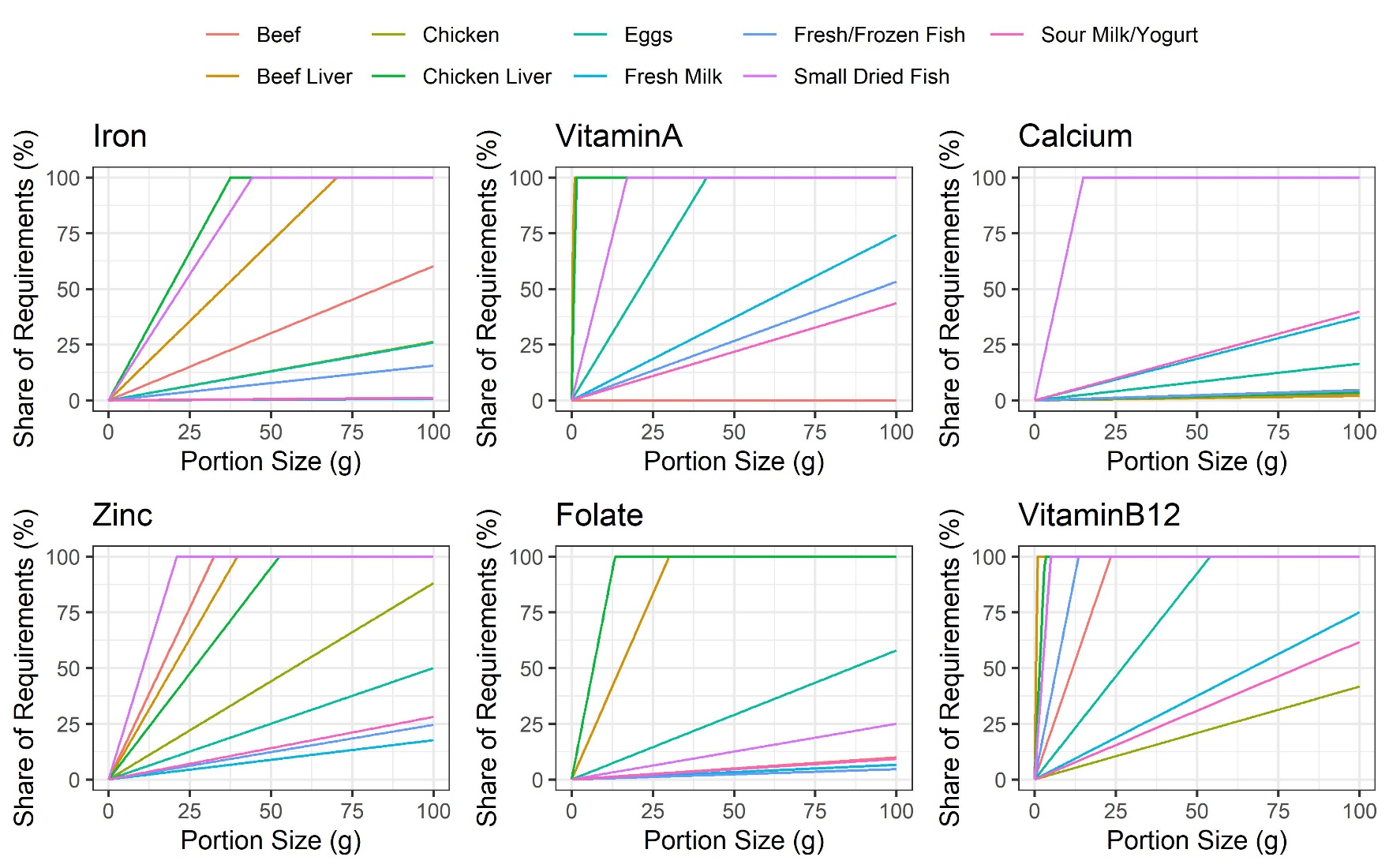


# Figure S3: Average share of micronutrient requirements across portion sizes, by food


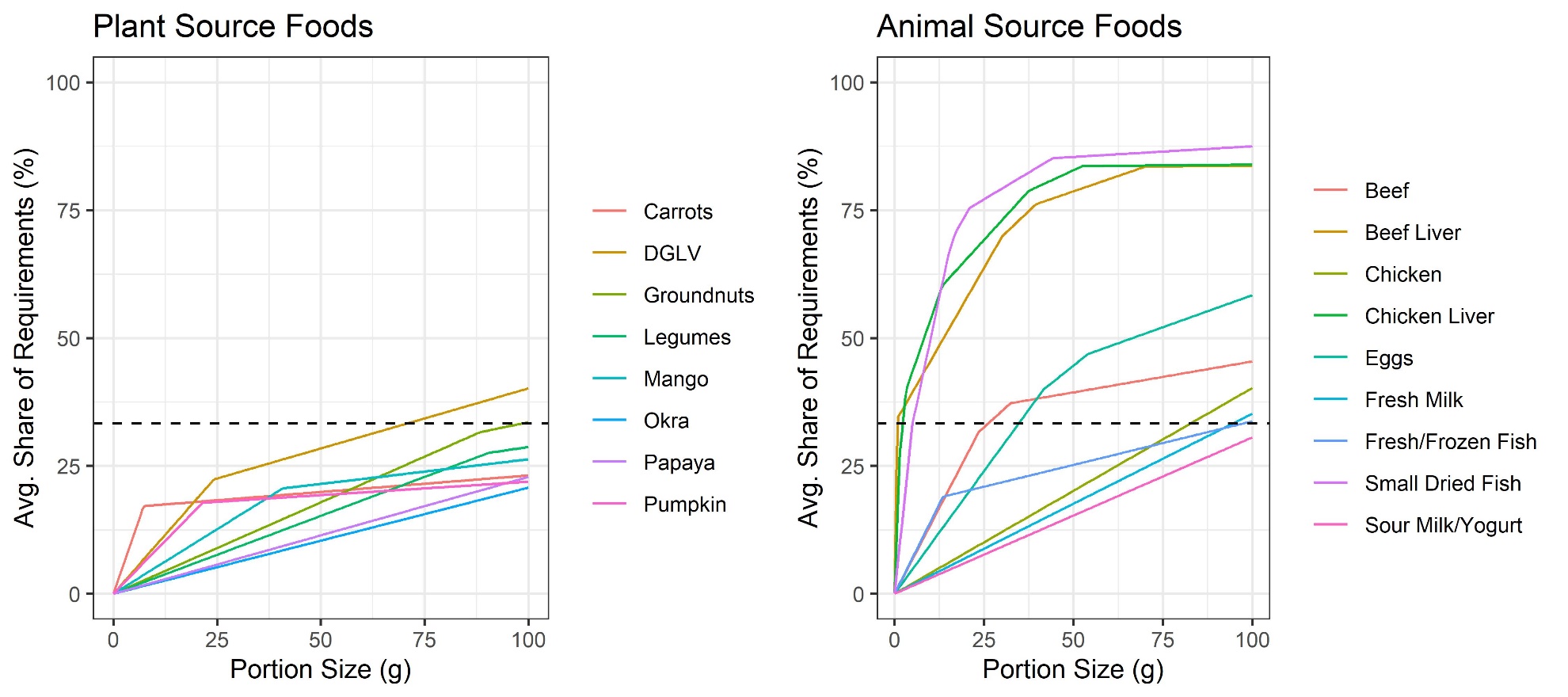


Notes: DGLV = Dark Green Leafy Vegetables

# Figure S4: Total household food and non-food expenditure, by rural/urban setting


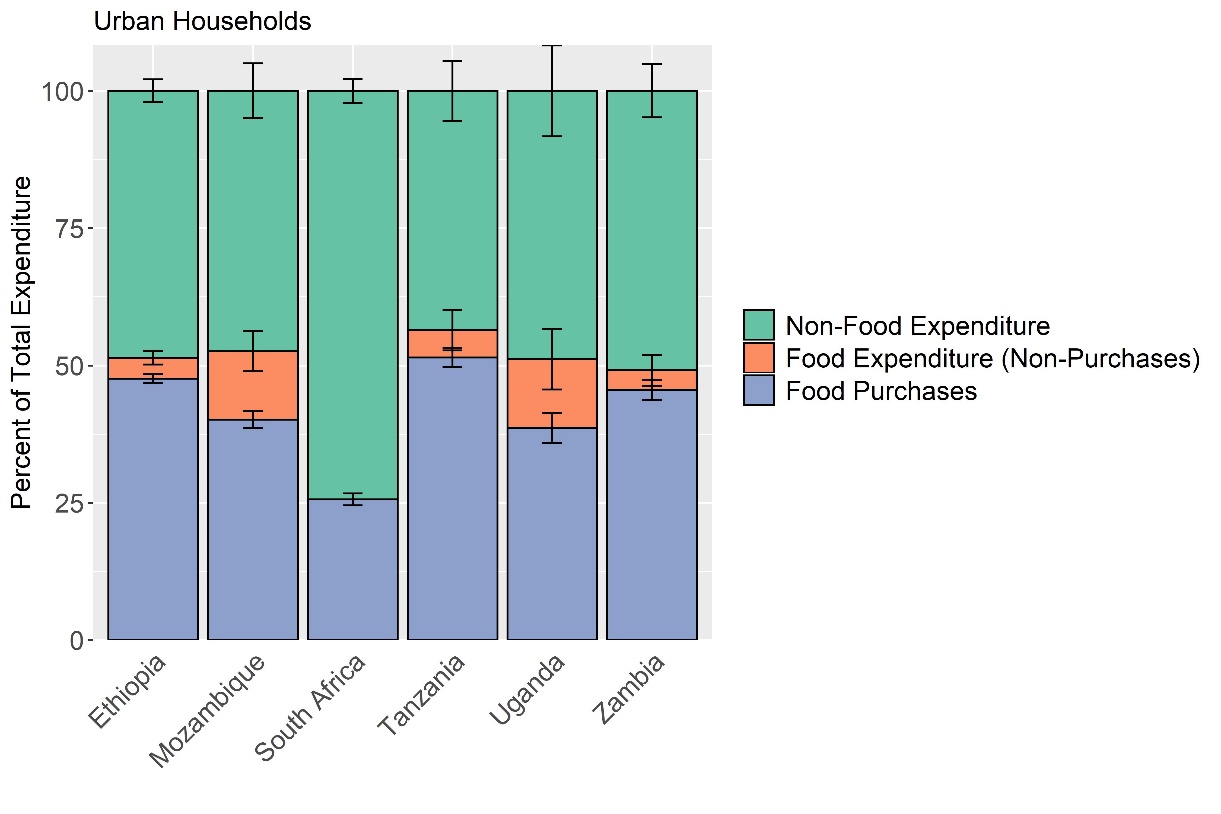

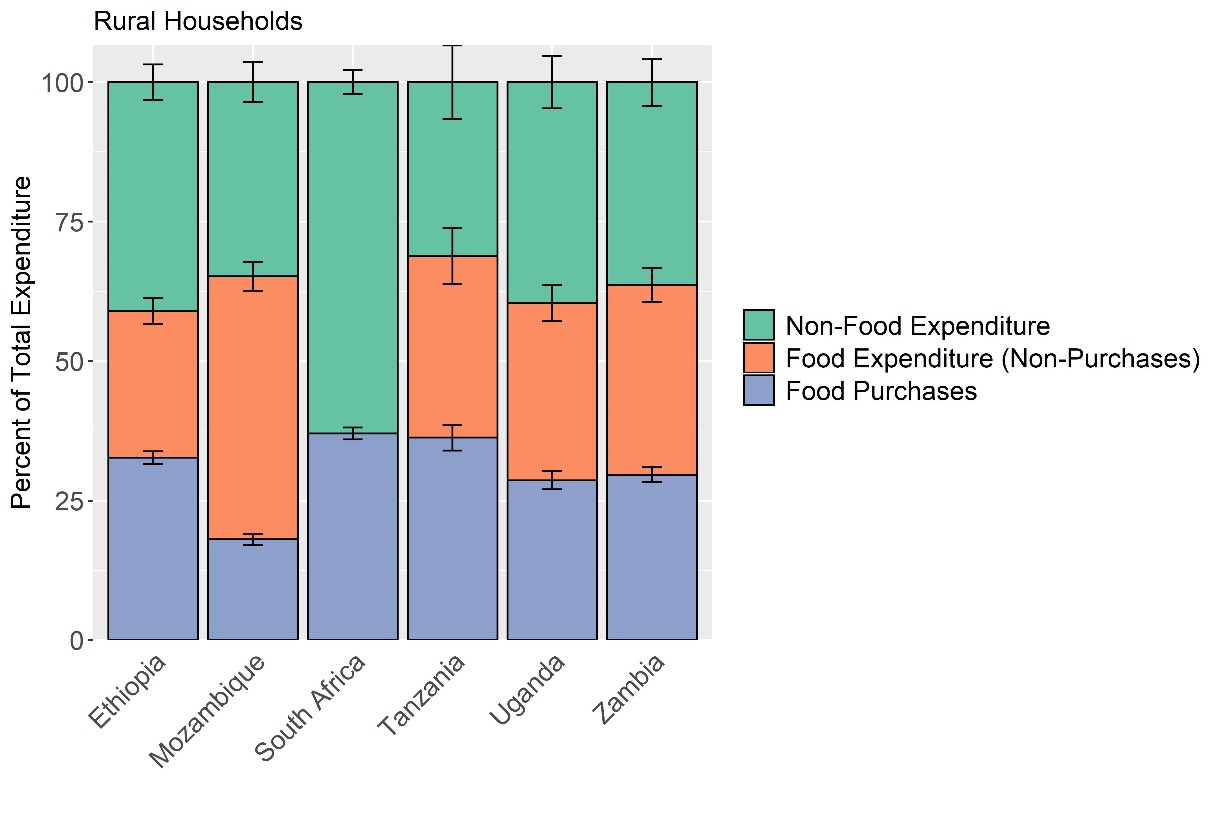


Note: breakdowns of food expenditure by purchases vs. own production/other sources were not available for South Africa. However, purchases are thought to make up the vast majority of total food expenditure given low levels of food consumption from own production in the country. For all countries, only households with children of complementary feeding age are shown.

Figure S5: Total household food and non-food expenditure, by quintile**
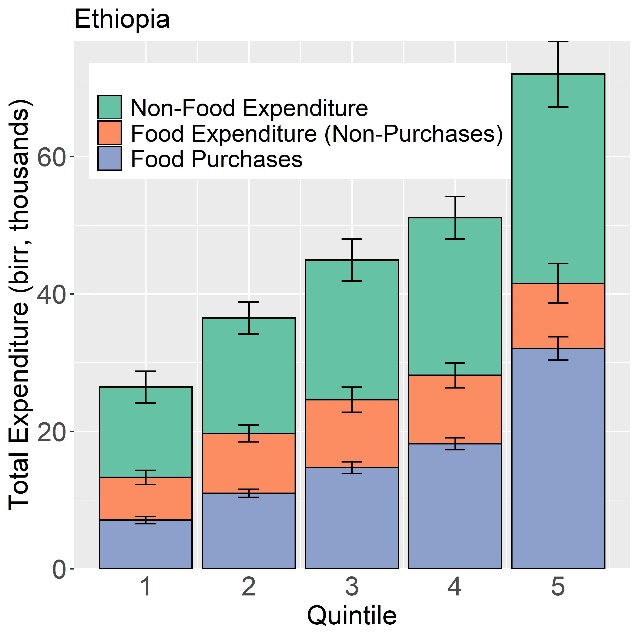

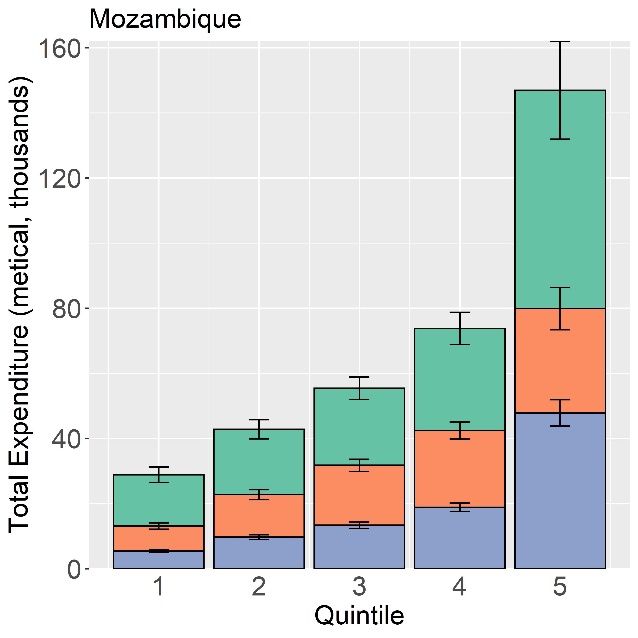

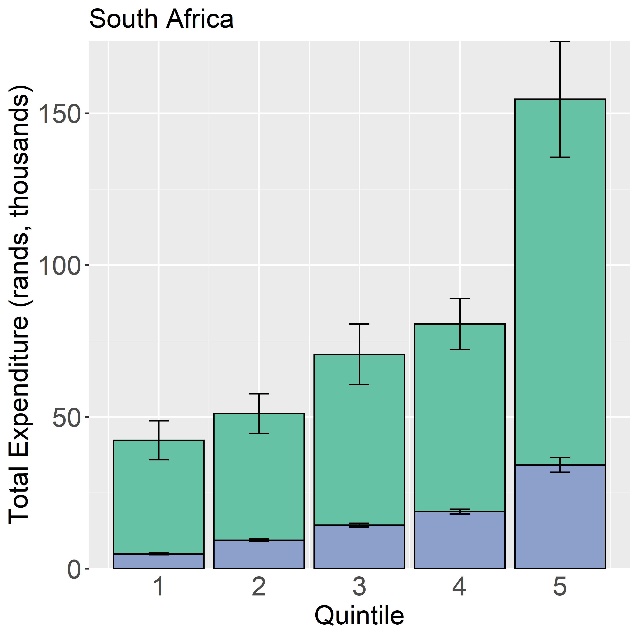

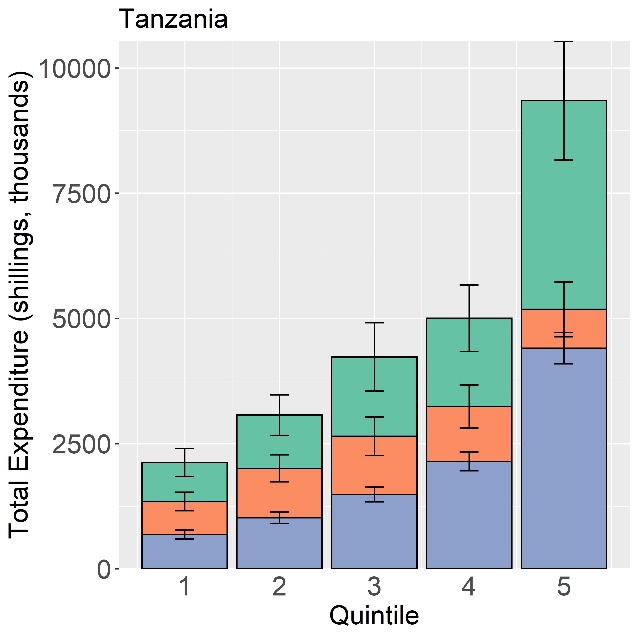
**

**
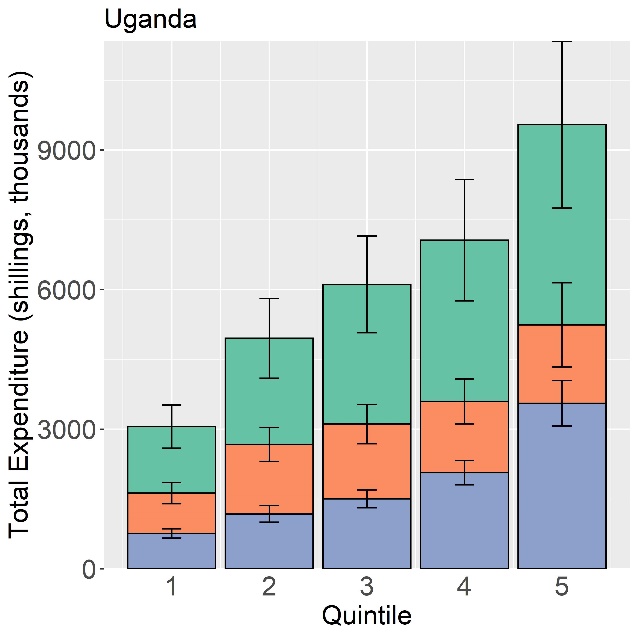

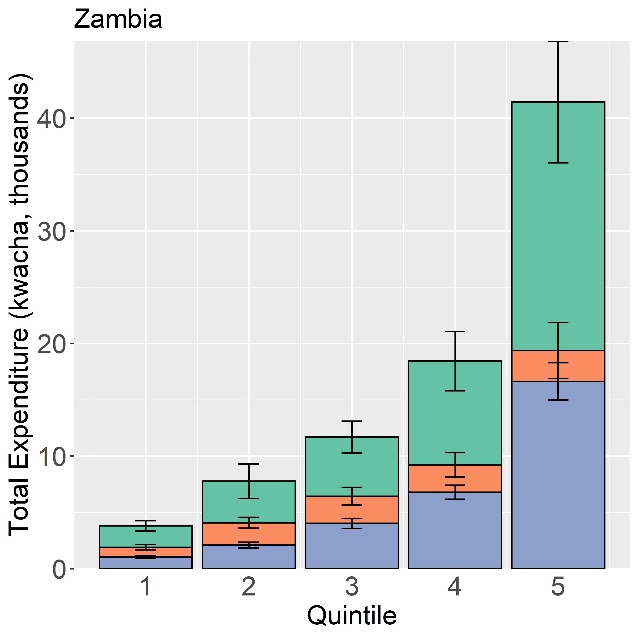
**

Note: breakdowns of food expenditure by purchases vs. own production/other sources were not available for South Africa. However, purchases are thought to make up the vast majority of total food expenditure given low levels of food consumption from own production in the country. For all countries, only households with children of complementary feeding age are shown.

# Figure S6: Household consumption of key food groups, by rural/urban setting


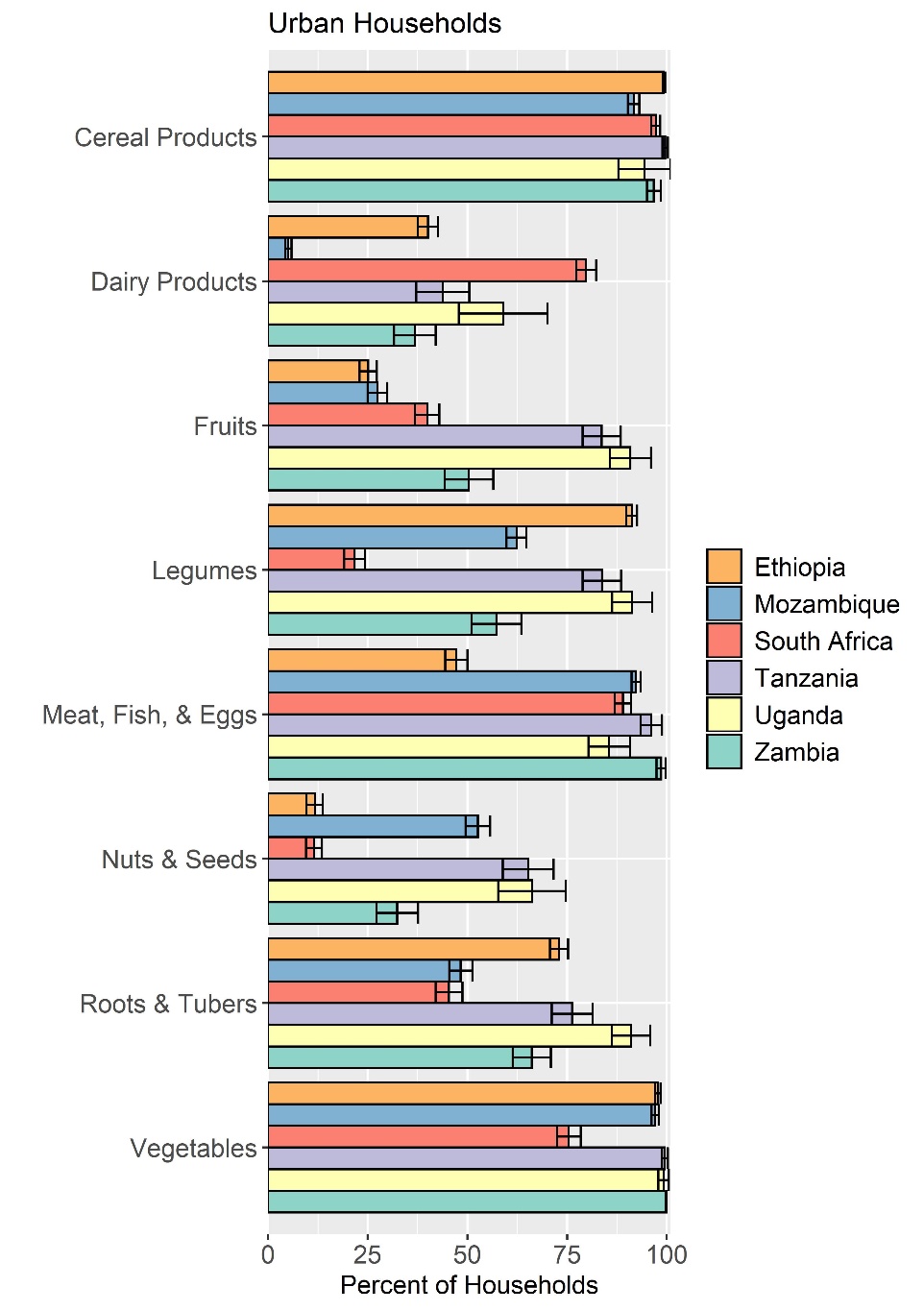

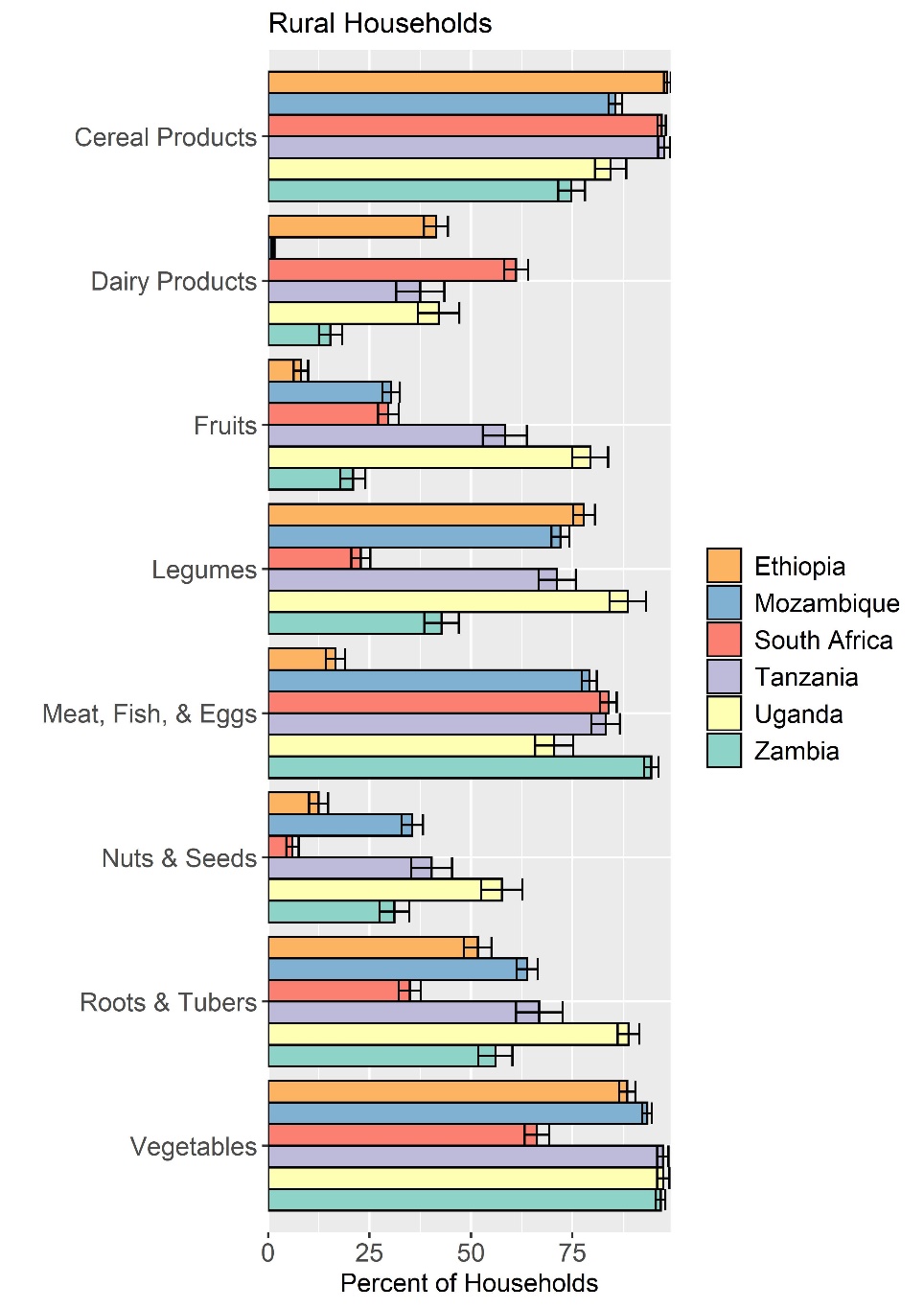


Note: only households with children of complementary feeding age are shown. Surveys covered household consumption over the past week, except for South Africa and Zambia, for which the past two weeks’ consumption was covered.

# Figure S7: Weekly household expenditure by food group and rural/urban setting


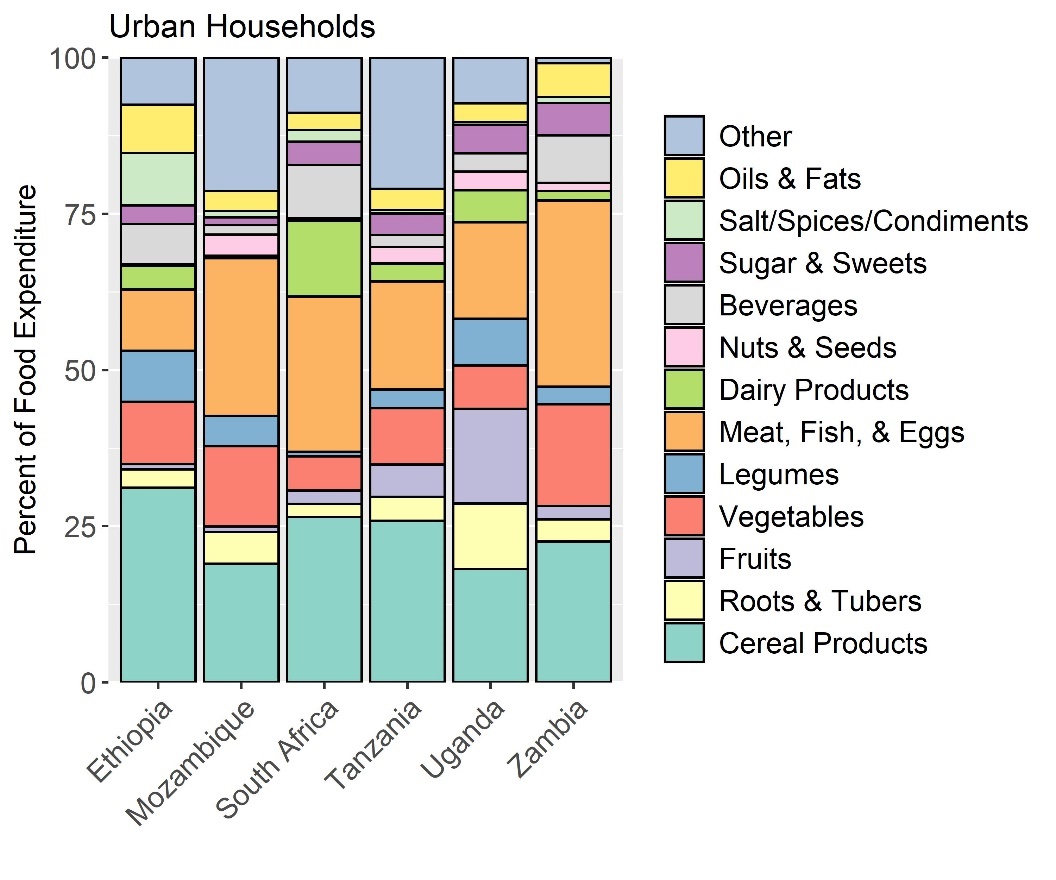

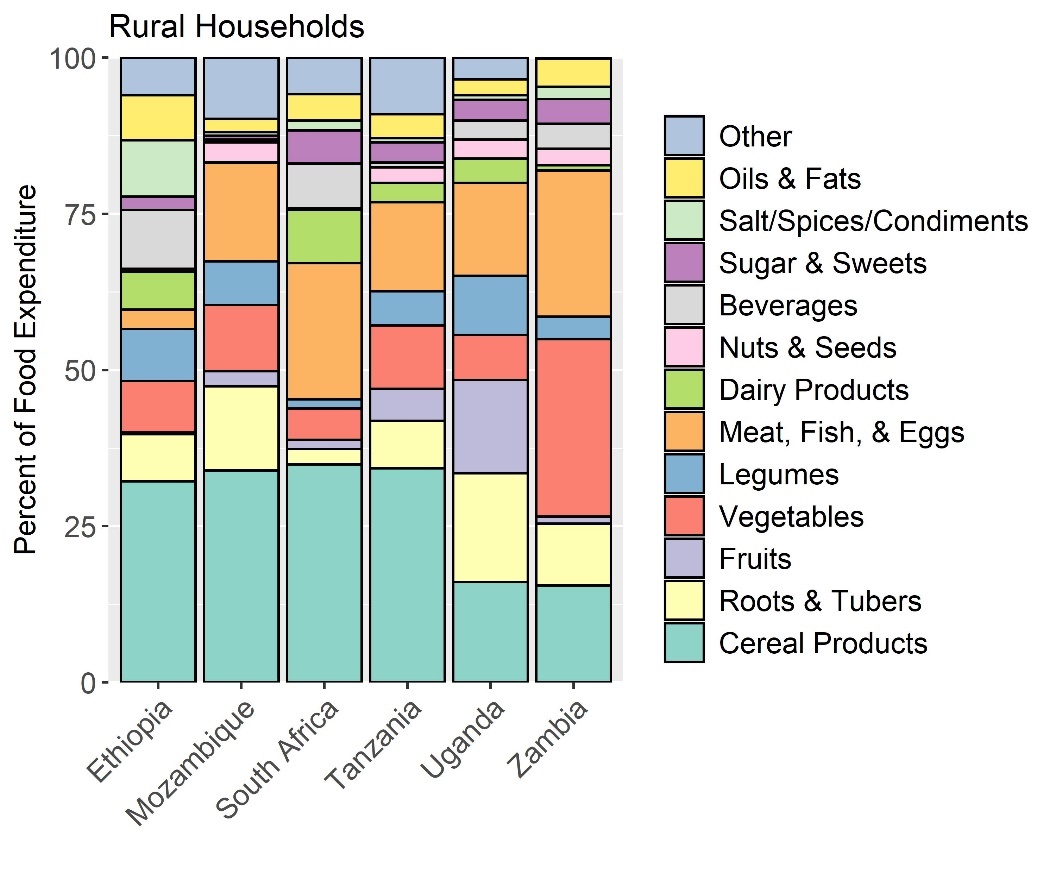


Note: only households with children of complementary feeding age are shown. Expenditure includes value of consumption from all sources (purchases, own-production, and in-kind).

# Figure S8: Household consumption of key food groups, by quintile


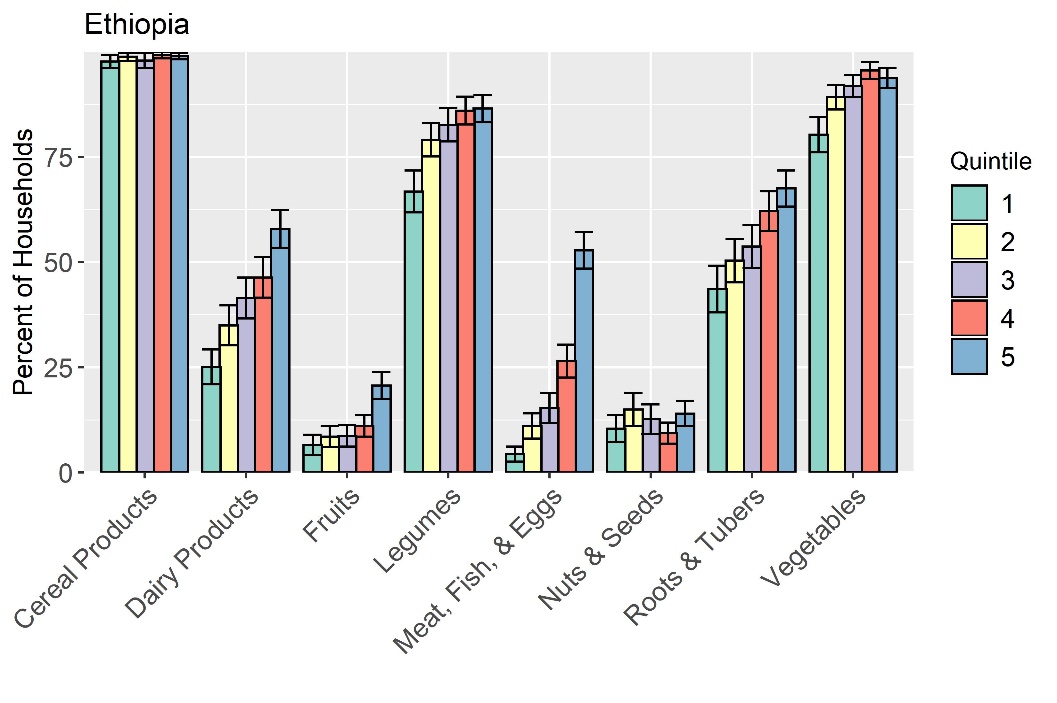

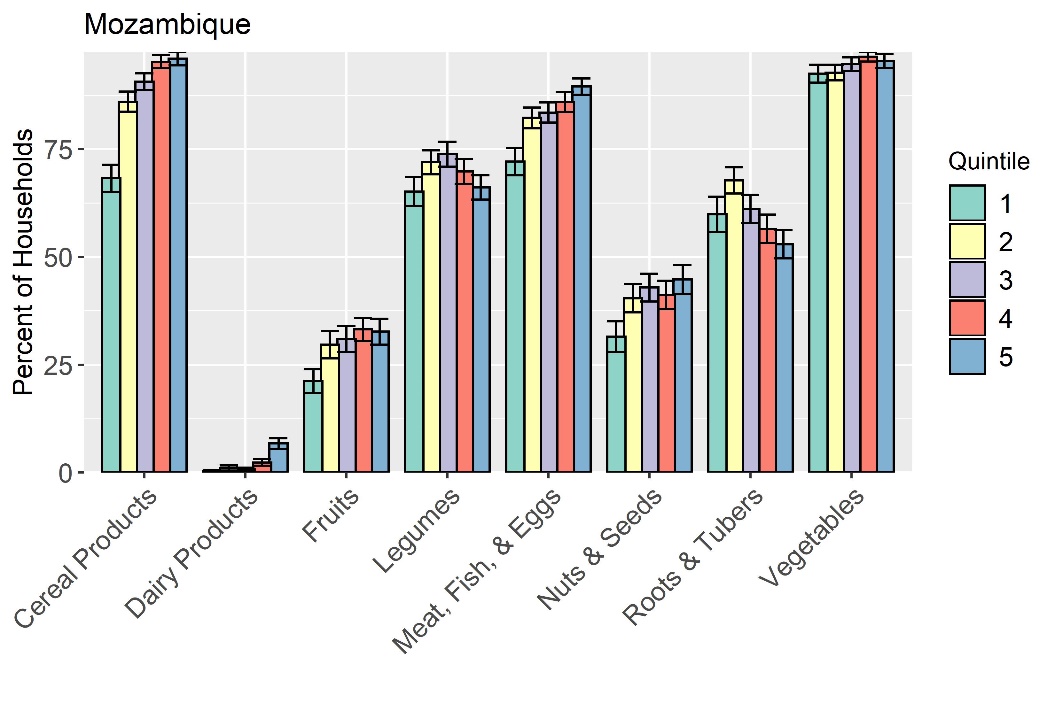

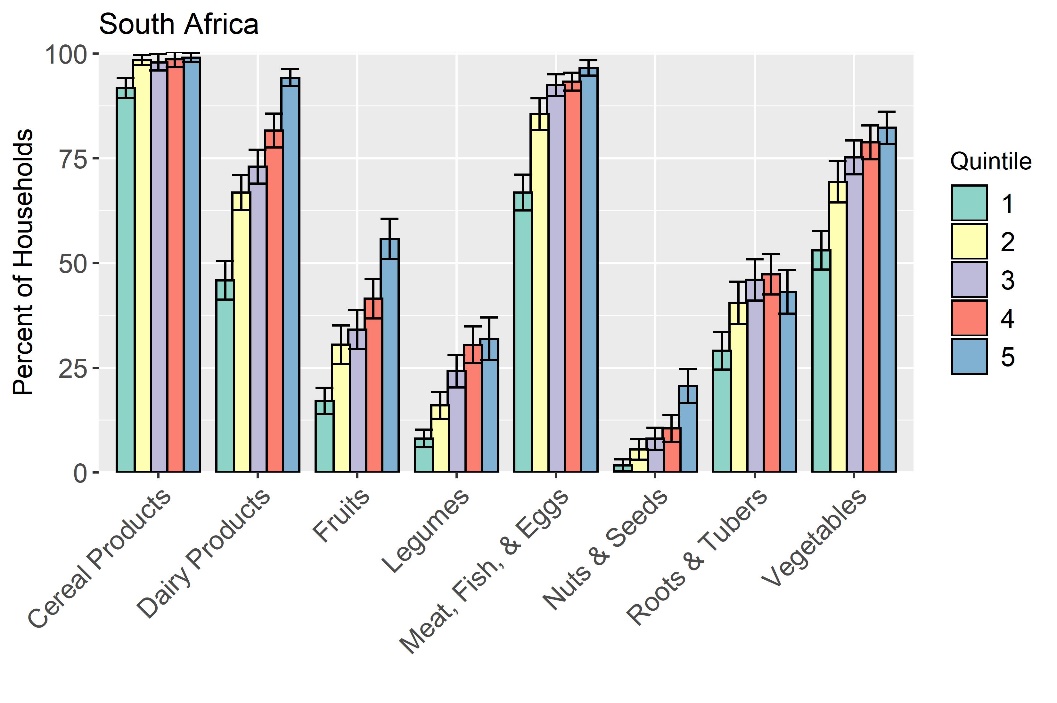

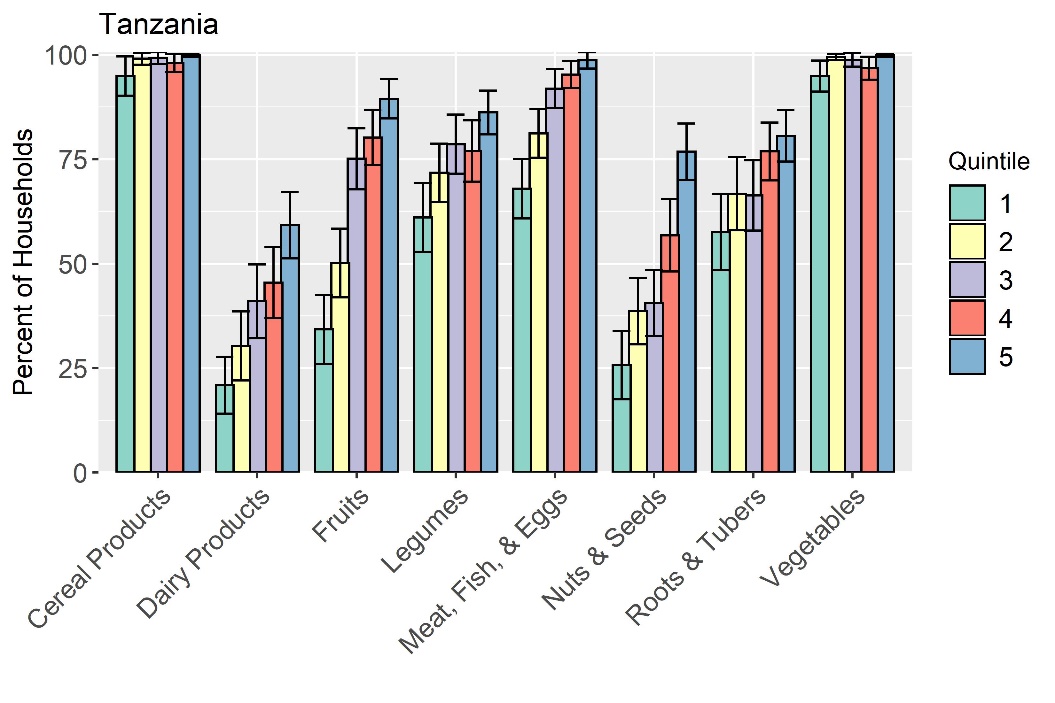


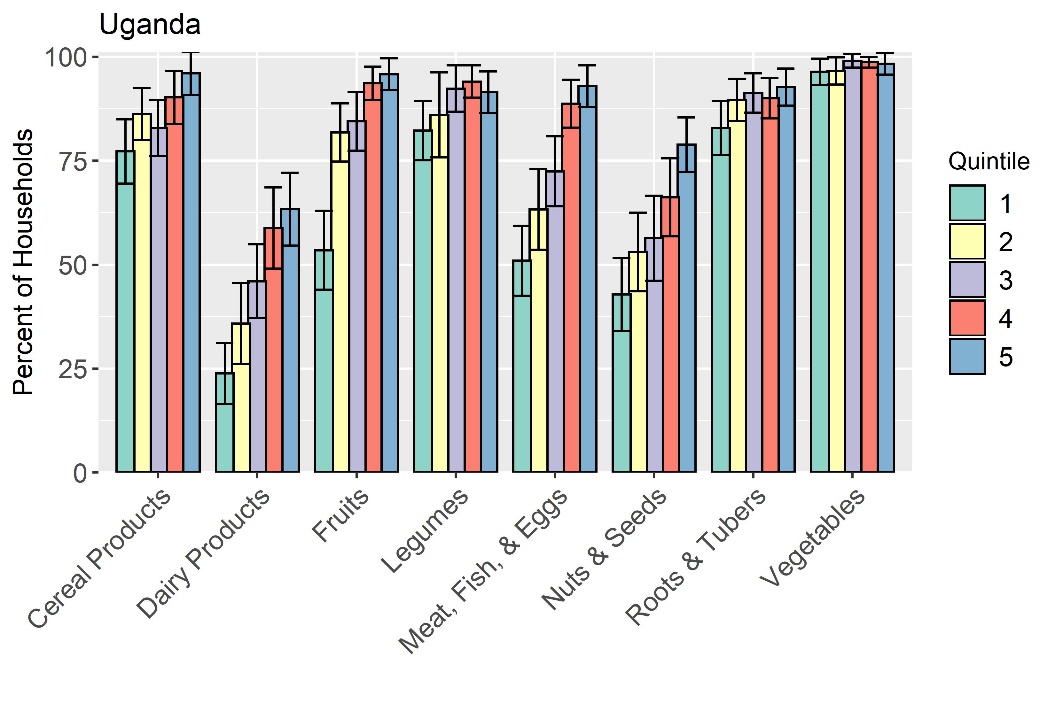

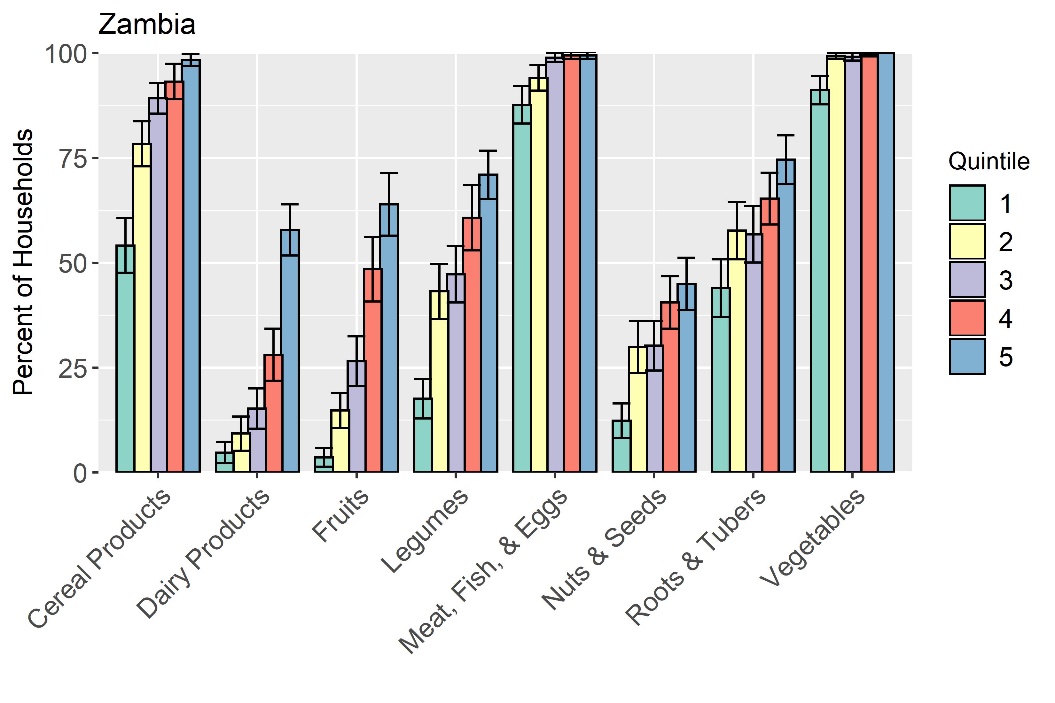


Note: only households with children of complementary feeding age are shown. Surveys covered household consumption over the past week, except for South Africa and Zambia, for which the past two weeks’ consumption was covered.

# Figure S9: Proportional weekly household expenditure by food group and quintile


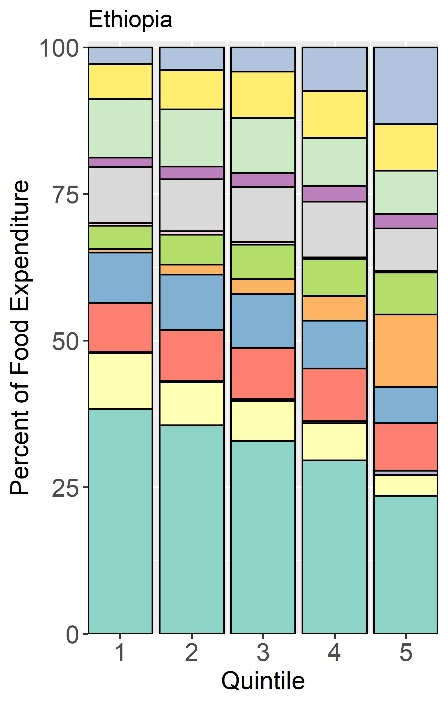

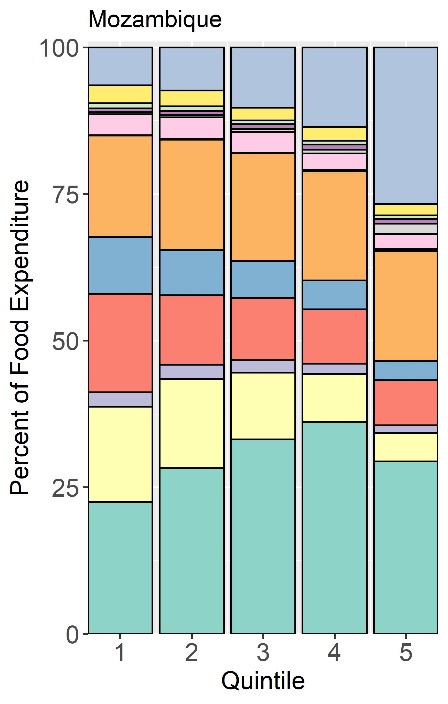

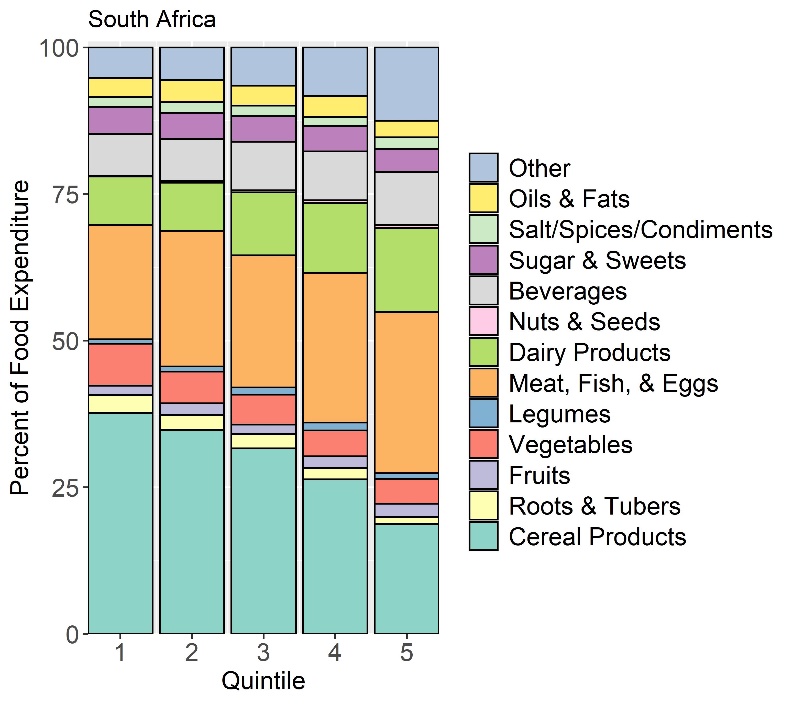

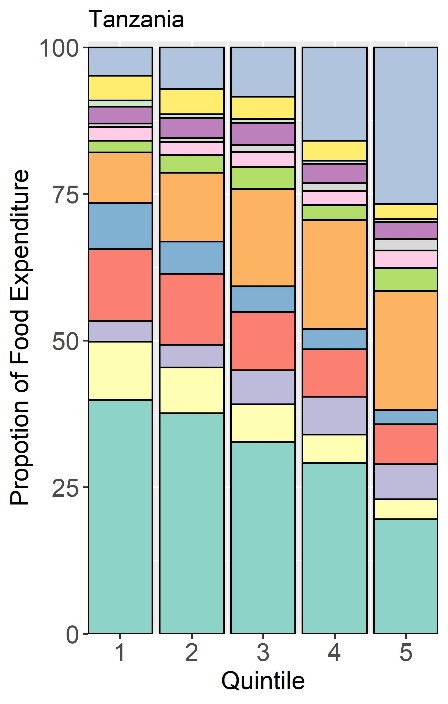

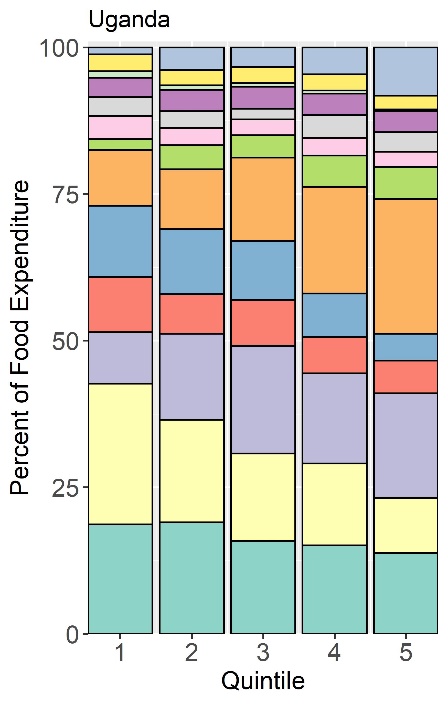

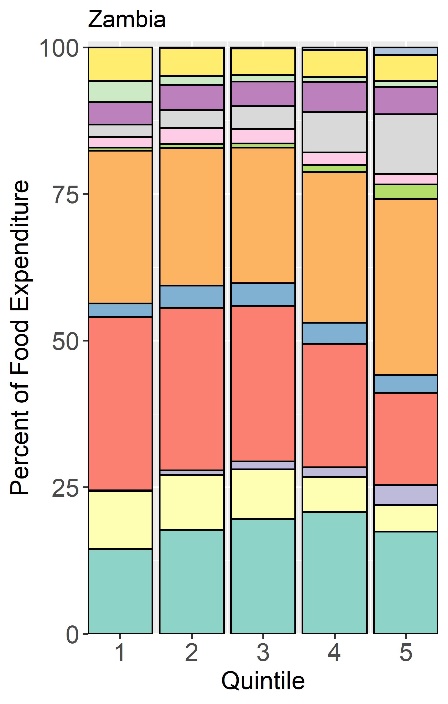


Note: only households with complementary feeding age children are shown. Expenditure includes value of consumption from all sources (purchases, own-production, in-kind).

# Figure S10: Total weekly household expenditure by food group and quintile


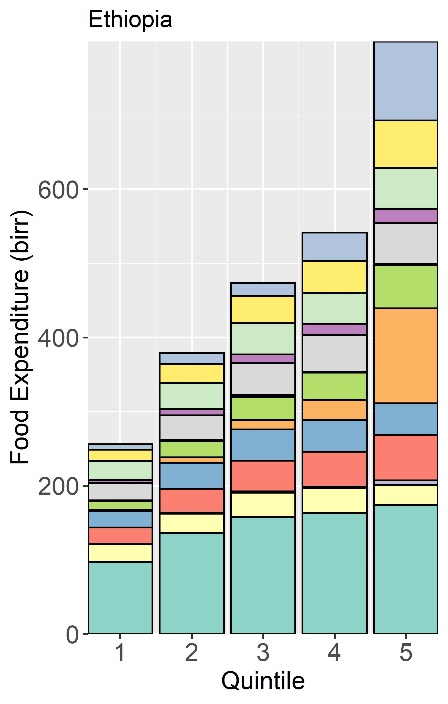

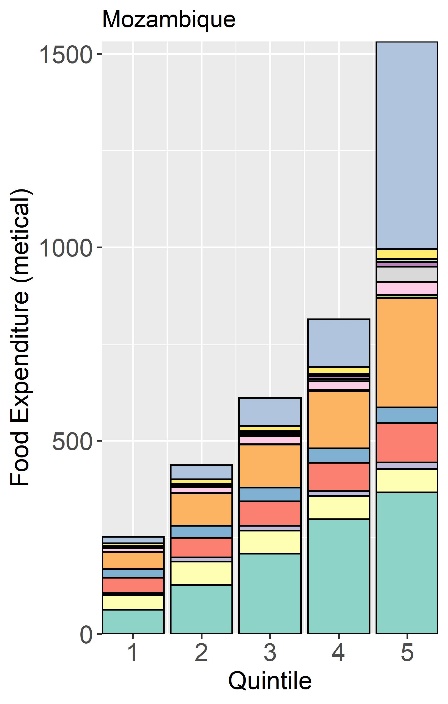

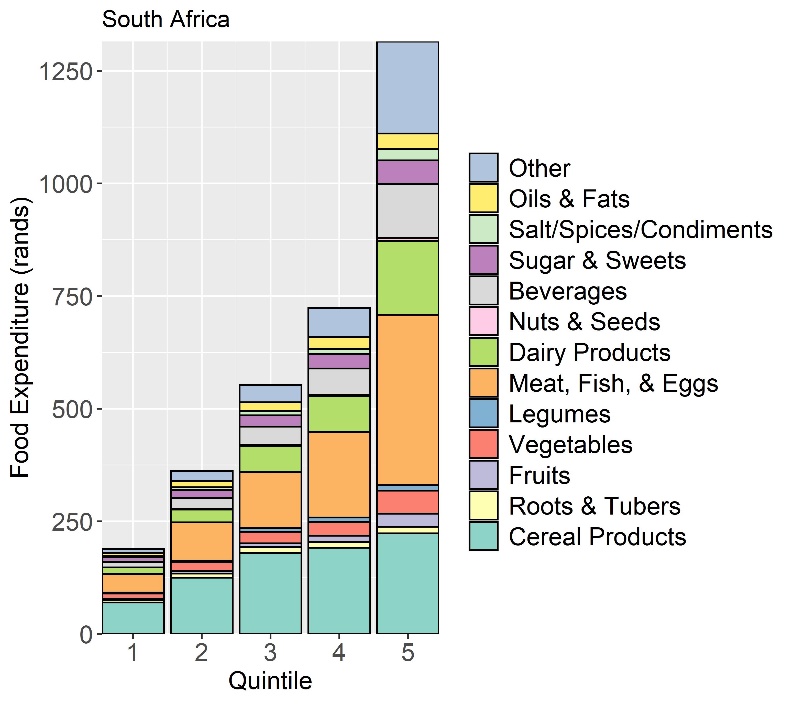

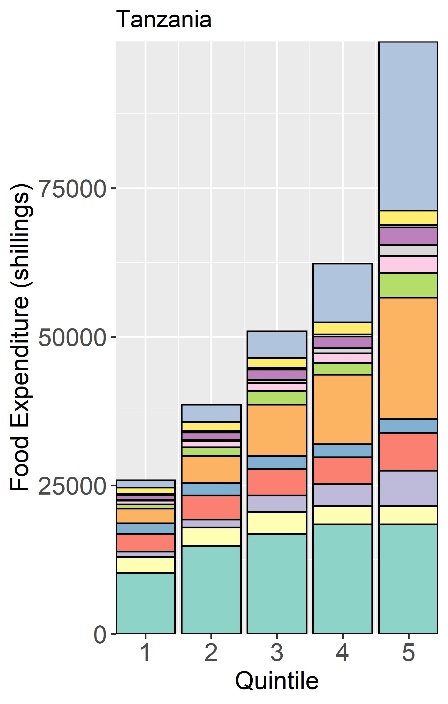

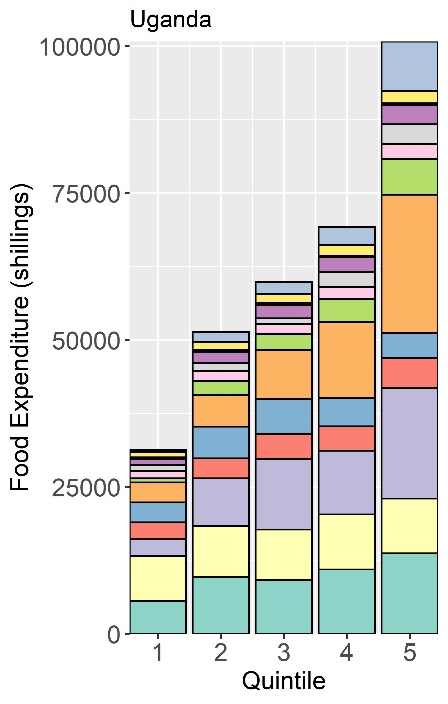

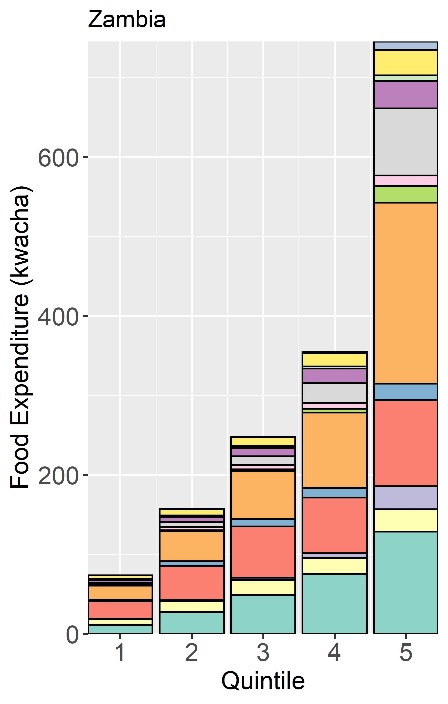


Note: only households with complementary feeding age children are shown. Expenditure includes value of consumption from all sources (purchases, own-production, in-kind).

# Figure S11: Current consumption of selected nutritious foods, by rural/urban setting


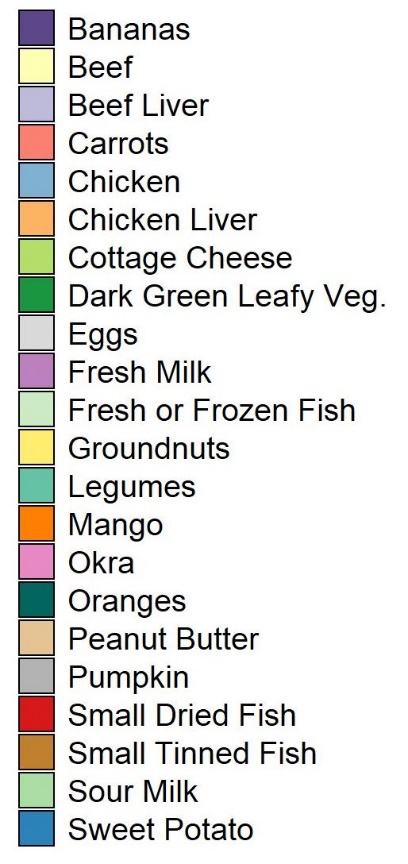

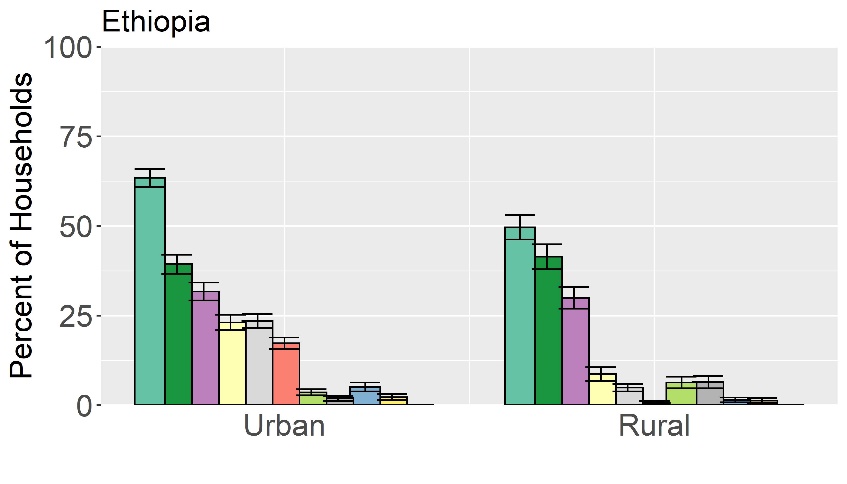

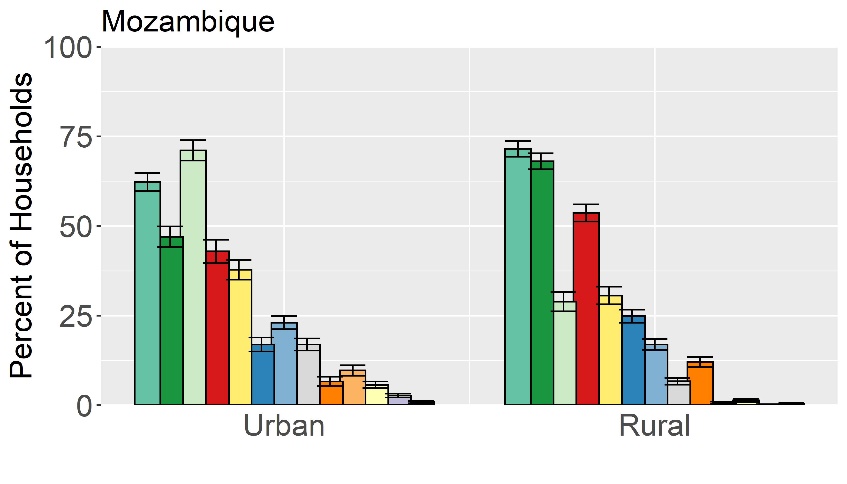

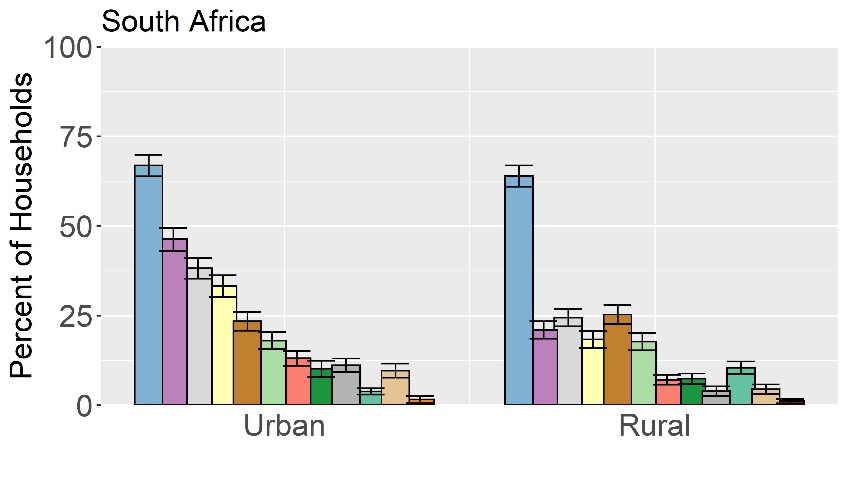

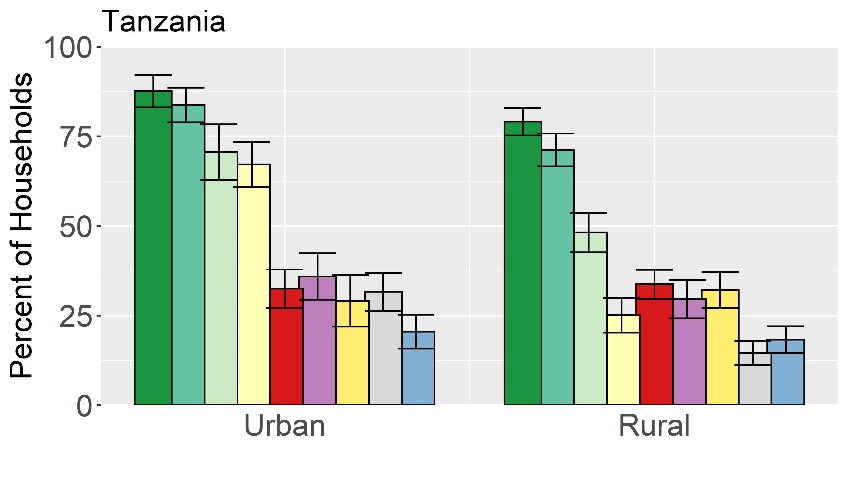

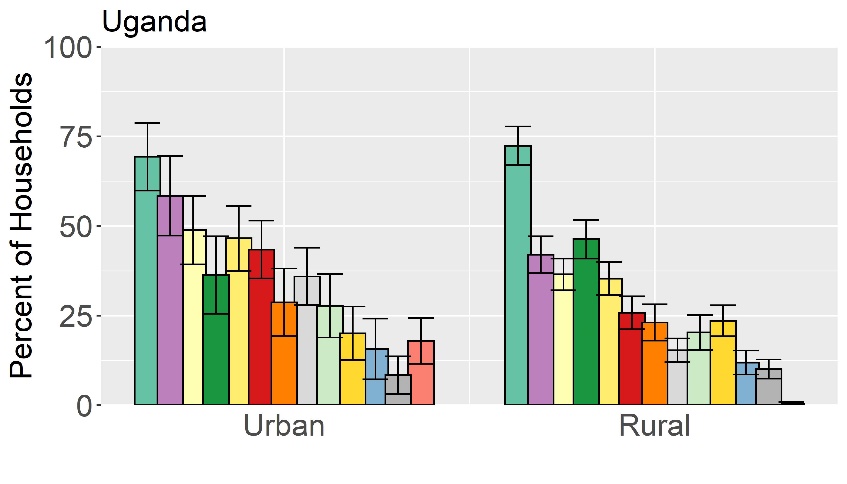

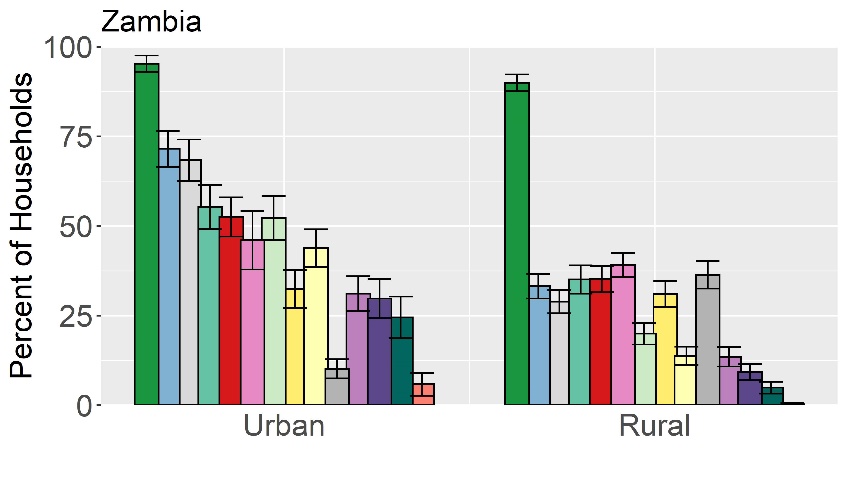


Note: only households with children of complementary feeding age are shown.

# Figure S12: Current expenditure on selected nutritious foods, by rural/urban setting


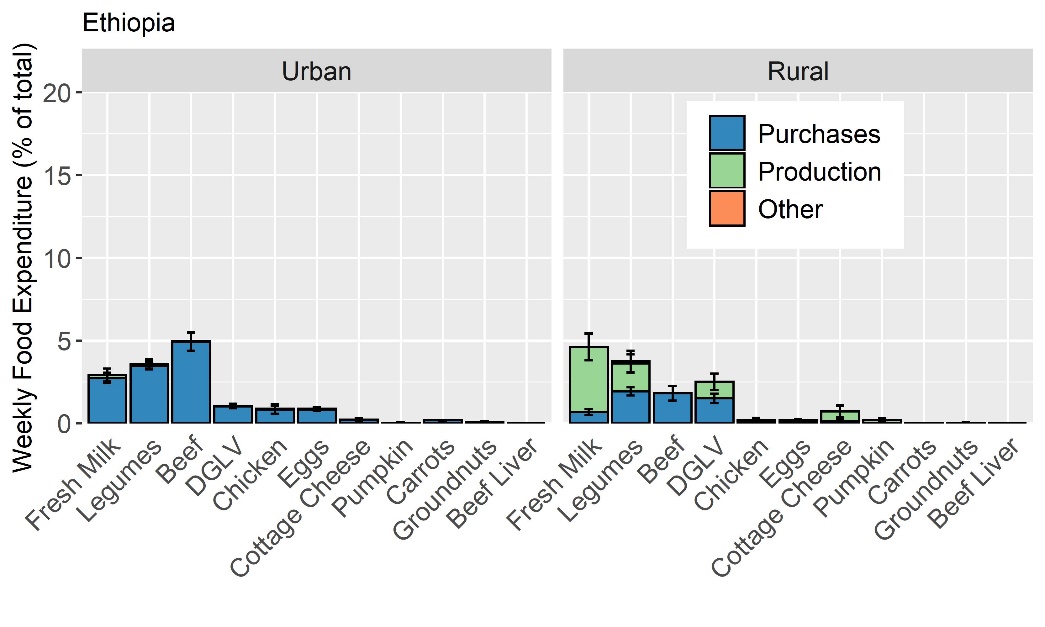

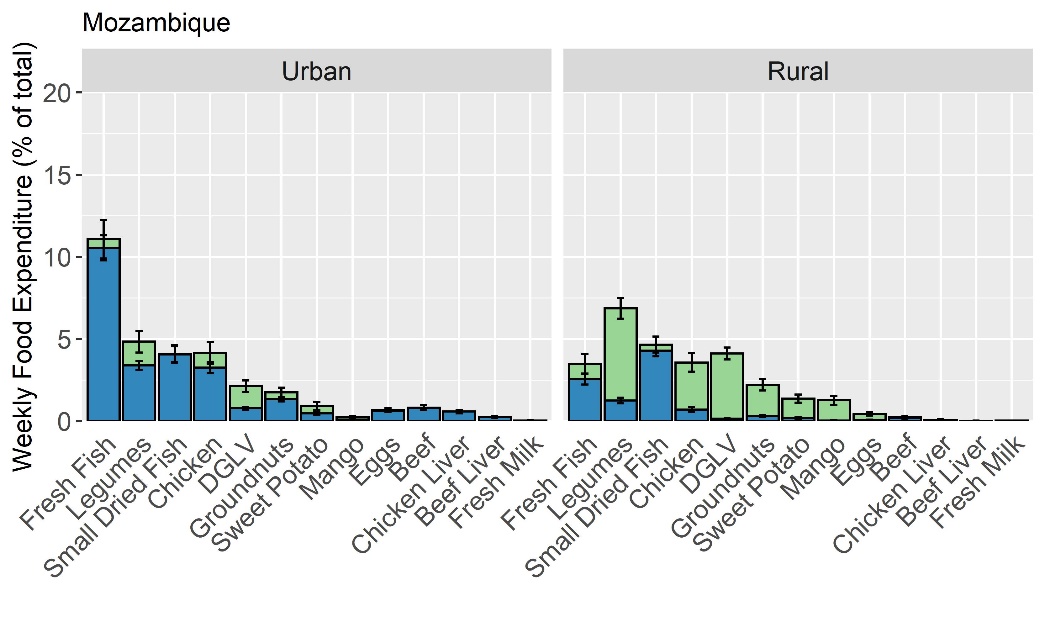

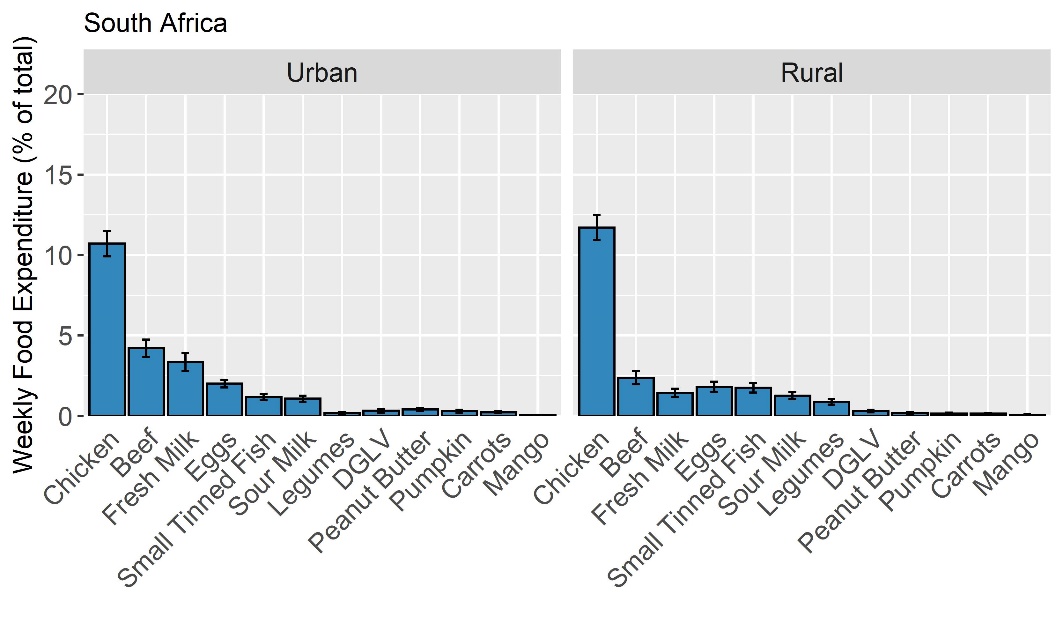

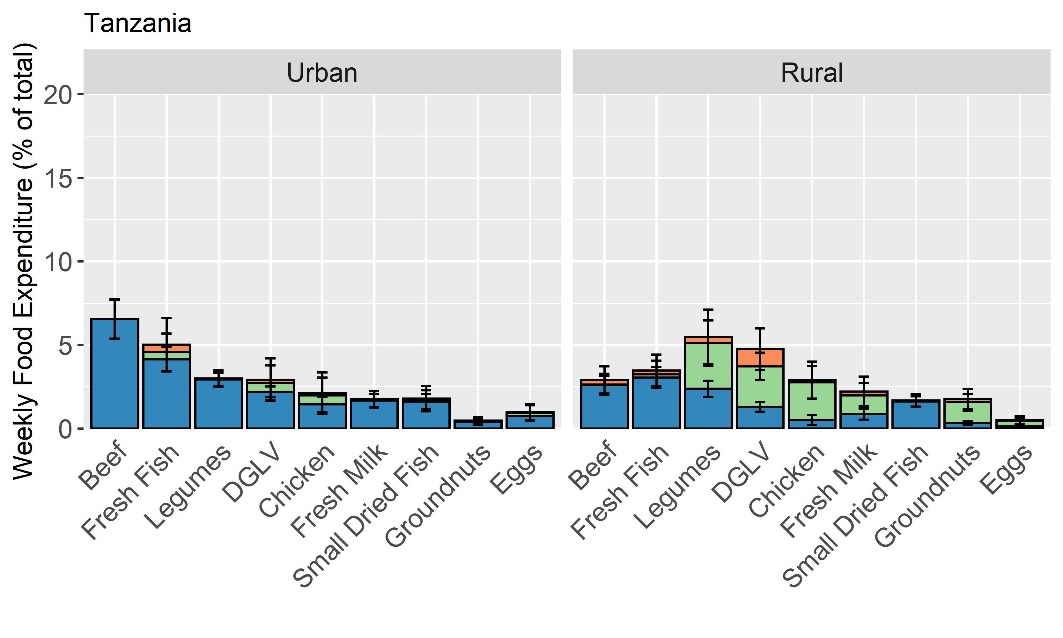

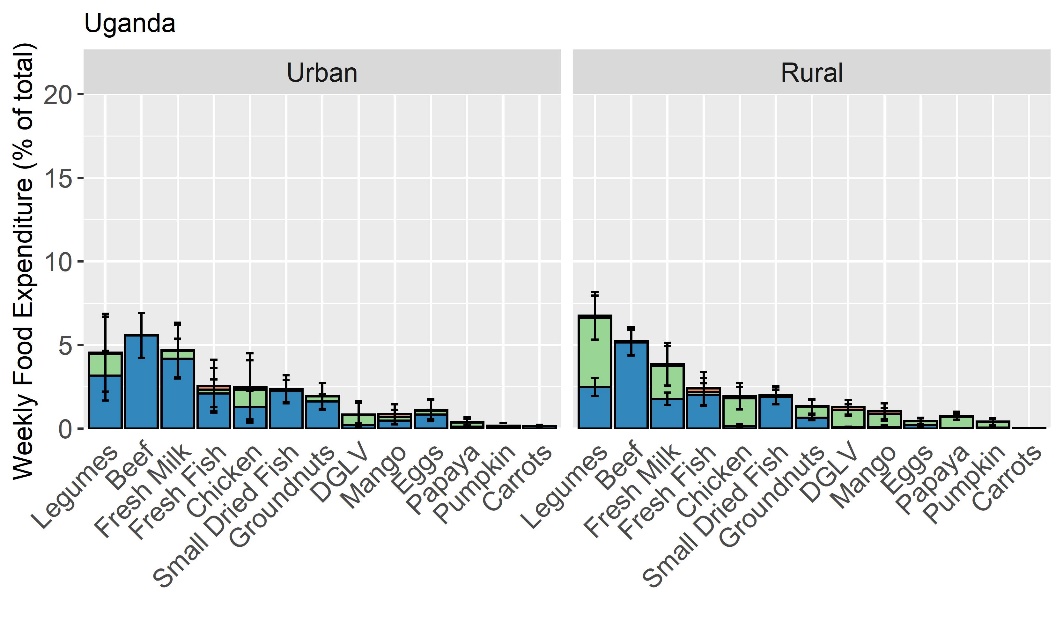

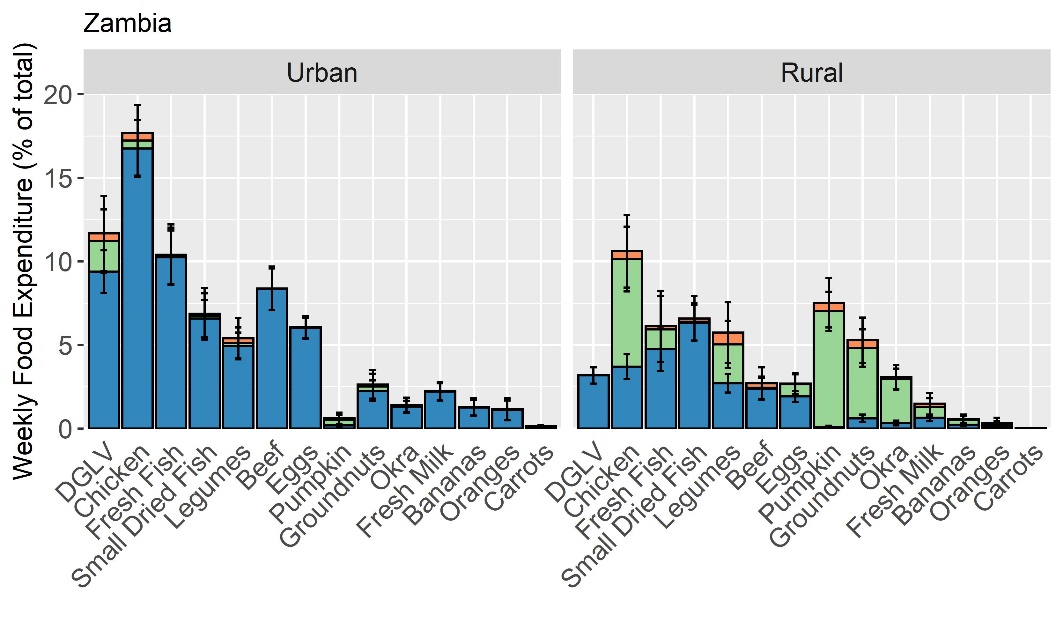


Note: only households with children of complementary feeding age are shown. Breakdowns of food expenditure by type were not available for South Africa. The Mozambique survey only covered expenditures from purchases and own production, not other sources.

# Figure S13: Current consumption of selected nutritious foods, by quintile


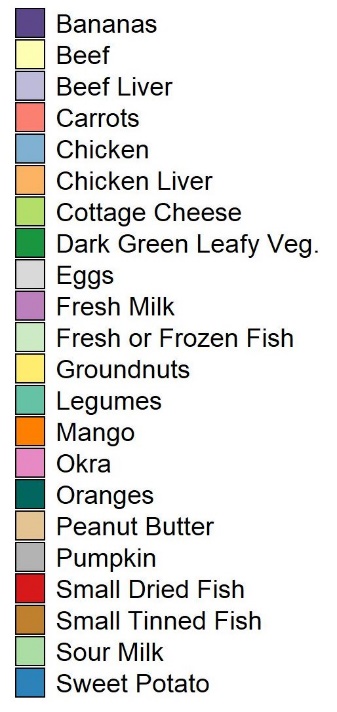

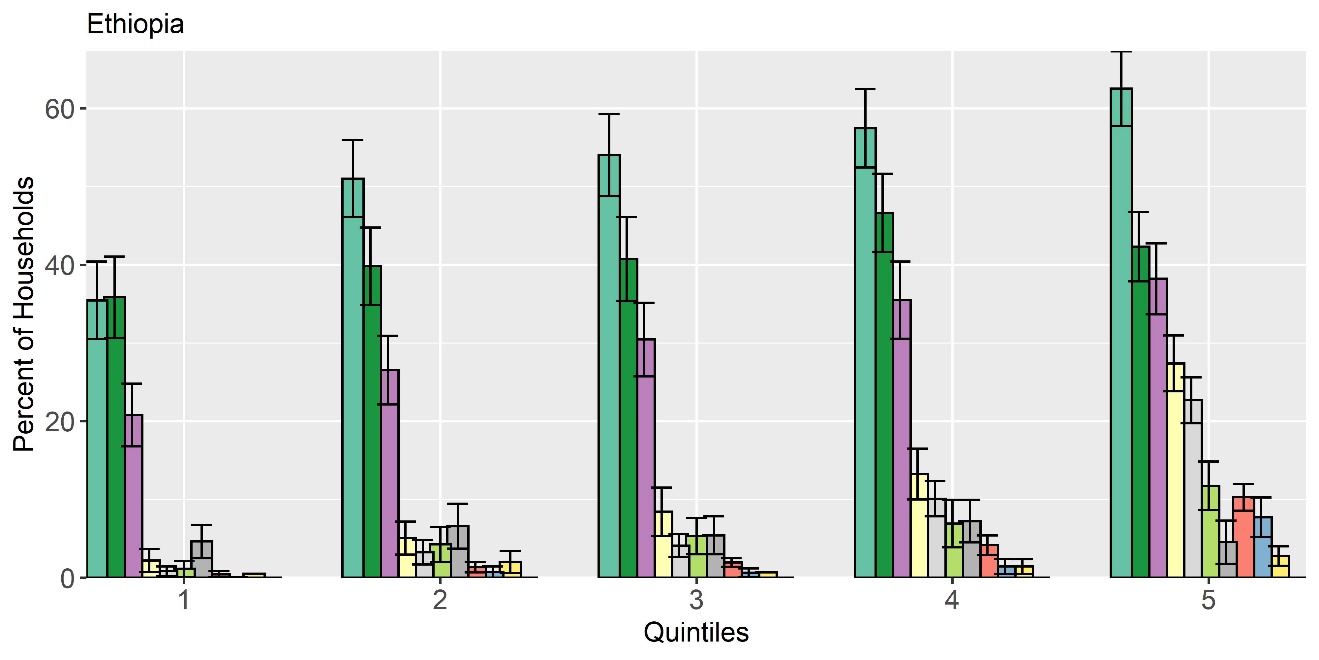

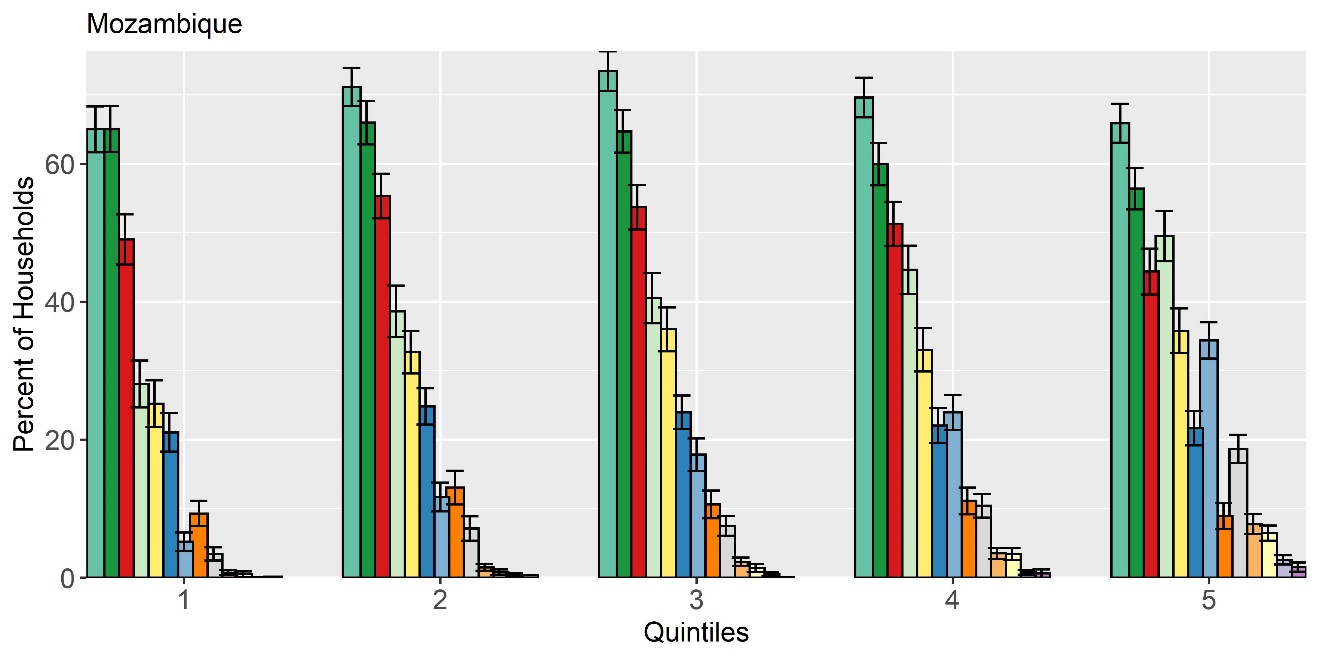

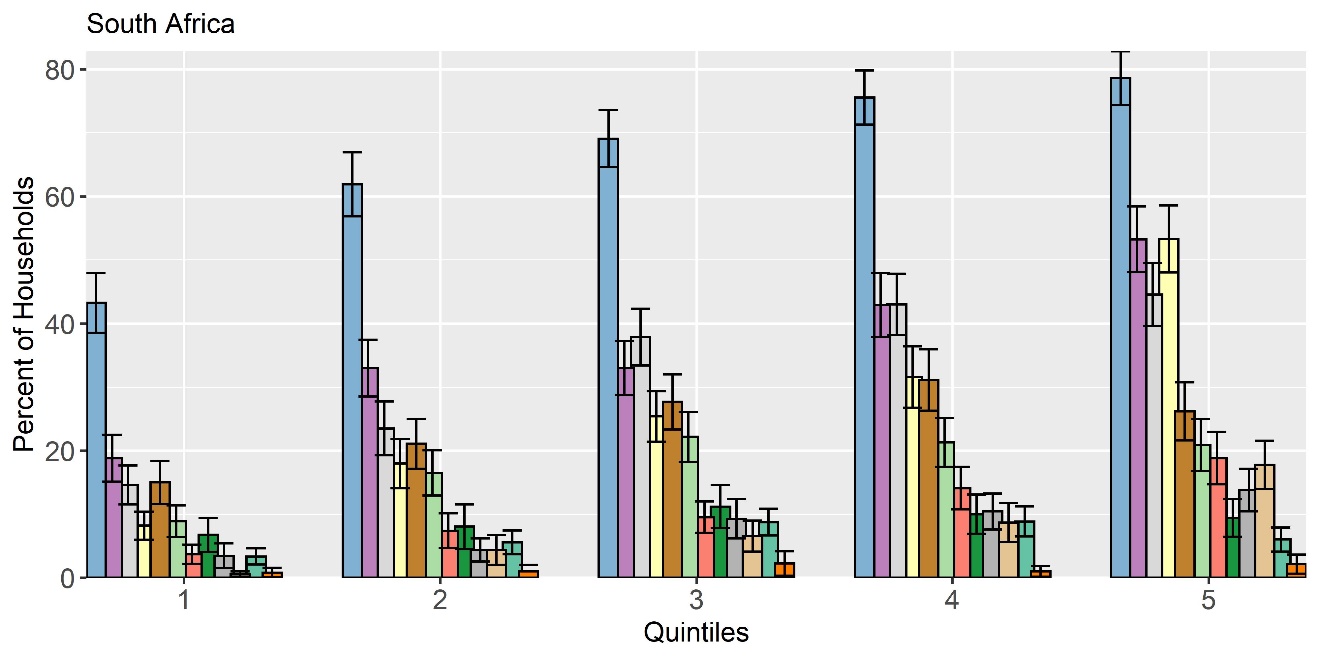

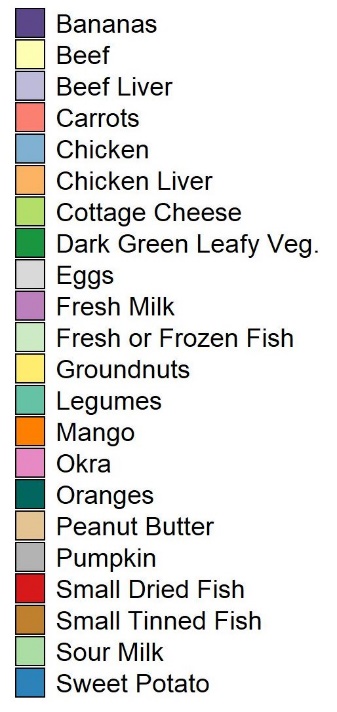

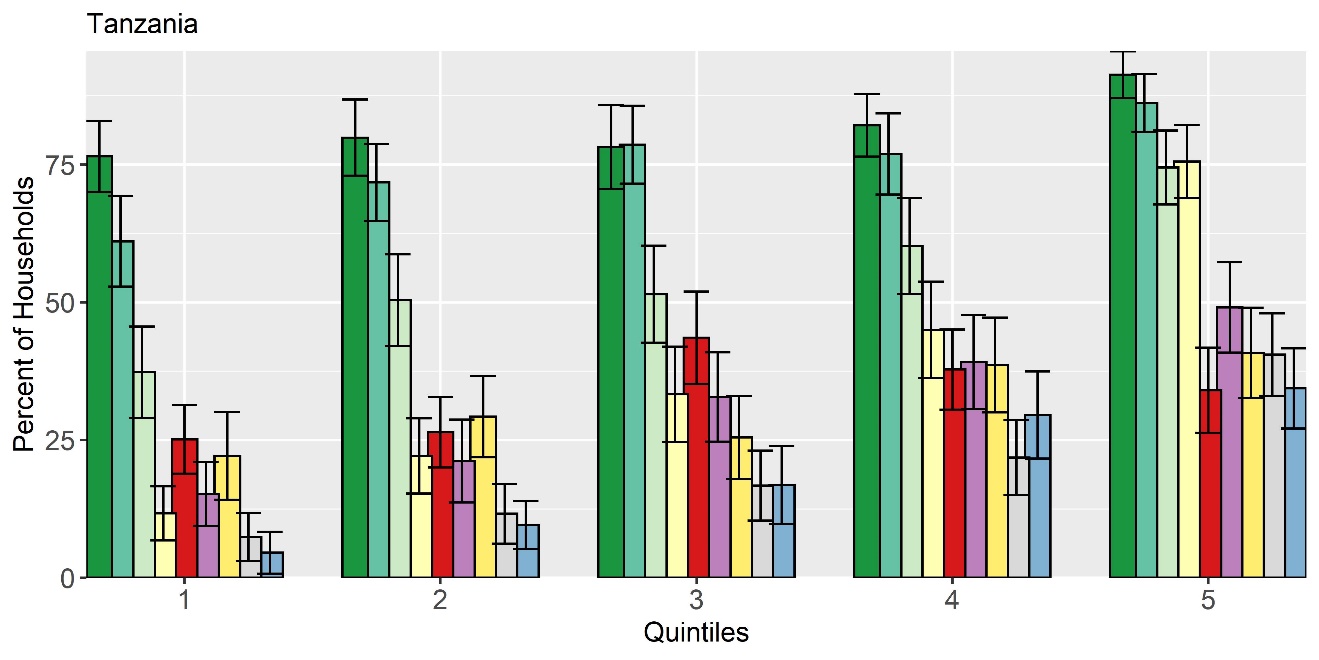

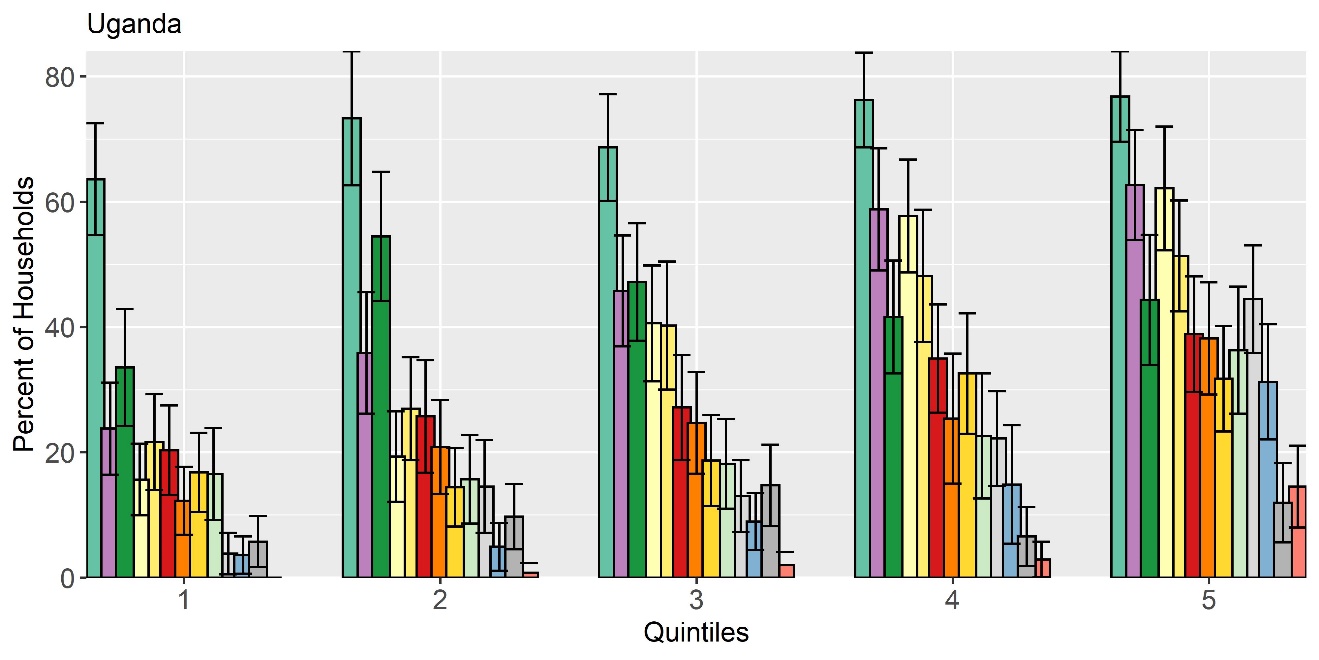

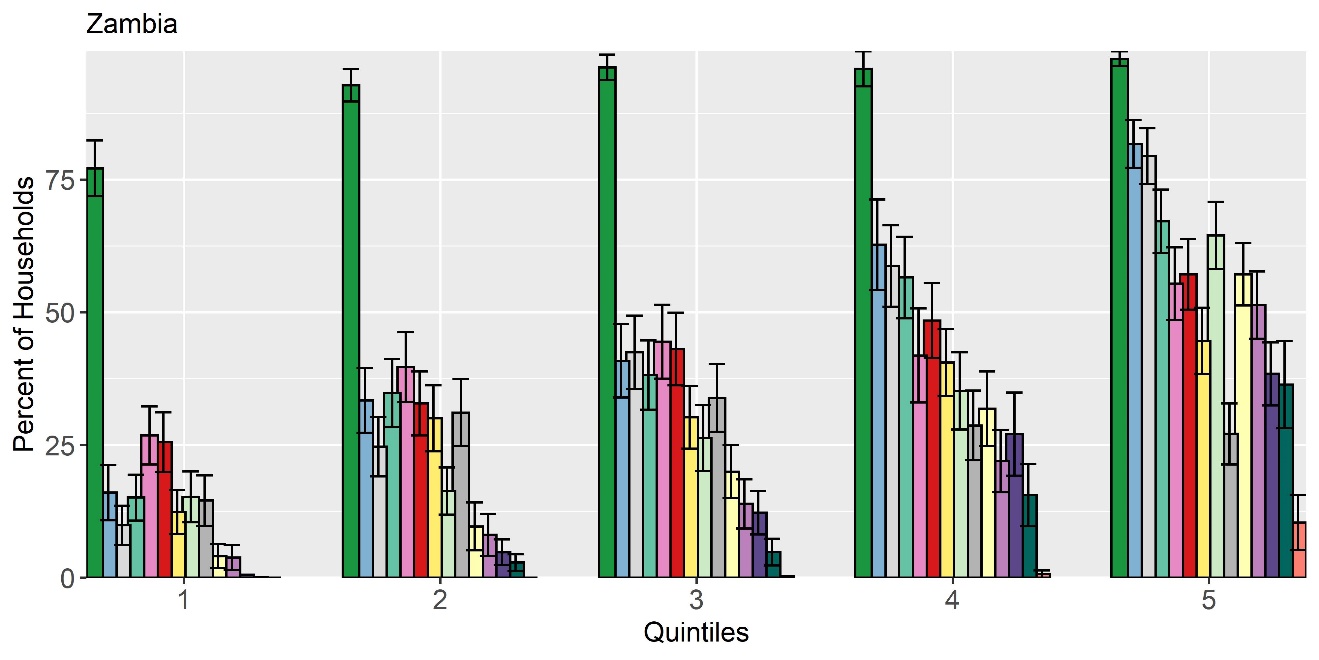


Note: only households with children of complementary feeding age are shown.

# Figure S14: Current expenditure on selected nutritious foods, by quintile


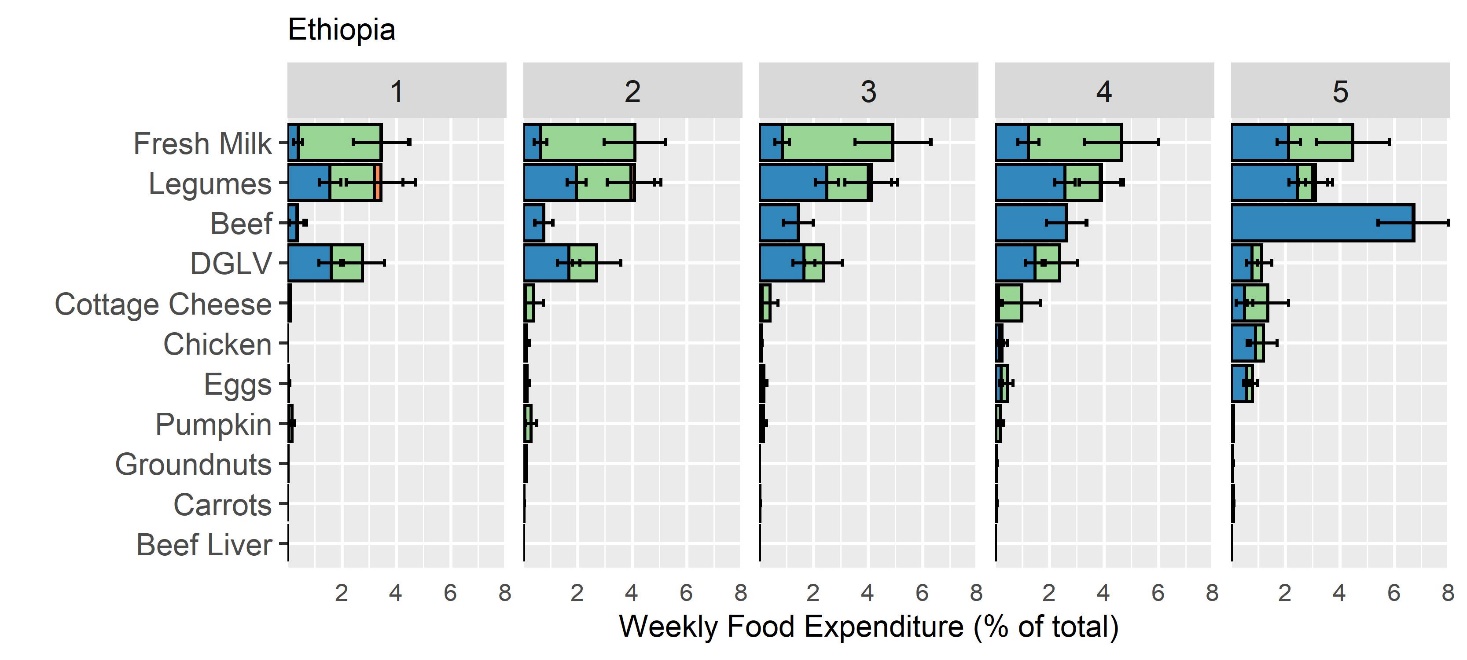

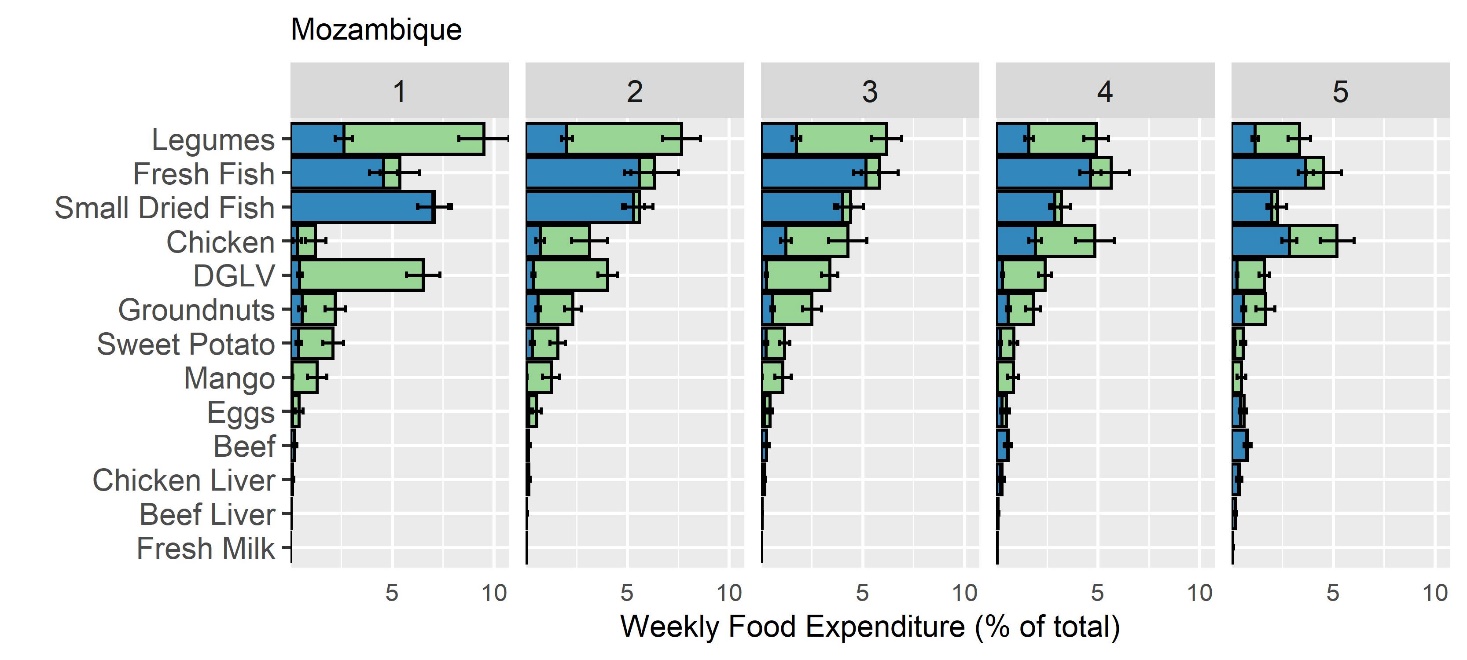

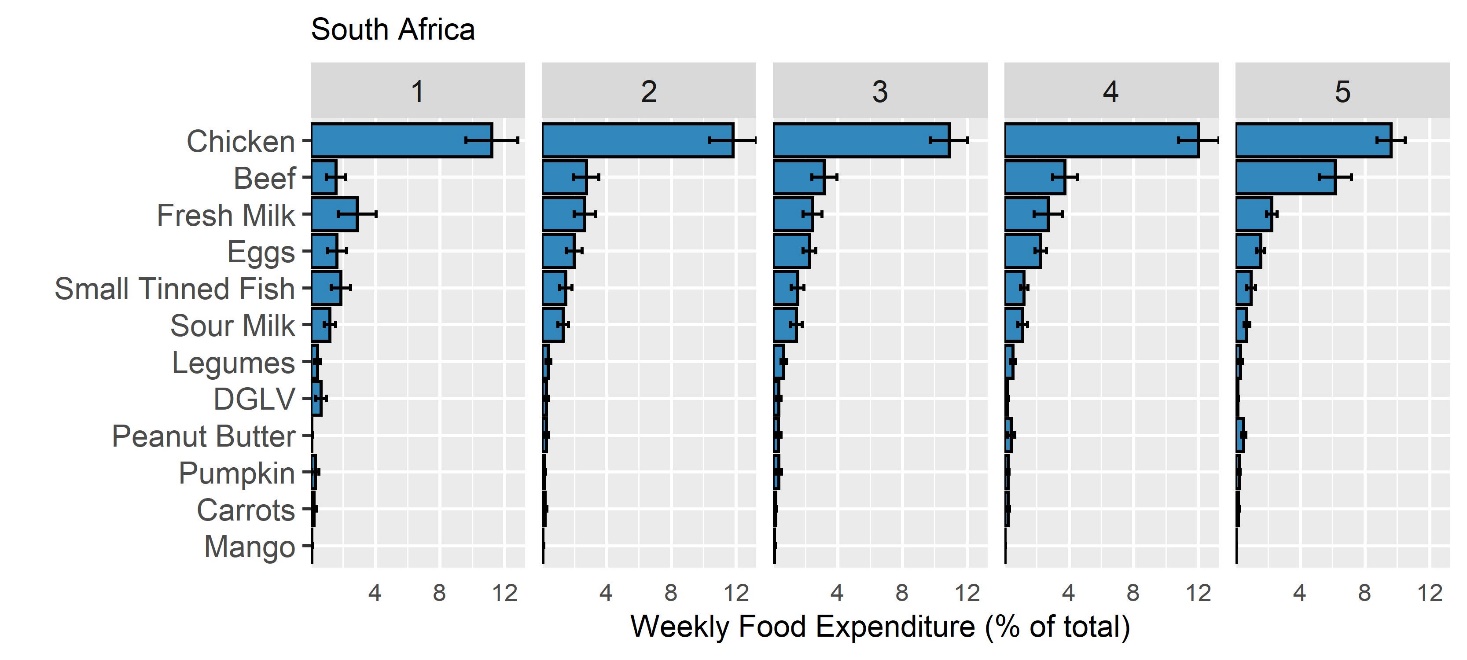

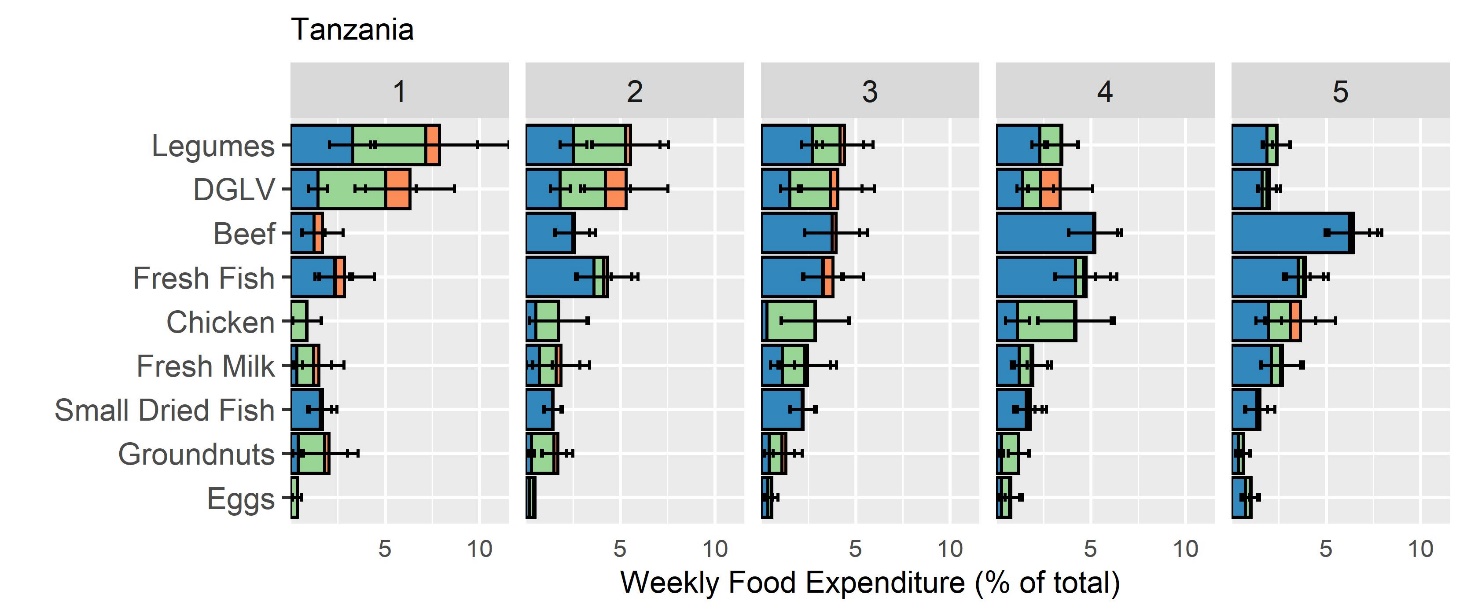

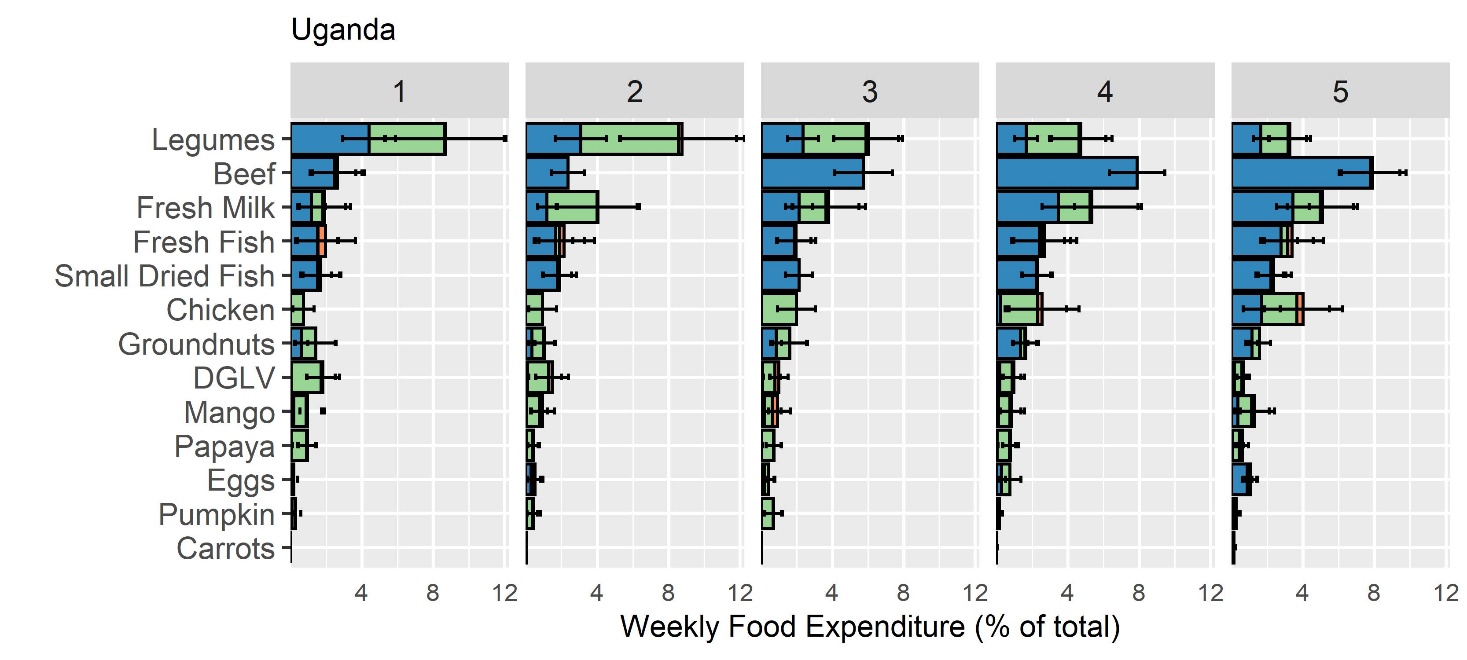

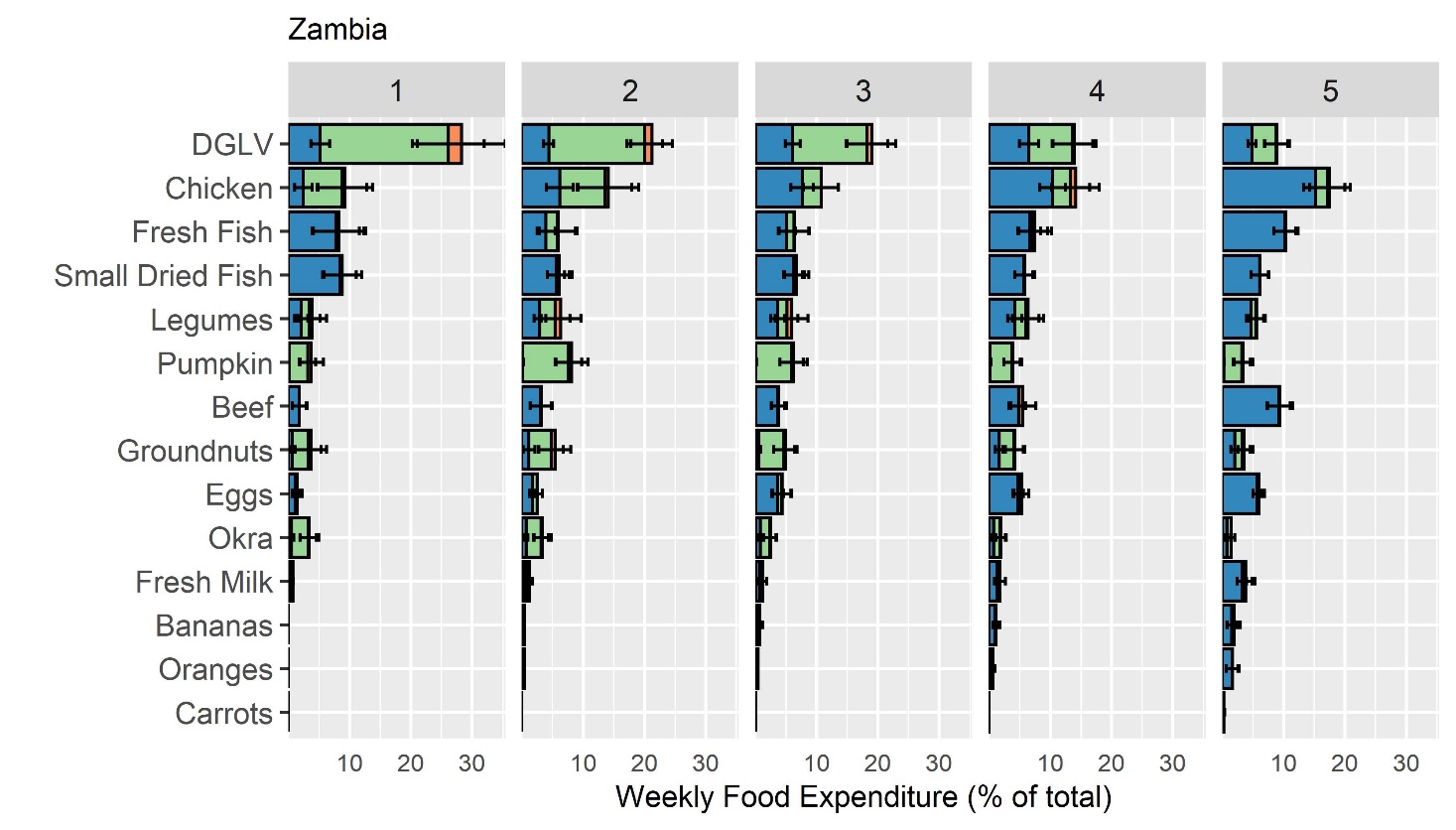
Note: only households with children of complementary feeding age are shown. Breakdowns of food expenditure by type were not available for South Africa. The Mozambique data only covered expenditures from purchases and own production, not other sources.

# Figure S15: **Portion size cost, as a share of total household food expenditure per adult equivalent, by food**


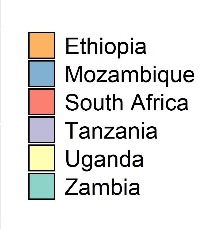

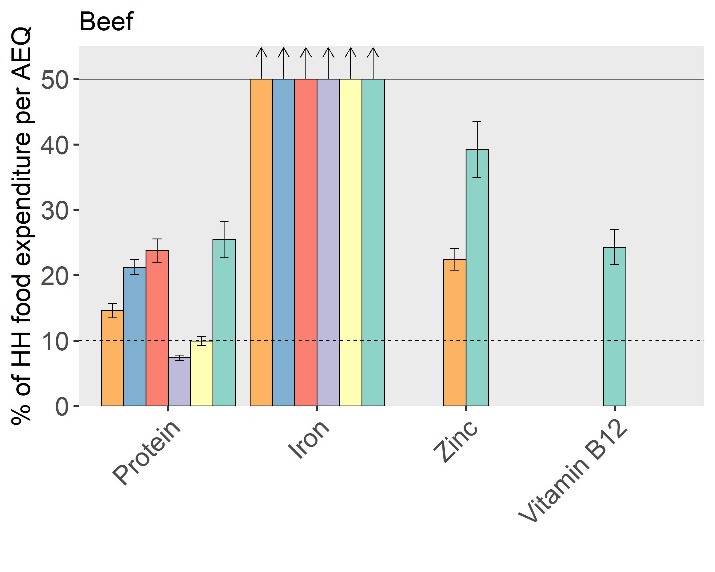

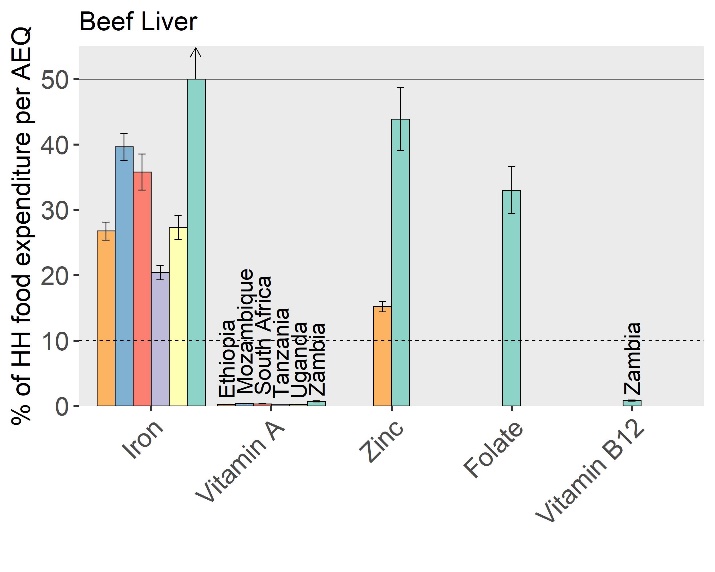

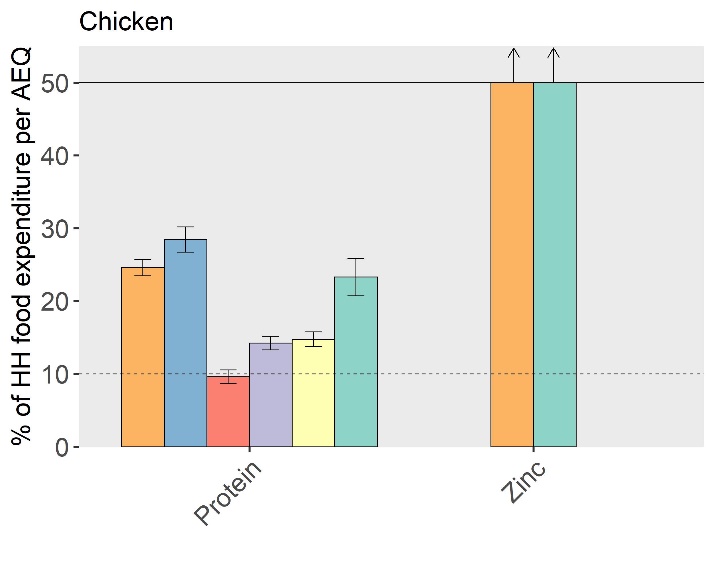

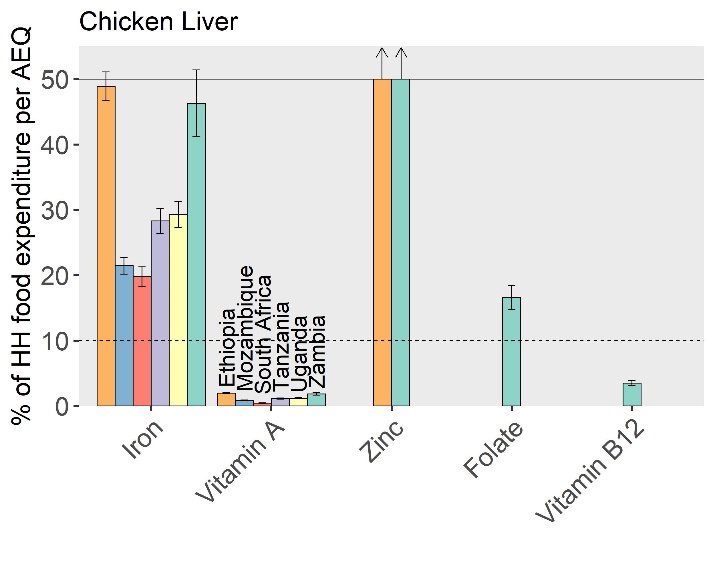

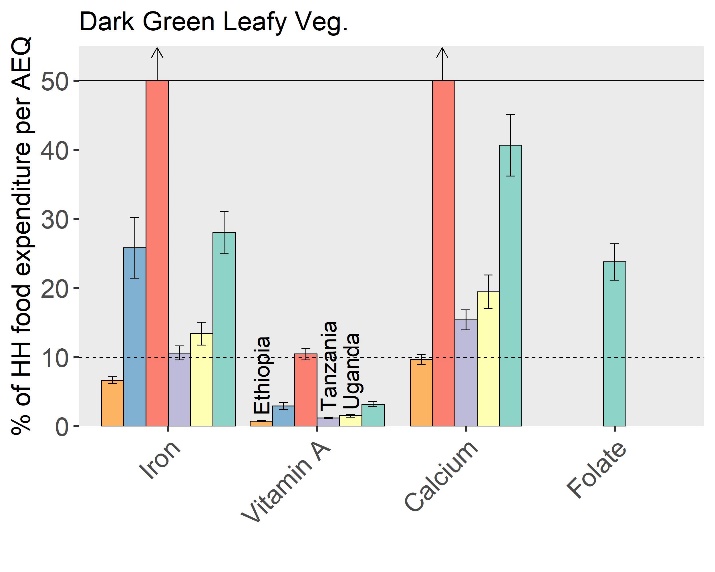

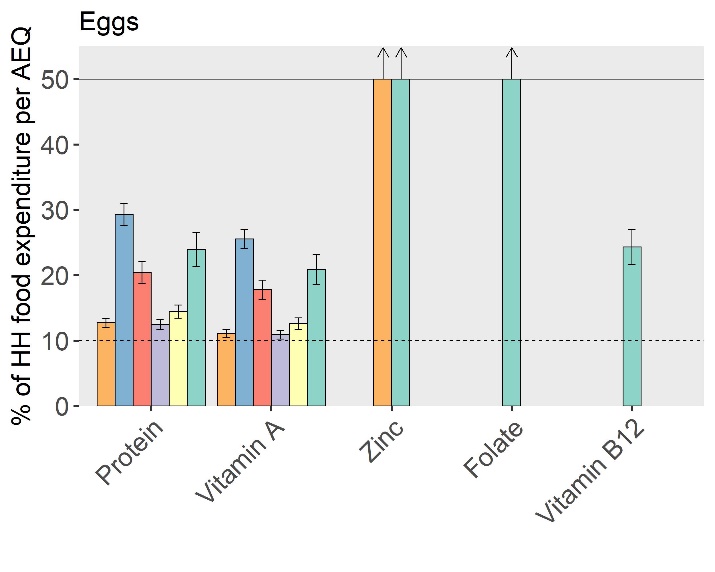

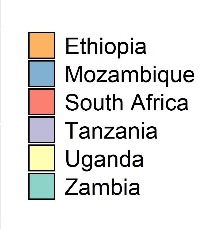

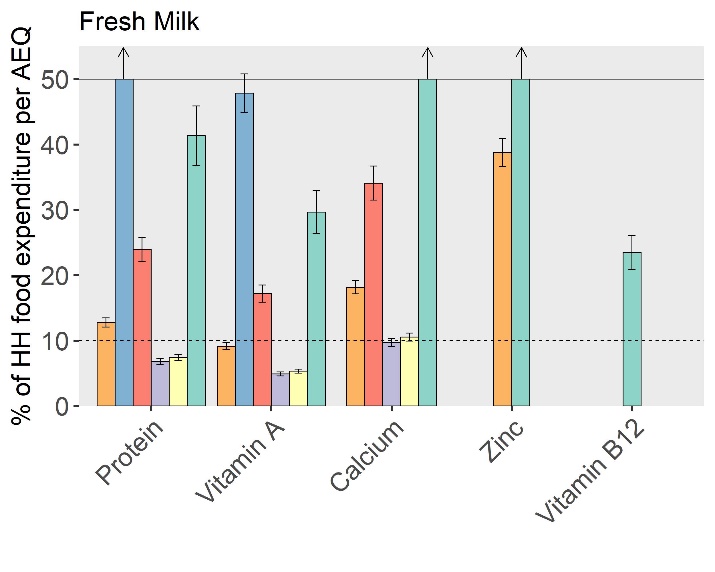

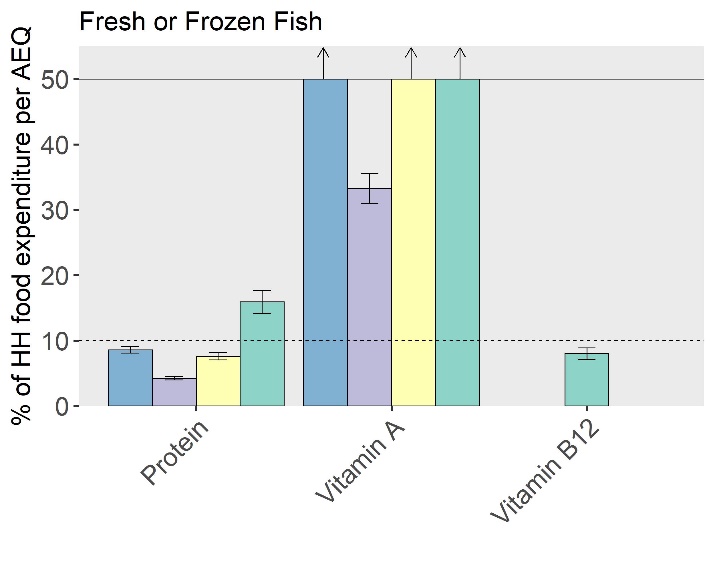

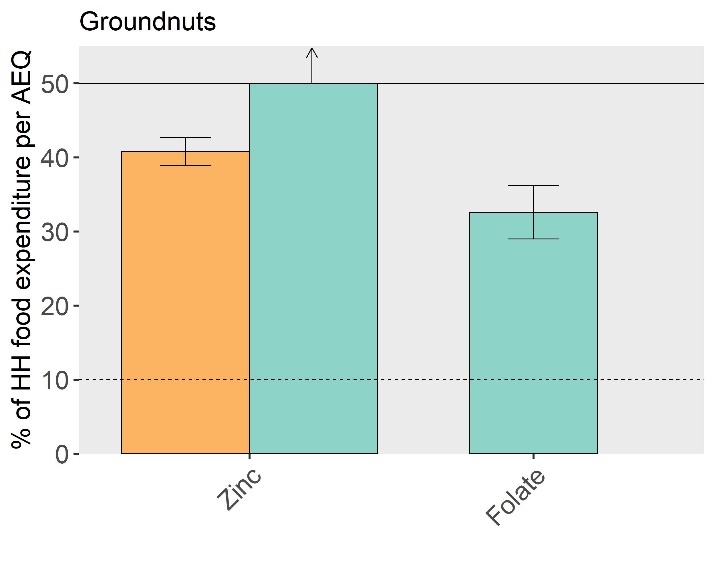

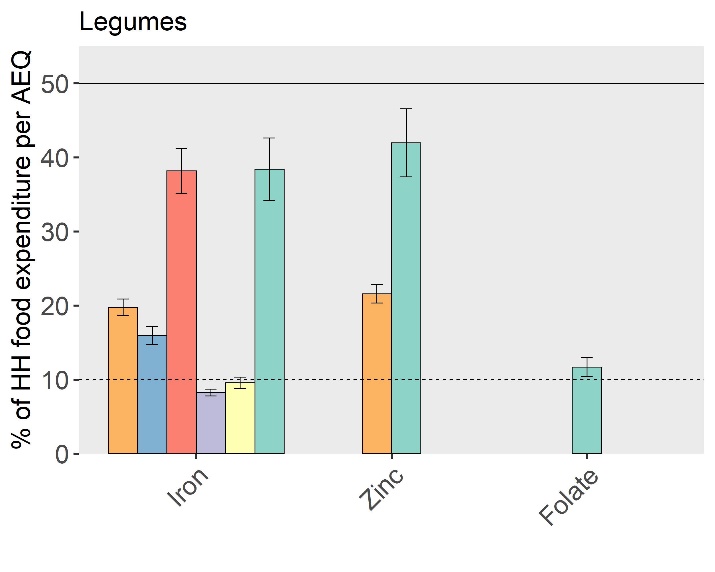

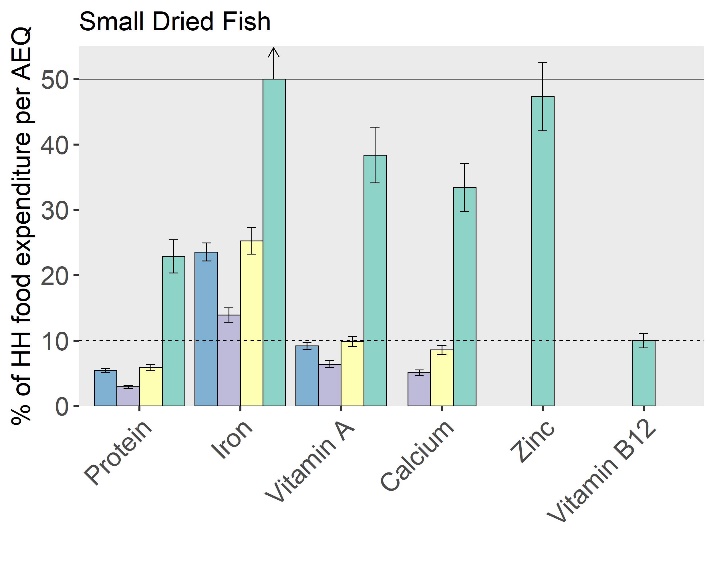

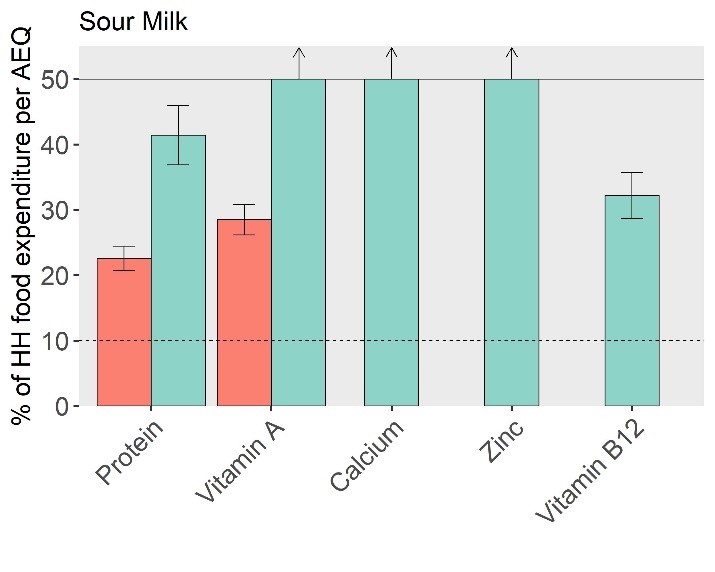


Note: the figure includes only foods that could meet multiple nutrient needs for multiple countries. The y-axis was truncated at 55%, but the cost of some foods exceeded 55% of household food expenditure per AEQ. Regional price data were not available for Zambia and thus the confidence intervals shown on the Zambia panels do not incorporate geographic price variation.

# Figure S16: Portion size cost, as a share of total household food expenditure per adult equivalent, by rural/urban setting


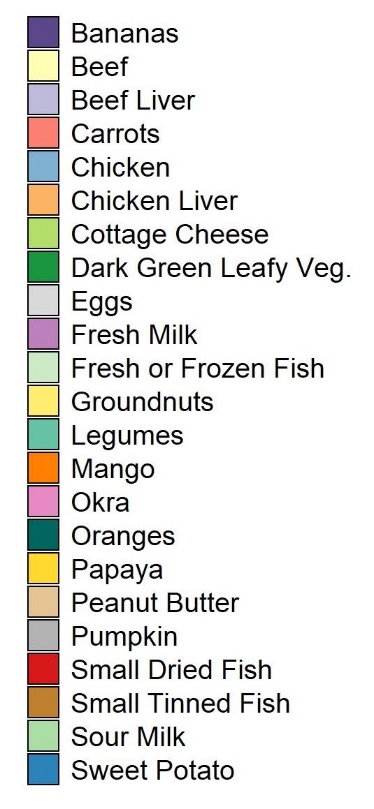

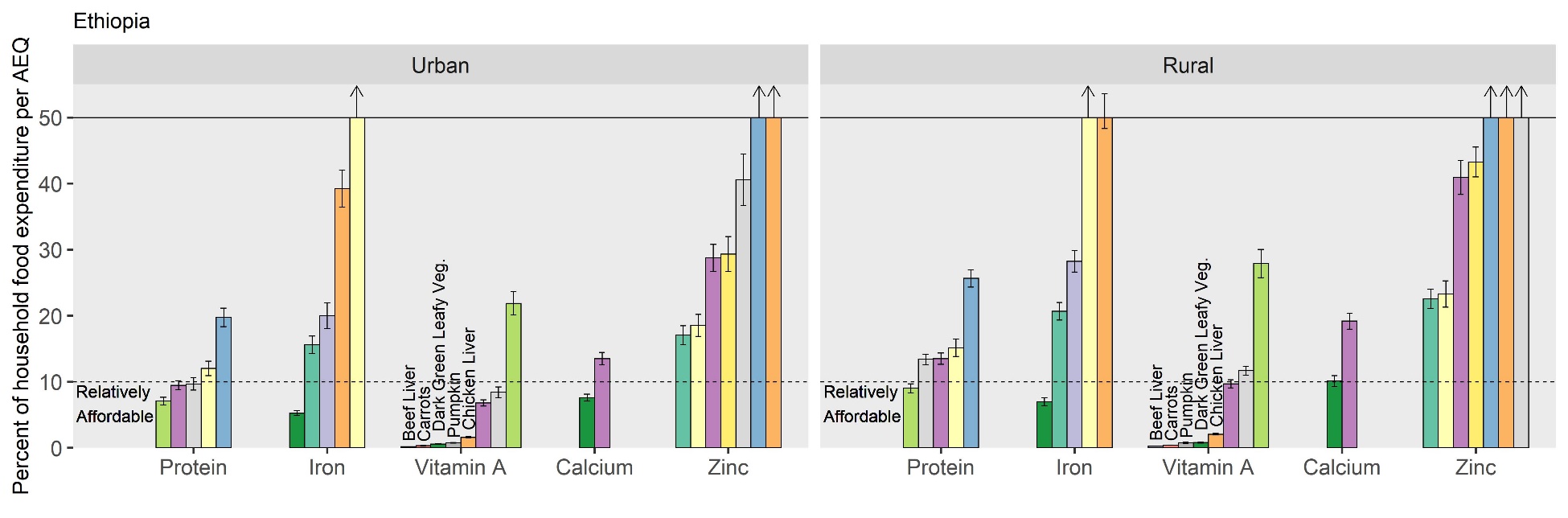

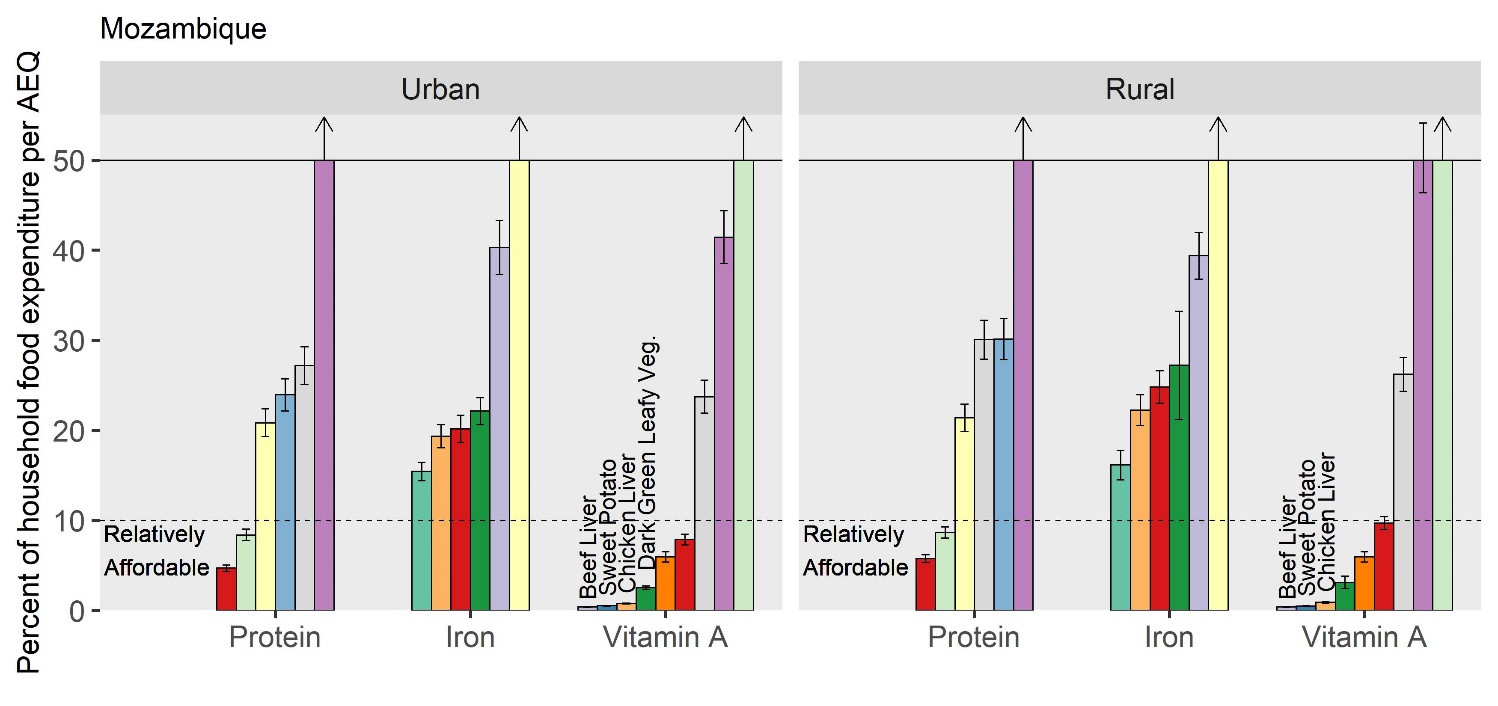


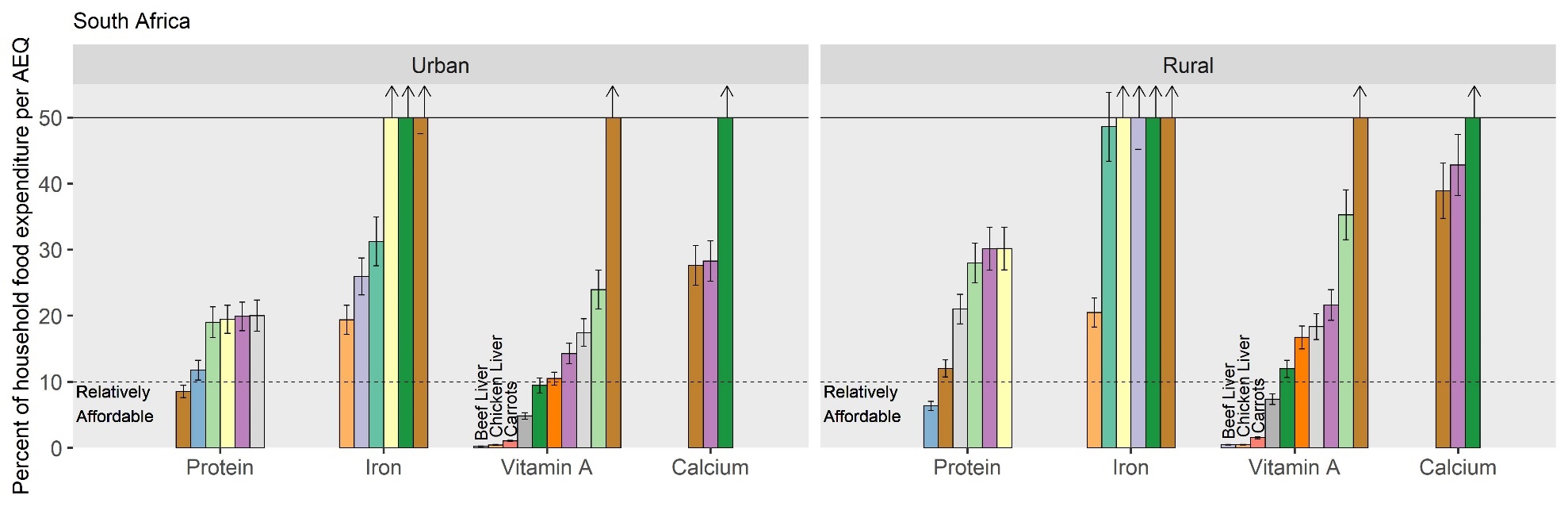

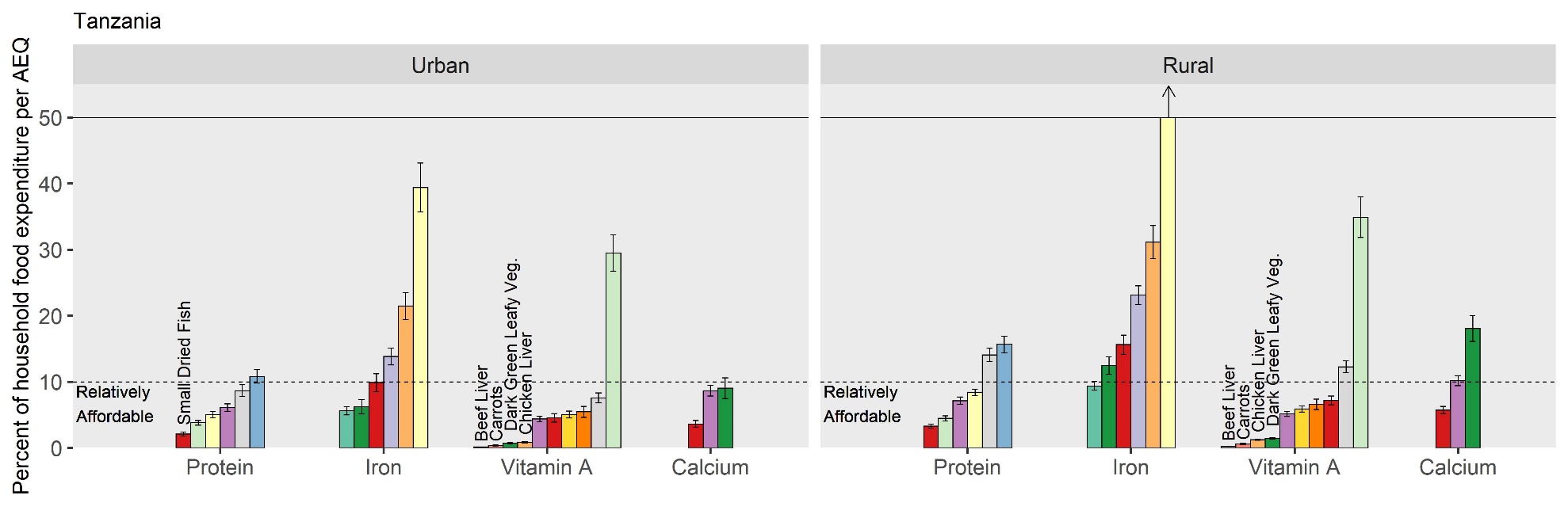


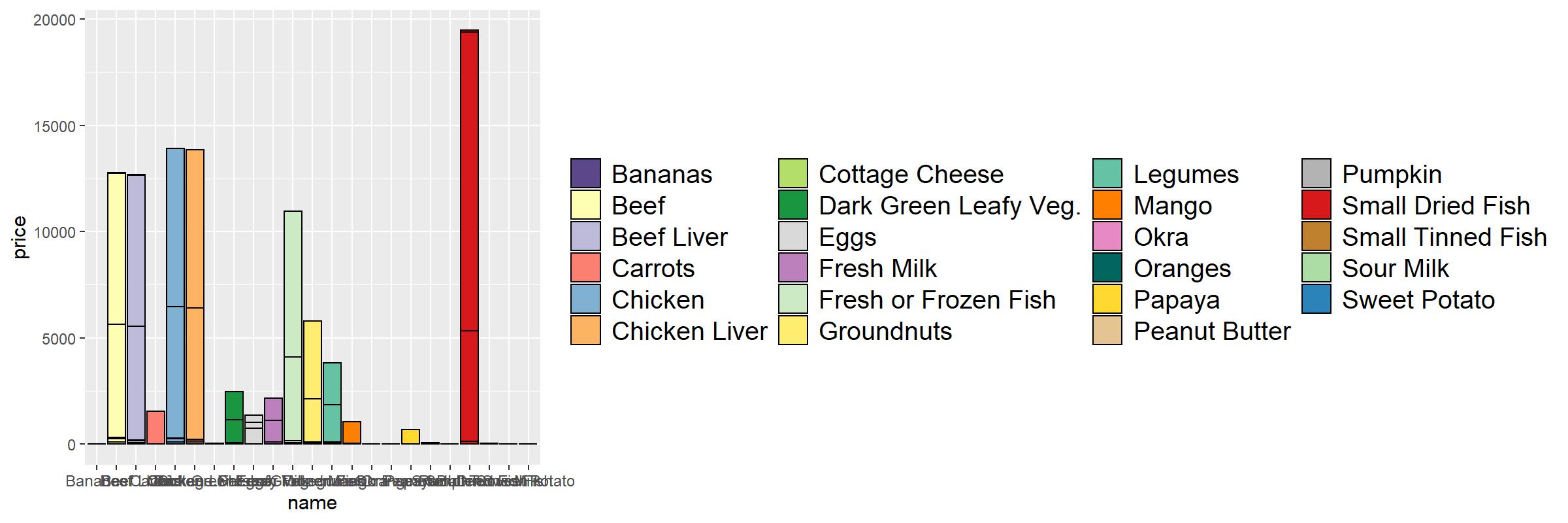

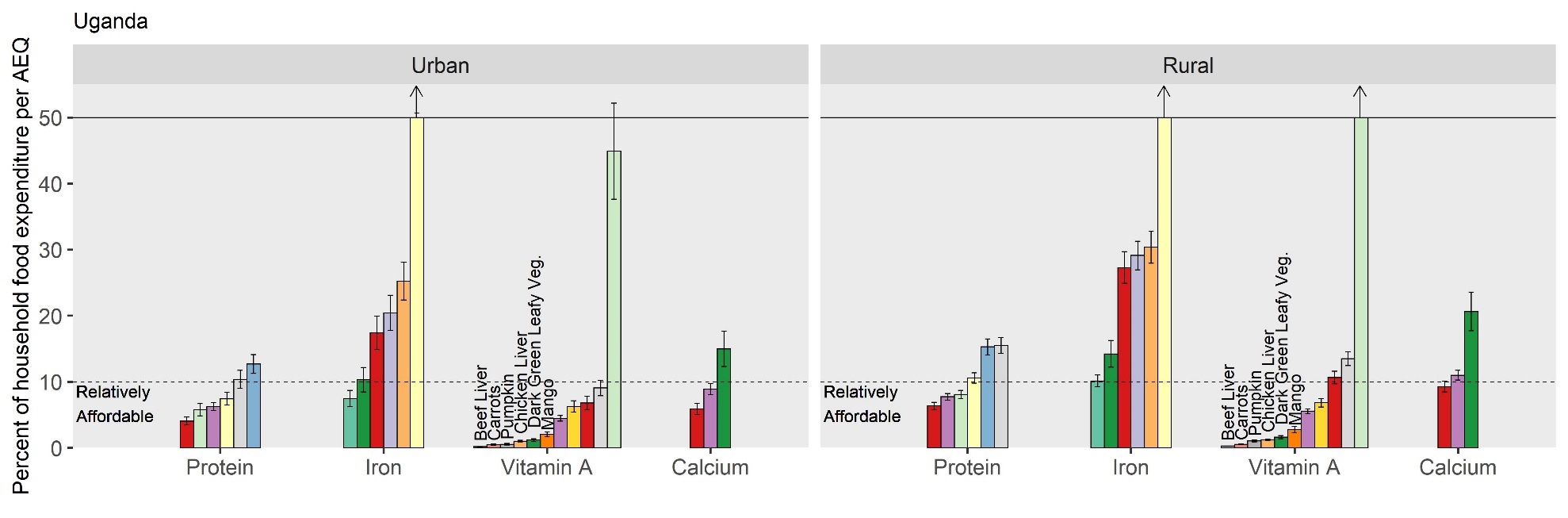


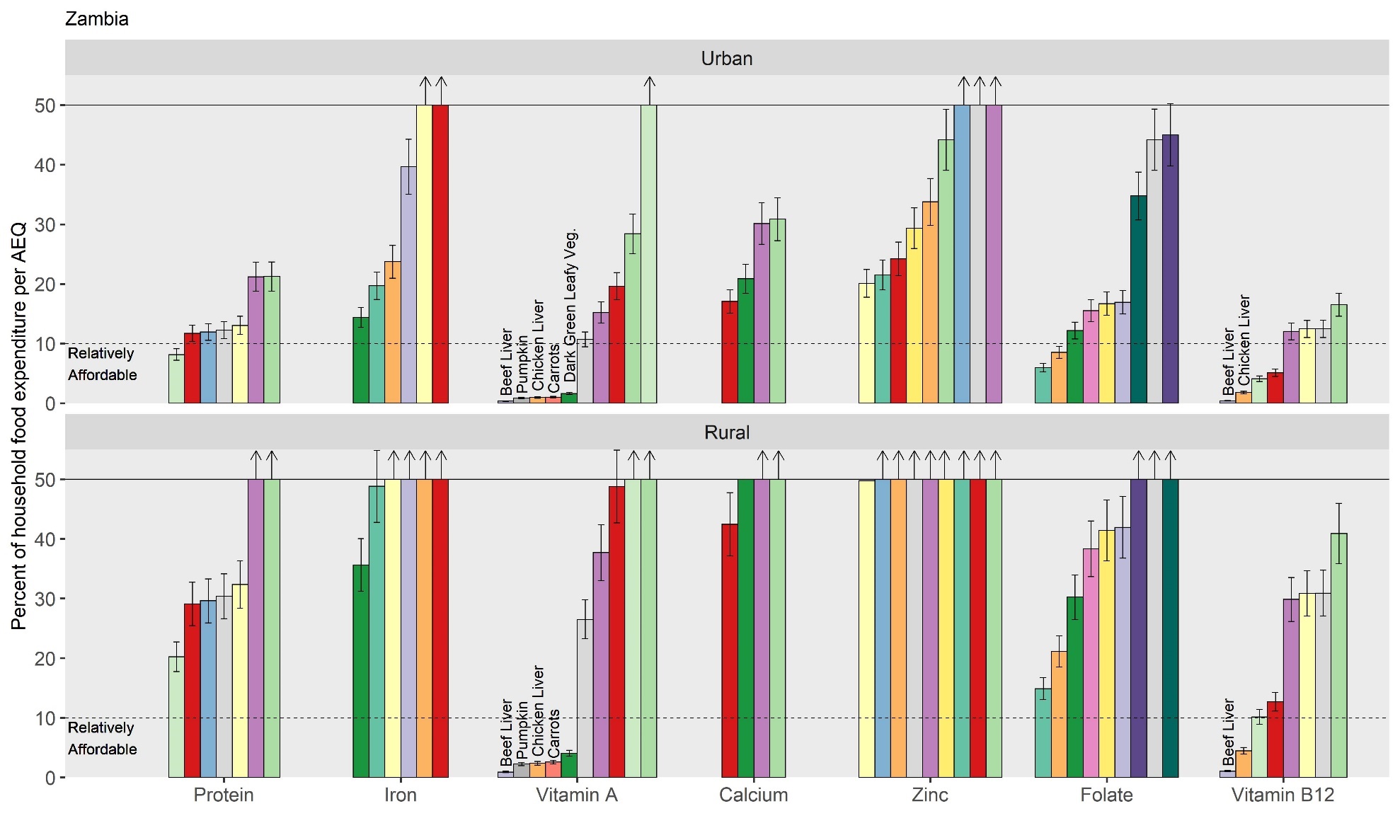


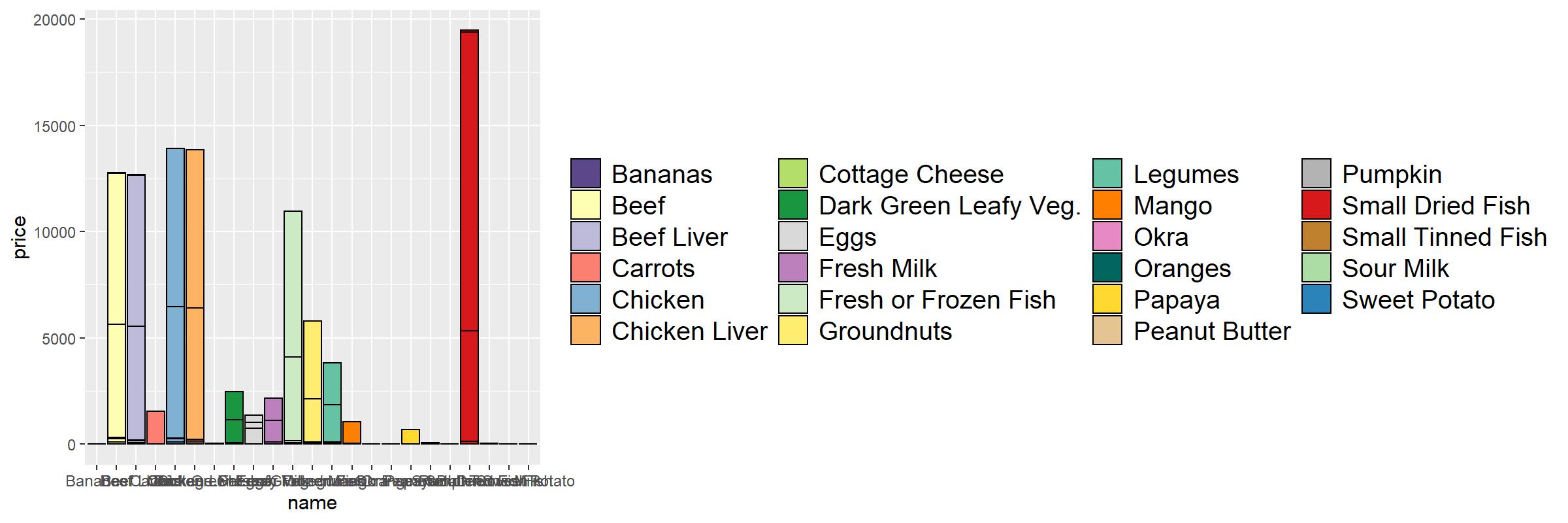

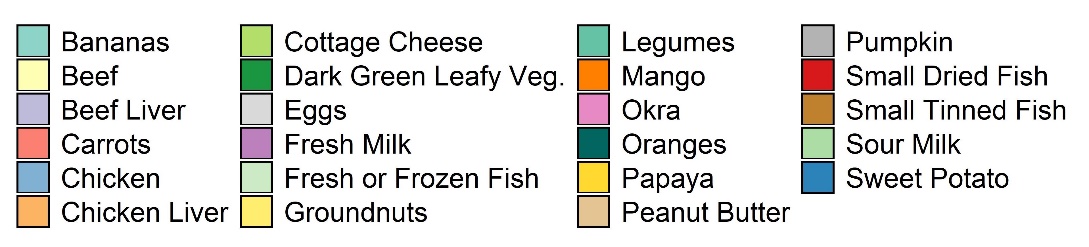
Note: The y-axis was truncated at 50, but the costs of some foods exceeded 50% of household food expenditure per AEQ; these foods are designated with vertical arrows indicating that the bar continues vertically beyond the scale of the graph. Regional price data were not available for Zambia and thus the confidence intervals shown on the Zambia panels do not incorporate geographic price variation.

# Figure S17: Food cost per kcal by rural/urban setting


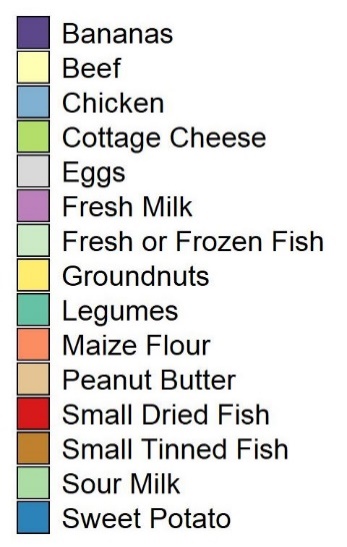

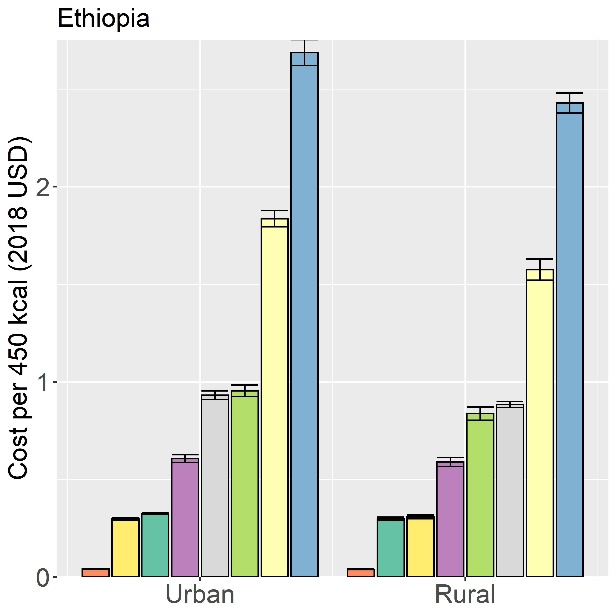

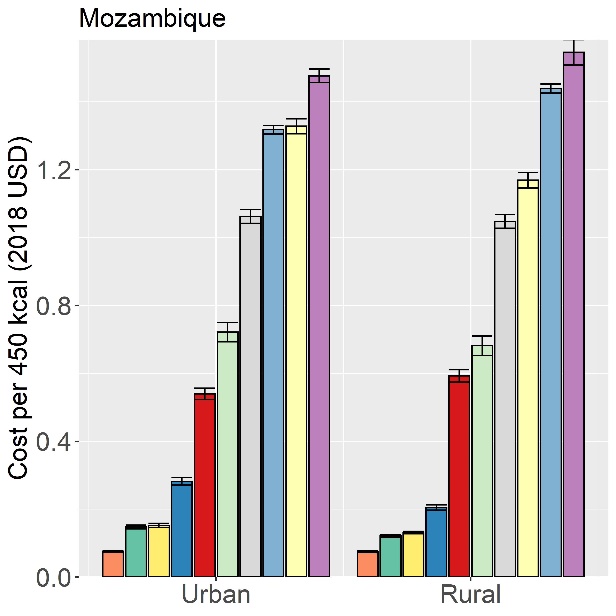

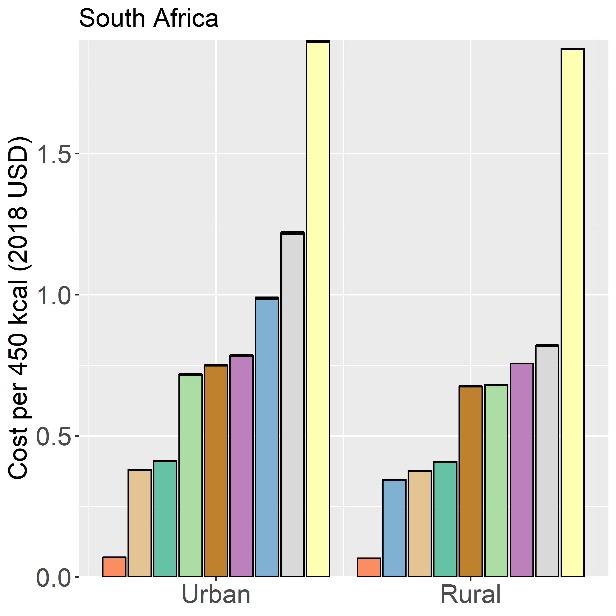

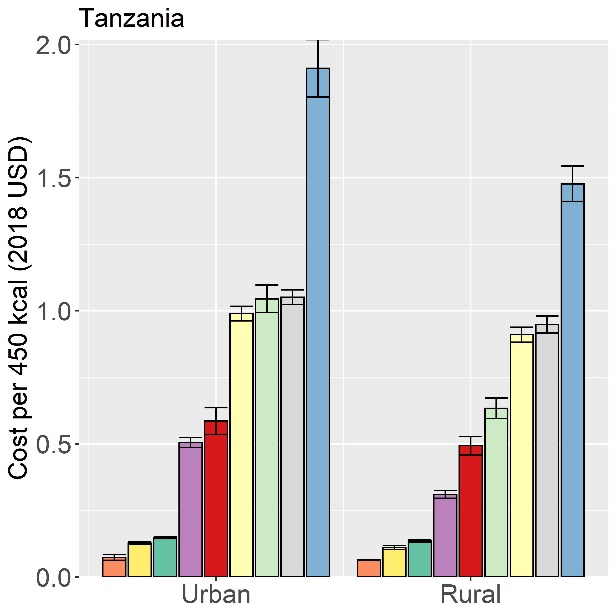

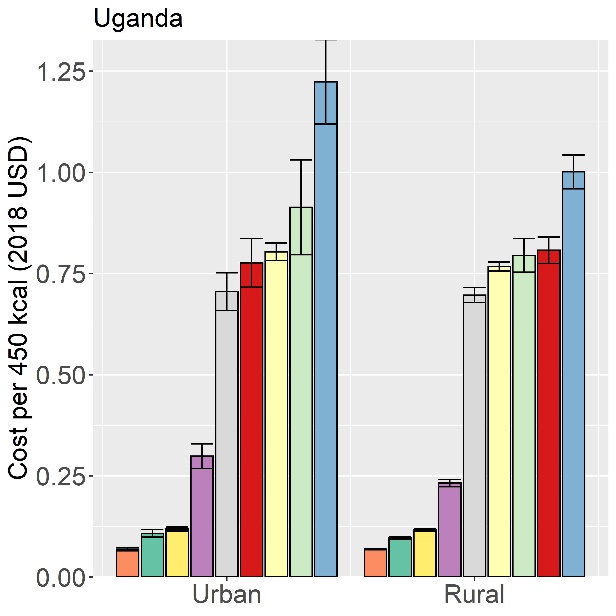

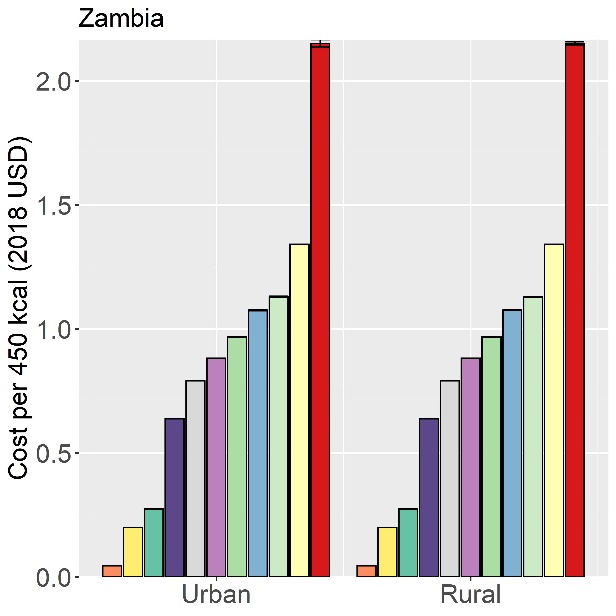


Note: Regional price data were not available for Zambia and thus the confidence intervals shown on the Zambia panels do not

Figure S18: Average share of micronutrient requirements affordability analysis, by rural/urban setting


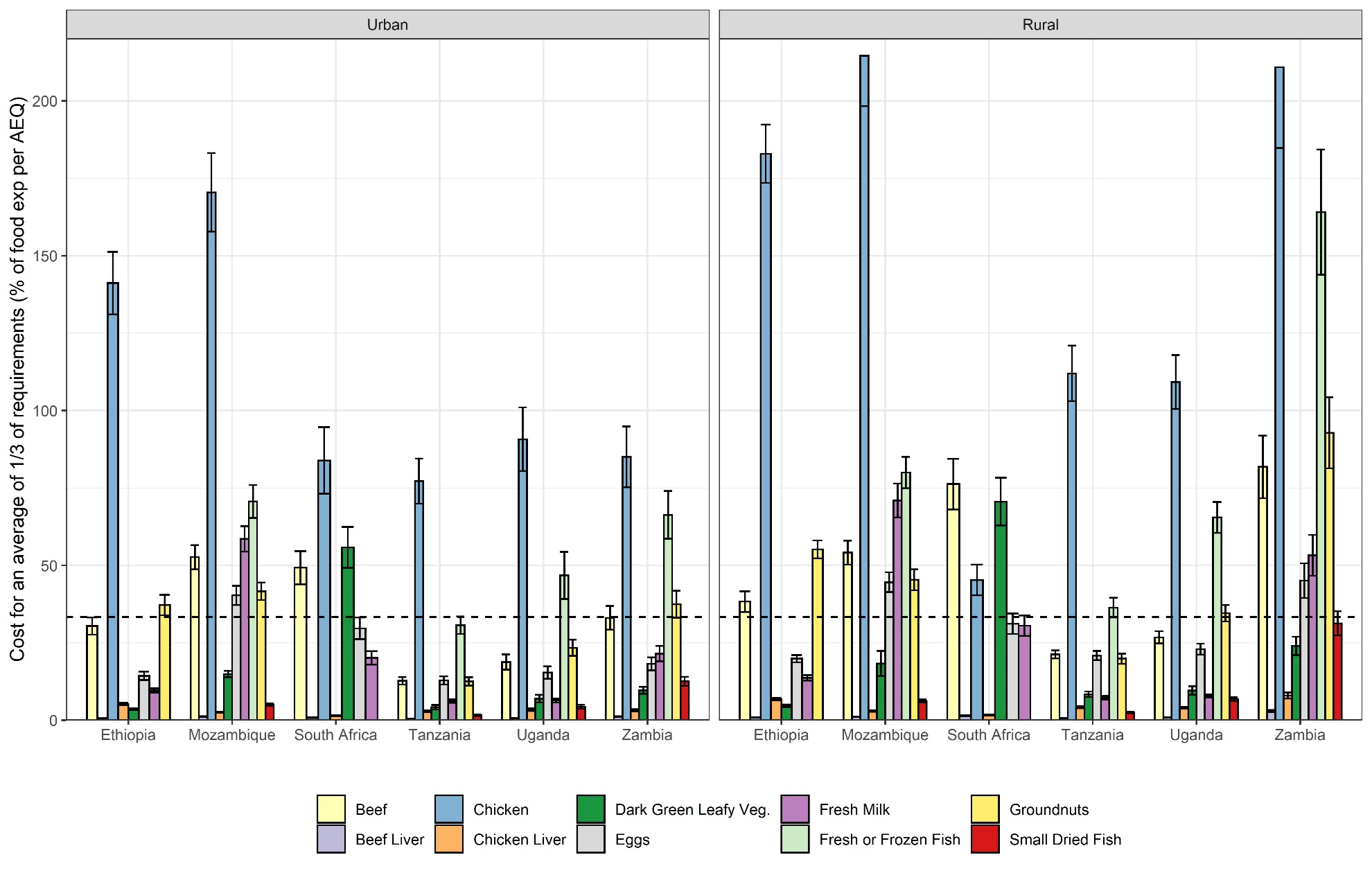


Note: Regional price data were not available for Zambia and thus the confidence intervals shown for Zambia do not incorporate geographic price variation.

Figure S19: Portion size cost, as a share of total household food expenditure per adult equivalent, by quintile


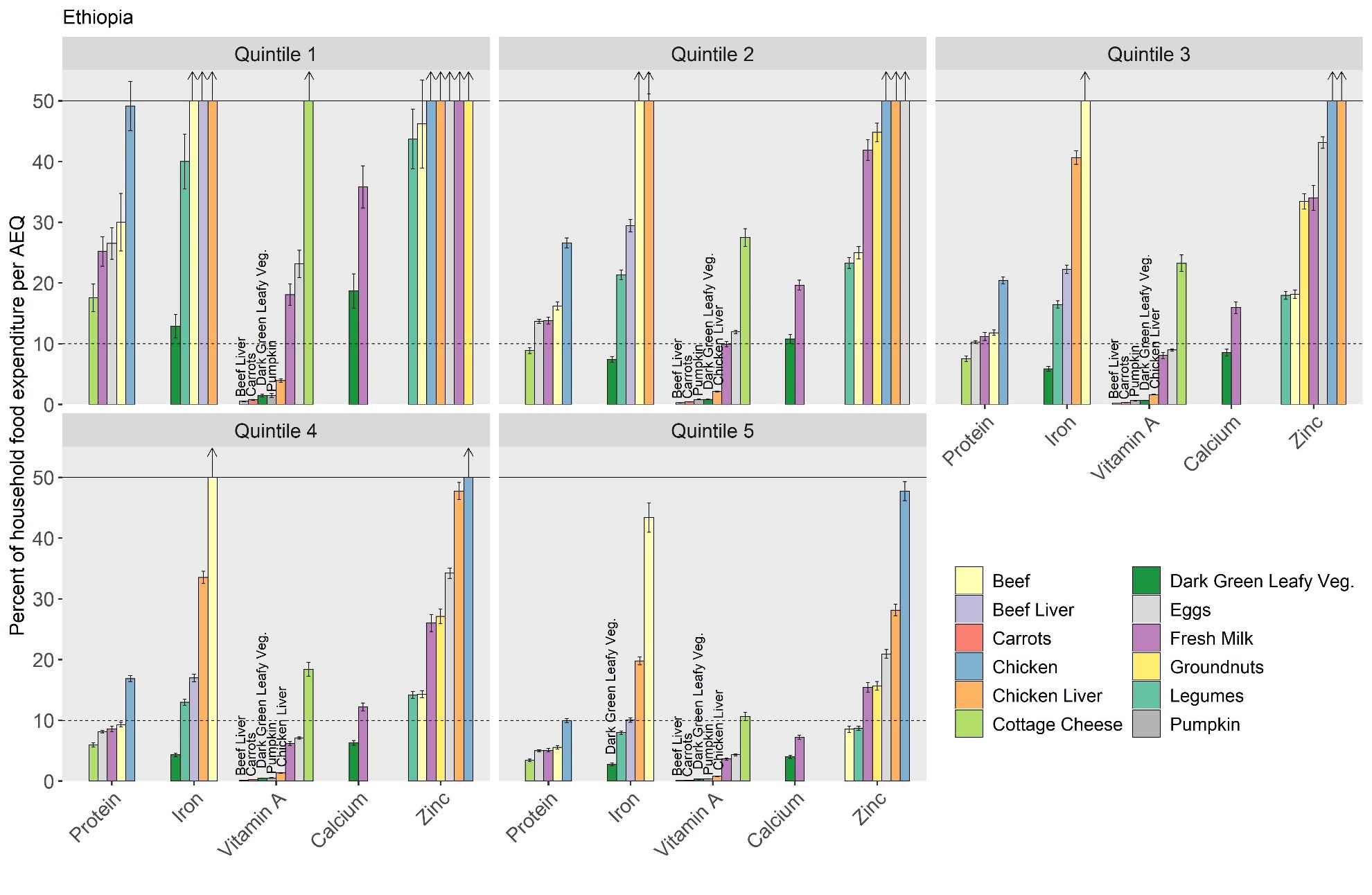


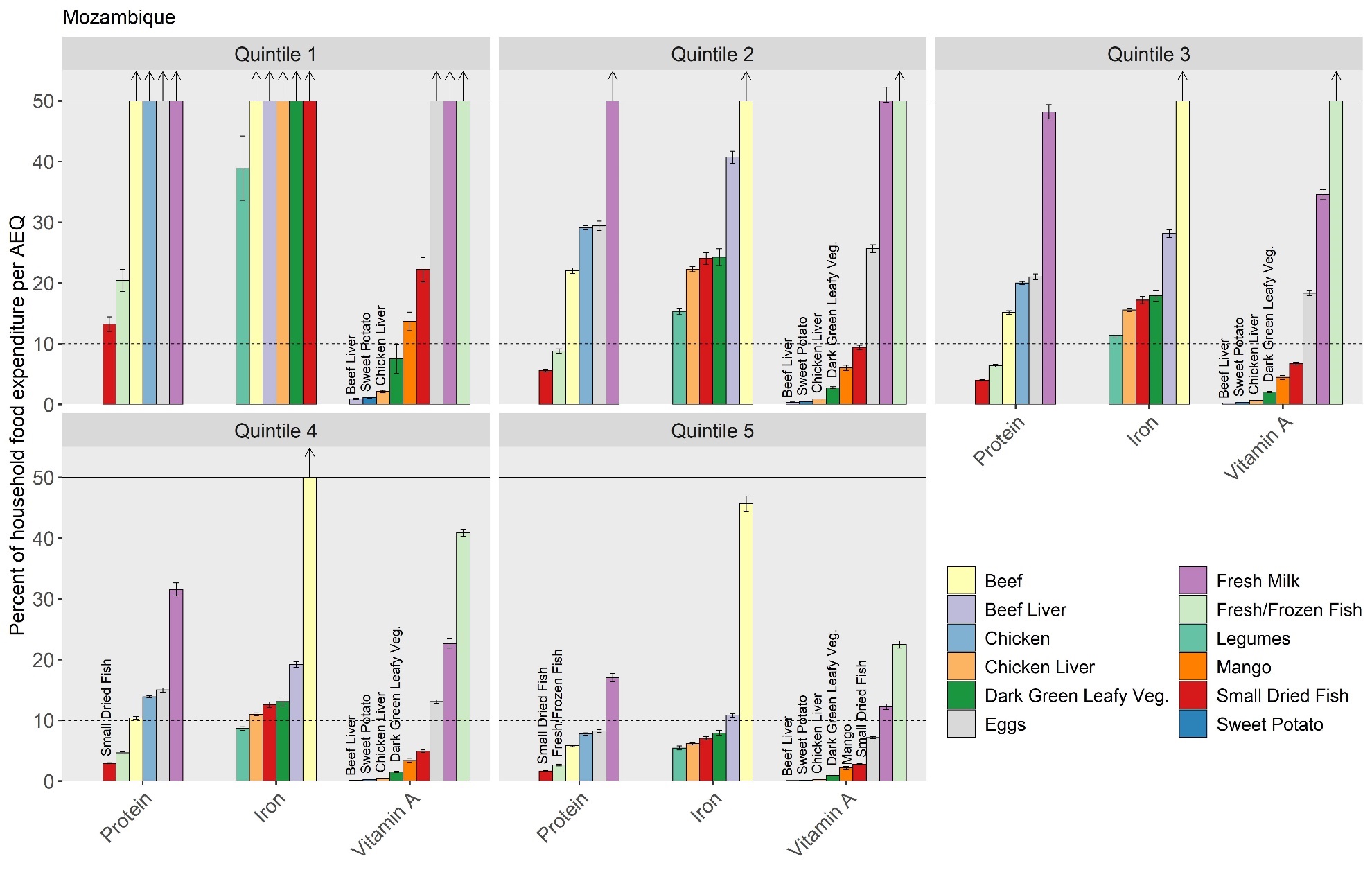

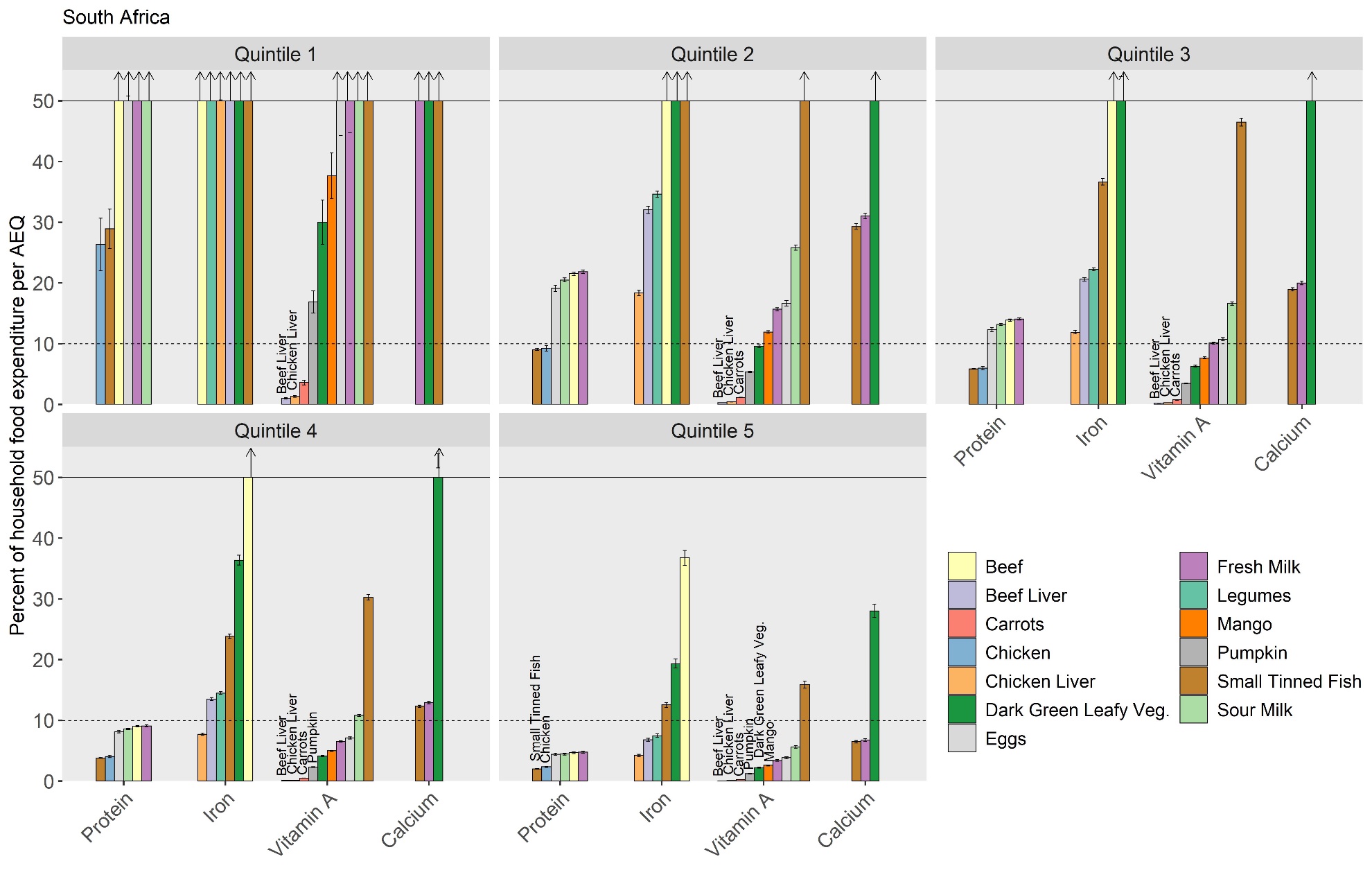

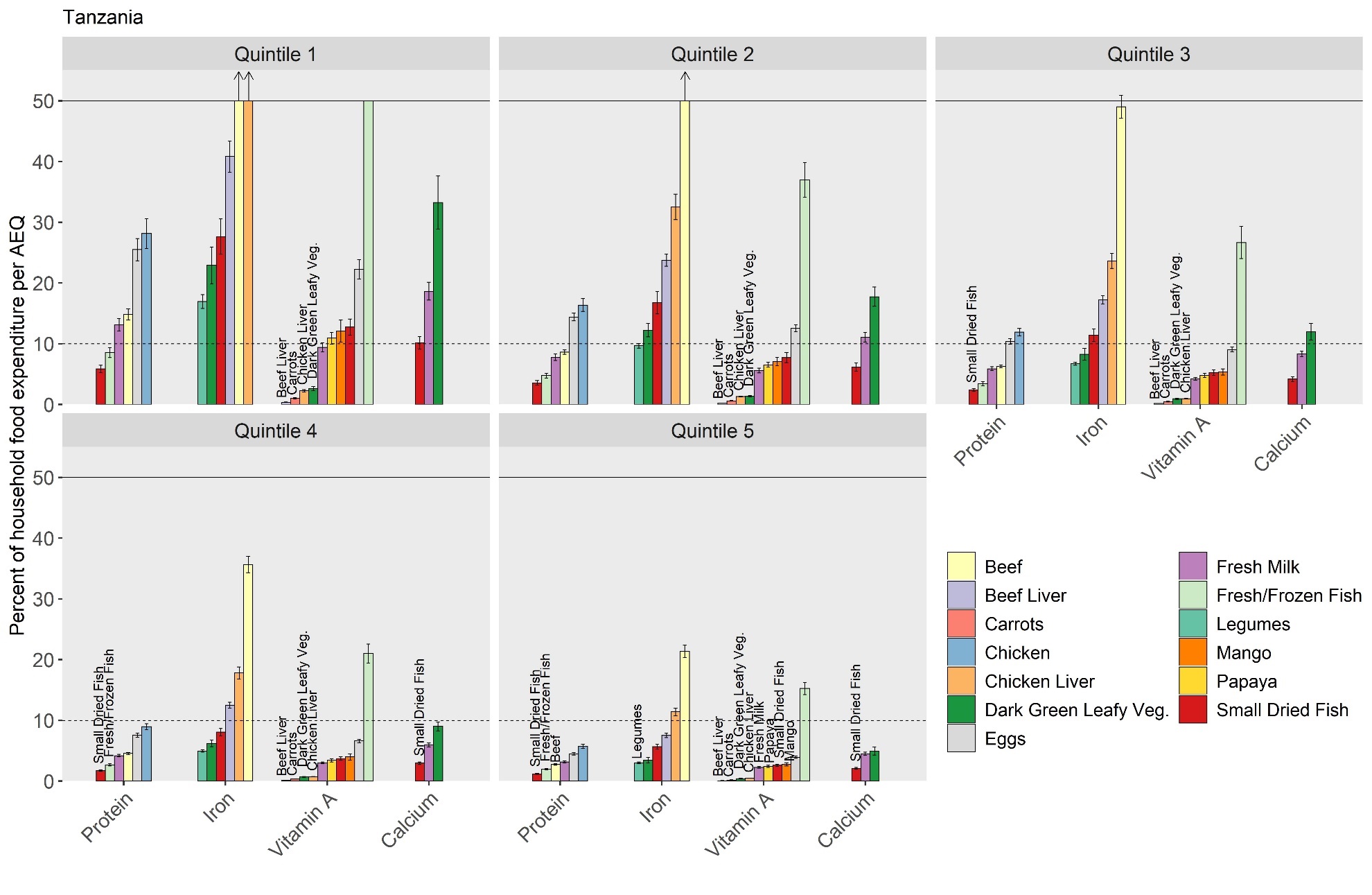

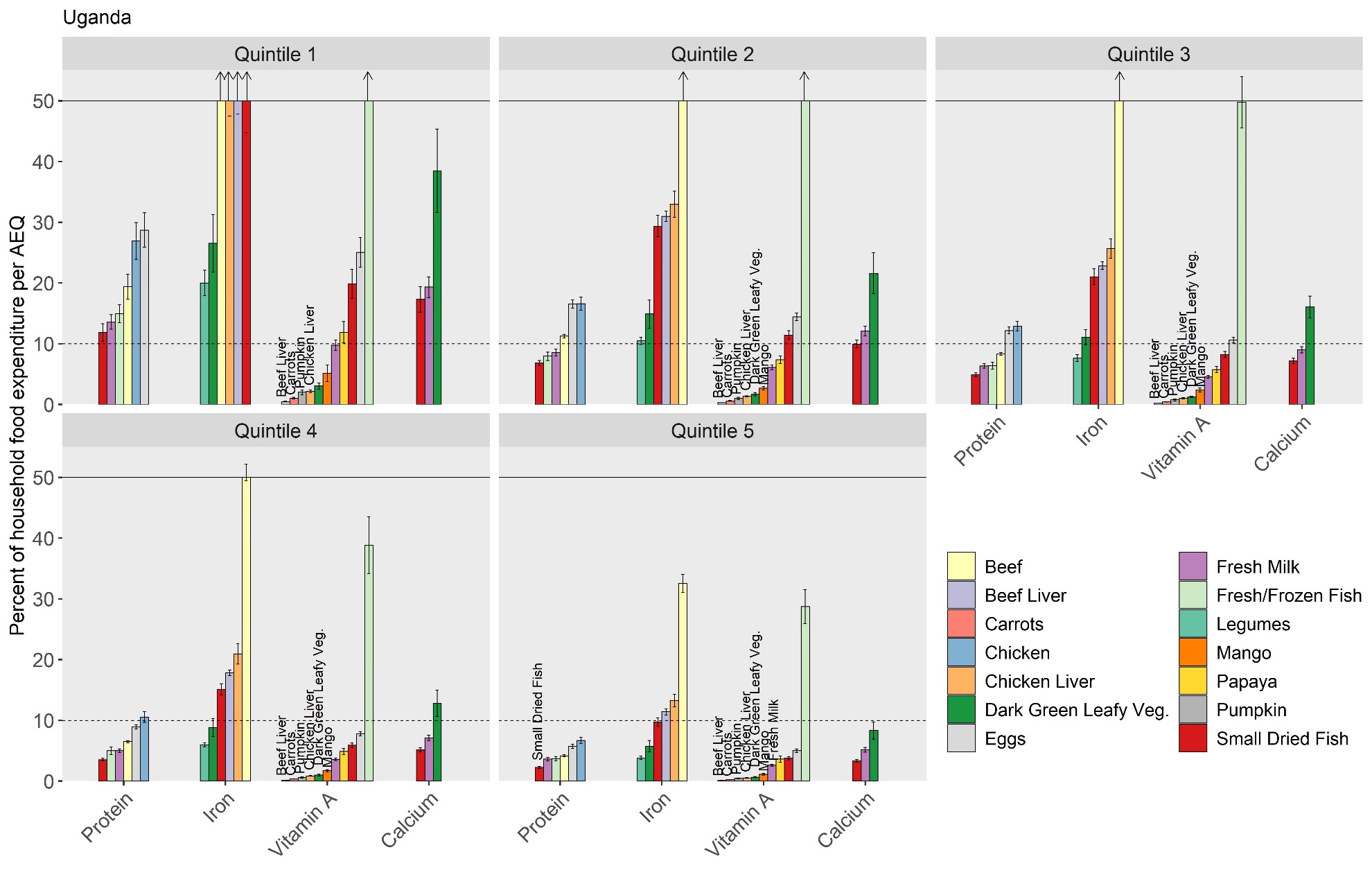

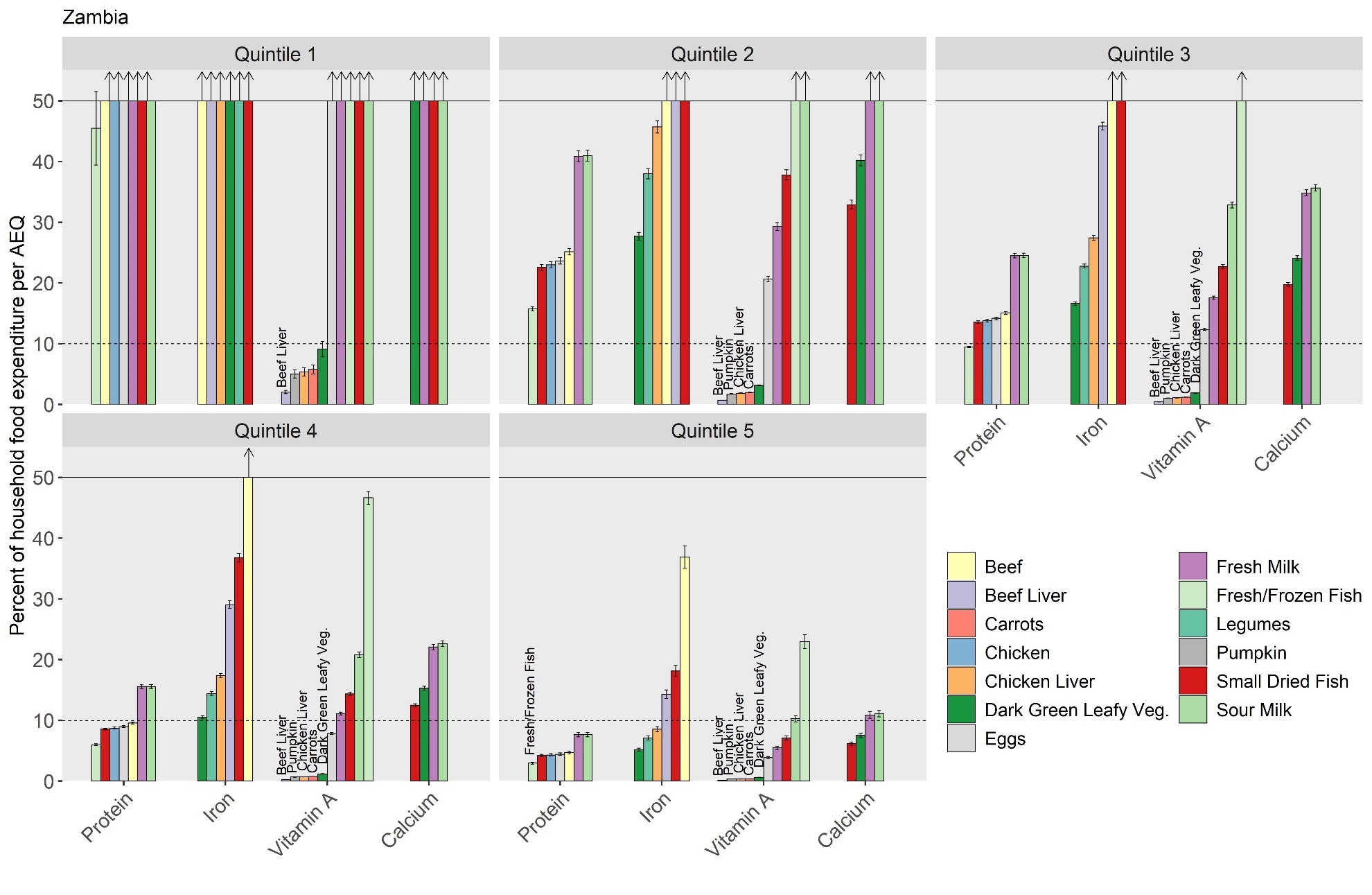

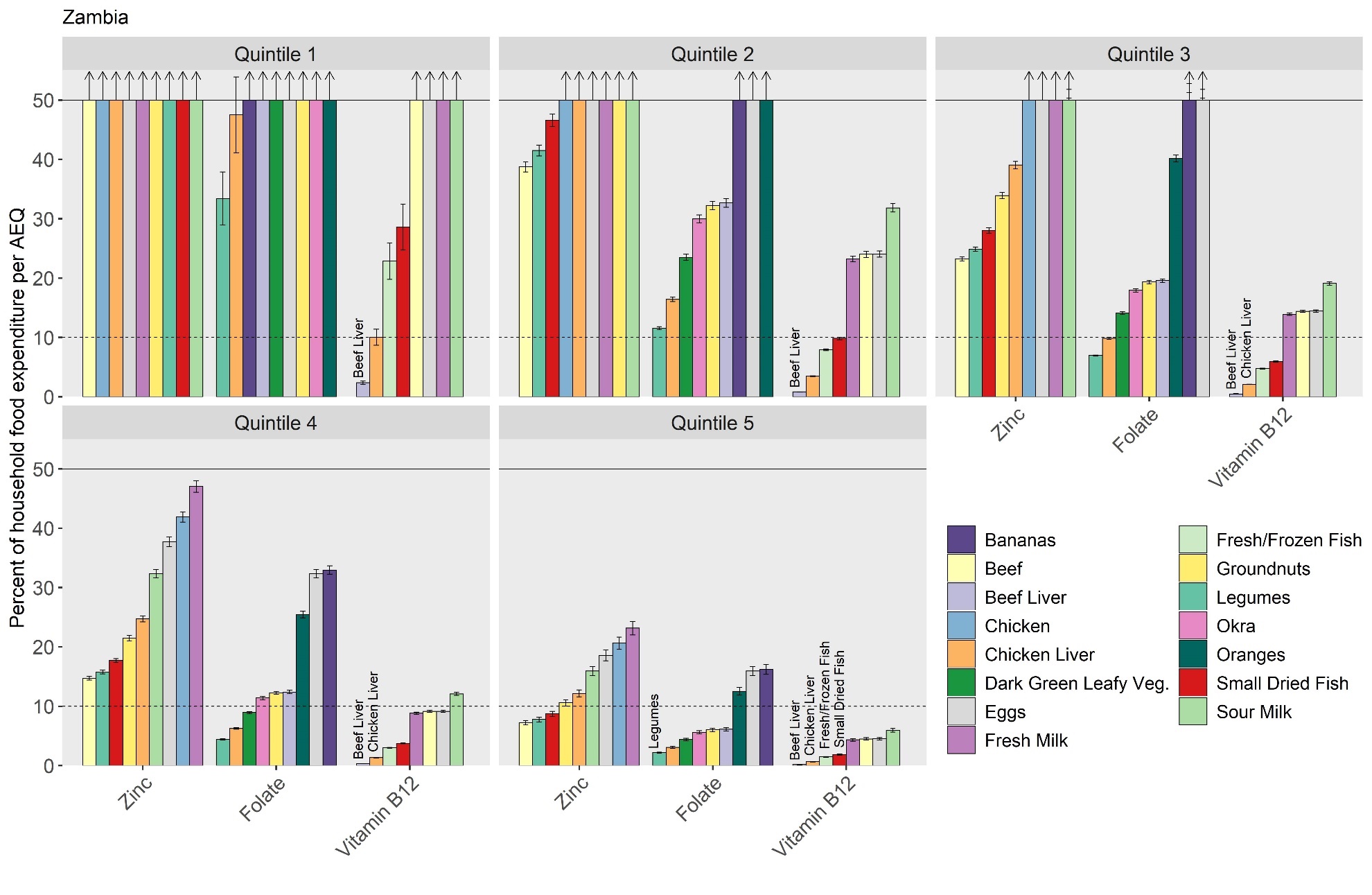


Note: The y-axis was truncated at 50, but the costs of some foods exceeded 50% of household food expenditure per AEQ; these foods are designated with vertical arrows indicating that the bar continues vertically beyond the scale of the graph. Regional price data were not available for Zambia and thus the confidence intervals shown on the Zambia panels do not incorporate geographic price variation.

# Figure S20: Food cost per kcal by quintile


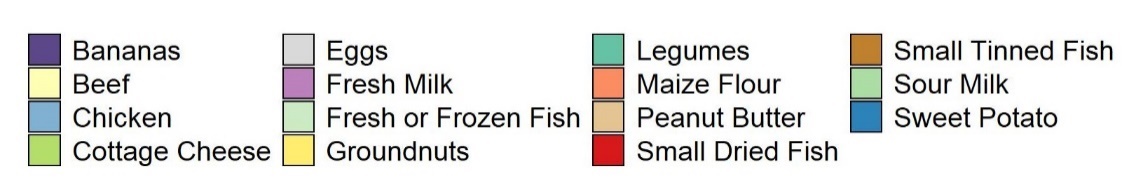


Note: Regional price data were not available for Zambia and thus the confidence intervals shown on the Zambia panels do not incorporate geographic price variation.

Figure S21: Average share of micronutrient requirements affordability analysis, by quintile

Note: Regional price data were not available for Zambia and thus the confidence intervals shown for Zambia do not incorporate geographic price variation.

# Figure S22: Food prices per kg by country, adjusted for currency exchange rates and purchasing power parities

Note: Regional price data were not available for Zambia and thus the confidence intervals shown on the Zambia panels do not incorporate geographic price variation.

# Figure S23: Portion size cost net current expenditure per AEQ, as a share of total household food expenditure per AEQ

Note: The y-axis was truncated at 50, but the costs of some foods exceeded 50% of household food expenditure per AEQ; these foods are designated with vertical arrows indicating that the bar continues vertically beyond the scale of the graph. Regional price data were not available for Zambia and thus the confidence intervals shown on the Zambia panels do not incorporate geographic price variation.

# Figure S24: Sensitivity analysis results for nutrient density and refuse for dark leafy green vegetables, legumes, fish, chicken, and chicken liver

Note: The y-axis was truncated at 50, but the costs of some foods exceeded 50% of household food expenditure per AEQ; these foods are designated with vertical arrows indicating that the bar continues vertically beyond the scale of the graph. Regional price data were not available for Zambia and thus the confidence intervals shown on the Zambia panels do not incorporate geographic price variation.

# Figure S25: Cost per kcal sensitivity analysis results for nutrient density and refuse for legumes, fish, and chicken

Note: Regional price data were not available for Zambia and thus the confidence intervals shown on the Zambia panels do not incorporate geographic price variation.

# Figure 26A: Seasonal price variation by food and country – Ethiopia (Birr)

# Figure 26B: Seasonal price variation by food and country – Mozambique (Metical)

# Figure 26C: Seasonal price variation by food and country – South Africa (Rand)

# Figure 26D: Seasonal price variation by food and country – Tanzania (Shillings)

# Figure 26E: Seasonal price variation by food and country – Uganda (Shillings)

Note: seasonal price variation graphs are not included for Zambia because the Zambia data only covered three months out of the year.

# References

1. Average Food Prices in South Africa (Unofficial).

2. Food Price Monitoring – National Agricultural Marketing Council. Accessed March 14, 2019. https://www.namc.co.za/category/research-publications/publications/food-price-monitoring/

3. Azahan E. Edible Component Parts of Broiler Chickens. *MARDI Research Bulletin*. 1984;12(1):153-156.

4. Ethiopia 2015-16 Household Income Consumption and Expenditure Survey. Accessed April 24, 2019. http://www.csa.gov.et/survey-report/category/7-household-income-consumption-and-expenditure-survey

5. Inquérito sobre Orcamento Familiar (IOF) — Instituto Nacional de Estatistica. Accessed April 3, 2019. http://www.ine.gov.mz/operacoes-estatisticas/inqueritos/inquerito-sobre-orcamento-familiar

6. South Africa - Living Conditions Survey 2014-2015. Accessed March 14, 2019. http://microdata.worldbank.org/index.php/catalog/2882/related_materials

7. Tanzania - National Panel Survey 2014-2015, Wave 4. Accessed February 25, 2019. http://microdata.worldbank.org/index.php/catalog/2862

8. Uganda - National Panel Survey 2013-2014 - Overview. Accessed May 15, 2019. http://microdata.worldbank.org/index.php/catalog/2663/study-description

9. Zambia - Living Conditions Monitoring Survey VII 2015. Accessed February 27, 2019. http://catalog.ihsn.org/index.php/catalog/7105/related_materials

10. Zambia Statistics Agency - Monthly Bulletins. Accessed February 27, 2019. http://www.zamstats.gov.zm/index.php/publications/category/7-2015

11.  World Health Organization, Food and Agriculture Organization of the United Nations, eds. *Vitamin and Mineral Requirements in Human Nutrition*. 2nd ed. World Health Organization ; FAO; 2004. https://apps.who.int/iris/bitstream/handle/10665/42716/9241546123.pdf

12. Dewey KG. Nutrition, Growth, and Complementary Feeding of The Brestfed Infant. *Pediatric Clinics of North America*. 2001;48(1):87-104. doi:10.1016/S0031-3955(05)70287-X

13. Korkalo L, Hauta-alus H, Mutanen M. *Food Composition Tables for Mozambique: Version 2*. Department of Food and Environmental Sciences, University of Helsinki; 2011. Accessed January 26, 2020. https://researchportal.helsinki.fi/en/publications/food-composition-tables-for-mozambique-version-2

14. Nyirenda DB, Musukwa M, Mugode RH, Shindano J. *Zambia Food Composition Tables, 4th Edition*. National Food and Nutrition Commission; 2009. http://nfnc.org.zm/download/file/fid/537

15. Stadlymayr B, Charrondiere UR, Enujiugha VN, et al. *West African Food Composition Table/Table De Composition Des Aliments D’afrique De L’ouest.* Food and Agricultural Organization of the United Nations; 2012. http://www.fao.org/3/a-i2698b.pdf

16.  U.S. Department of Agriculture, Agricultural Research Service. FoodData Central. Published 2019. Accessed January 26, 2020. https://fdc.nal.usda.gov./

17. Lukmanji Z, Hertzmark E, Mlingi N, Assey V, Ndossi G, Fawzi W. *Tanzania Food Composition Tables*. Muhimbili University of Health and Allied Sciences (MUHAS), Tanzania Food and Nutrition Centre (TFNC), Harvard School of Public Health (HSPH); 2008. https://cdn1.sph.harvard.edu/wp-content/uploads/sites/30/2012/10/tanzania-food-composition-tables.pdf

18. Kabahenda MK, Amega R, Okalany E, Husken SMC, Heck S. Protein and micronutrient composition of low value fish products commonly marketed in the Lake Victoria region. *World Journal of Agricultural Sciences*. Published online 2011. Accessed January 26, 2020. https://digitalarchive.worldfishcenter.org/handle/20.500.12348/1196

19. Steiner‐Asiedu M, Lied E, Lie Ø, Nilsen R, Julshamn K. The nutritive value of sun-dried pelagic fish from the rift valley in Africa. *Journal of the Science of Food and Agriculture*. 1993;63(4):439-443. doi:10.1002/jsfa.2740630410

20. World Economic Outlook (October 2018) - Inflation rate, average consumer prices. Accessed April 3, 2019. https://www.imf.org/external/datamapper/PCPIPCH@WEO

21. Official exchange rate (LCU per US$, period average) | Data. Accessed December 10, 2018. https://data.worldbank.org/indicator/PA.NUS.FCRF

22. Bognar A. *Tables on Weight Yield of Food and Retention Factors of Food Constituents for the Calculation of Nutrient Composition of Cooked Foods (Dishes)*. Bundesforschungsanstalt für Ernährung; 2002. http://www.fao.org/uploads/media/bognar_bfe-r-02-03.pdf

23. Roseland JM, Nguyen QA, Williams JR, Patterson KY, Showell B, Pehrsson PR. USDA Table of Cooking Yields for Meat and Poultry. Published online 2014. https://data.nal.usda.gov/dataset/usda-table-cooking-yields-meat-and-poultry

24. Neidecker-Gonzales O, Nestel P, Bouis H. Estimating the Global Costs of Vitamin A Capsule Supplementation: A Review of the Literature: *Food and Nutrition Bulletin*. Published online September 15, 2007. doi:10.1177/156482650702800307

25. Horton S, Blum LS, Diouf M, et al. Delivering Vitamin A Supplements to Children Aged 6–59 Months: Comparing Delivery through Campaigns and through Routine Health Services in Senegal. *Curr Dev Nutr*. 2018;2(4). doi:10.1093/cdn/nzy006

26. Kagin J, Vosti SA, Engle-Stone R, et al. Measuring the Costs of Vitamin A Interventions: Institutional, Spatial, and Temporal Issues in the Context of Cameroon. *Food and Nutrition Bulletin*. Published online September 18, 2015. doi:10.1177/0379572115598445

27. Bhutta ZA, Das JK, Rizvi A, et al. Evidence-based interventions for improvement of maternal and child nutrition: what can be done and at what cost? *Lancet*. 2013;382(9890):452-477. doi:10.1016/S0140-6736(13)60996-4

28. Coverage at a Crossroads: New directions for Vitamin A supplementation programmes. UNICEF DATA. Published May 1, 2018. Accessed September 19, 2020. https://data.unicef.org/resources/vitamin-a-coverage/
